# Supplementary material for: Separate and unequal: Moral domains differ in corresponding social judgments of others
Source: PLoS One. 2026 Jan 8;21(1):e0338026. doi: 10.1371/journal.pone.0338026 (PMC12782401; doi:10.1371/journal.pone.0338026)
Supplement: S1 Appendix — (DOCX) [file pone.0338026.s001.docx]

**S1 Appendix. Study 1 Analyses Not Using Difference Scores.**

**
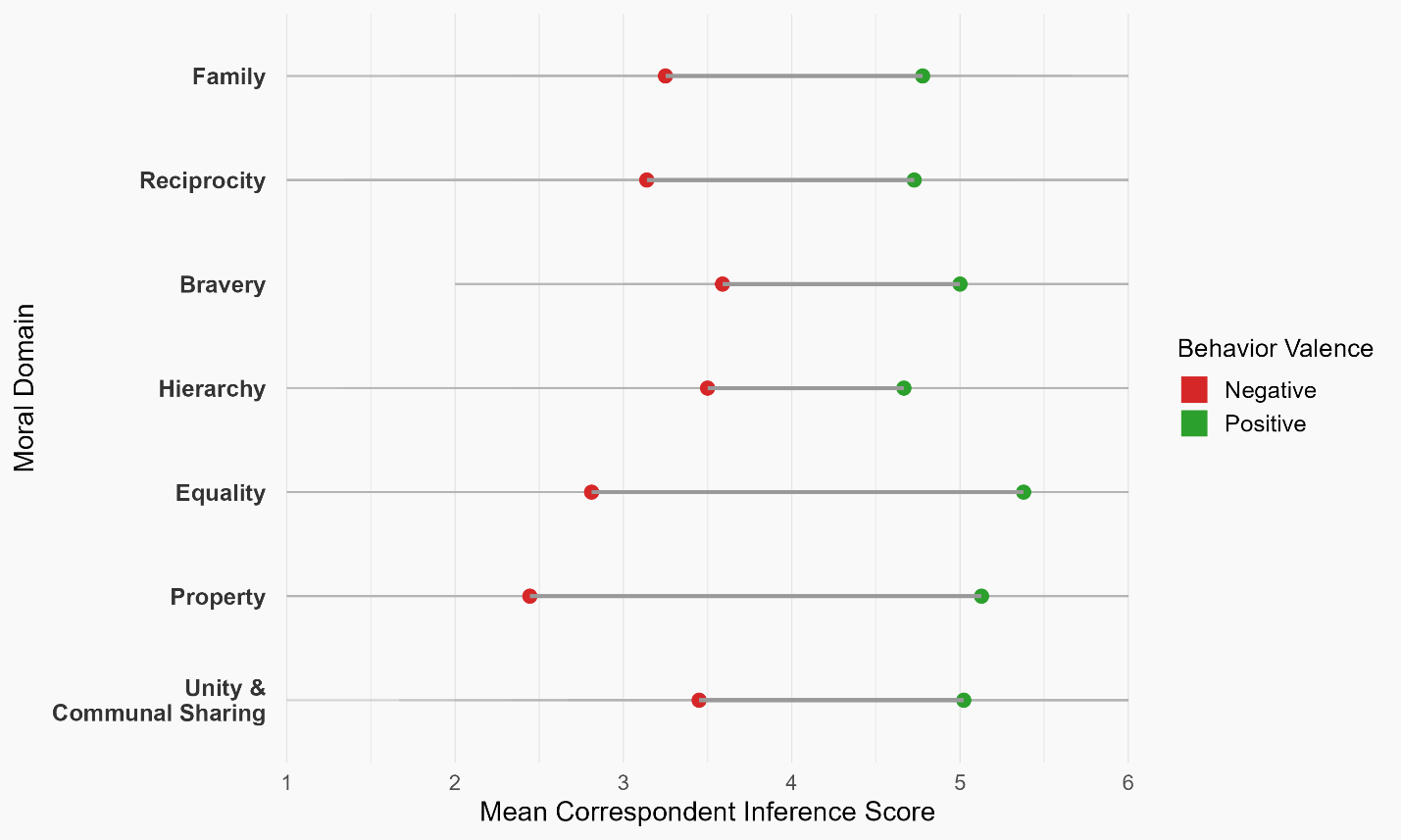
Fig A. Correspondent Inferences by Domain and Valence in Study 1 (No Difference Scores).**

Dumbbell plot shows individual and group-level mean ratings of how principled, ethical, and morally upstanding participants found social targets following positive and negative behaviors in each domain. There was a main effect of valence on correspondent inference scores such that positive behaviors (*M* = 4.96, *SD* = .82) were associated with higher character ratings than negative behaviors (*M* = 3.17, *SD* = 1.05), *F*(1, 59) = 234.62, *η^2^_p_* = .8, *p* = < .001. There was also an interaction between valence and domain, *F*(6, 354) = 20.59, *η^2^_p_* = .26, *p* = < .001, in line with the analysis reported in Study 1: inferences in the *Equality* and *Property* domains were more sensitive to behavior valence such that there were larger differences in inferences following positive and negative behaviors. There were no patterns to suggest the effects shown by difference scores in Study 1 were driven solely by positive or negative behaviors. Bonferroni-adjusted multiple comparisons of ratings across domains by behavior valence are shown in Table A below.

**Table A. Multiple Comparisons of Correspondent Inference by Valence in Study 1 (No Difference Scores).**

|  | | | | | | | |
| --- | --- | --- | --- | --- | --- | --- | --- |
| Valence | (I) Domain | (J) Domain | Mean Difference (I-J) | Std. Error | 95% Confidence Interval for Difference^b^ | |  |
|  |  |  |  |  | Lower Bound | Upper Bound |  |
| Positive | Family | Reciprocity | .05 | .12 | -.34 | .44 |  |
|  |  | Bravery | -.22 | .12 | -.59 | .15 |  |
|  |  | Hierarchy | .11 | .11 | -.25 | .47 |  |
|  |  | Equality | **-.60^***^** | .10 | -.92 | -.28 |  |
|  |  | Property | **-.35^*^** | .09 | -.65 | -.05 |  |
|  |  | Unity & Communal Sharing | -.24 | .11 | -.58 | .09 |  |
|  | Reciprocity | Family | -.05 | .12 | -.44 | .34 |  |
|  |  | Bravery | -.27 | .11 | -.64 | .09 |  |
|  |  | Hierarchy | .06 | .14 | -.38 | .51 |  |
|  |  | Equality | **-.65^***^** | .14 | -1.09 | -.21 |  |
|  |  | Property | **-.40^*^** | .11 | -.76 | -.04 |  |
|  |  | Unity & Communal Sharing | -.29 | .11 | -.66 | .07 |  |
|  | Bravery | Family | .22 | .12 | -.15 | .59 |  |
|  |  | Reciprocity | .27 | .11 | -.09 | .64 |  |
|  |  | Hierarchy | .33 | .14 | -.12 | .79 |  |
|  |  | Equality | **-.38^*^** | .11 | -.74 | -.02 |  |
|  |  | Property | -.13 | .09 | -.41 | .15 |  |
|  |  | Unity & Communal Sharing | -.02 | .11 | -.36 | .32 |  |
|  | Hierarchy | Family | -.11 | .11 | -.47 | .25 |  |
|  |  | Reciprocity | -.06 | .14 | -.51 | .38 |  |
|  |  | Bravery | -.33 | .14 | -.79 | .12 |  |
|  |  | Equality | **-.71^***^** | .12 | -1.10 | -.32 |  |
|  |  | Property | **-.46^*^** | .13 | -.86 | -.06 |  |
|  |  | Unity & Communal Sharing | -.36^*^ | .10 | -.68 | -.03 |  |
|  | Equality | Family | **.60^***^** | .10 | .28 | .92 |  |
|  |  | Reciprocity | **.65^***^** | .14 | .21 | 1.09 |  |
|  |  | Bravery | **.38^*^** | .11 | .02 | .74 |  |
|  |  | Hierarchy | **.71^***^** | .12 | .32 | 1.10 |  |
|  |  | Property | .25 | .11 | -.09 | .59 |  |
|  |  | Unity & Communal Sharing | **.36^*^** | .10 | .03 | .68 |  |
|  | Property | Family | **.35^*^** | .09 | .05 | .65 |  |
|  |  | Reciprocity | **.40^*^** | .11 | .04 | .76 |  |
|  |  | Bravery | .13 | .09 | -.15 | .41 |  |
|  |  | Hierarchy | **.46^*^** | .13 | .06 | .86 |  |
|  |  | Equality | -.25 | .11 | -.59 | .09 |  |
|  |  | Unity & Communal Sharing | .11 | .10 | -.20 | .41 |  |
|  | Unity & Communal Sharing | Family | .24 | .11 | -.09 | .58 |  |
|  |  | Reciprocity | .29 | .11 | -.07 | .66 |  |
|  |  | Bravery | .02 | .11 | -.32 | .36 |  |
|  |  | Hierarchy | **.36^*^** | .10 | .03 | .68 |  |
|  |  | Equality | **-.36^*^** | .10 | -.68 | -.03 |  |
|  |  | Property | -.11 | .10 | -.41 | .20 |  |
| Negative | Family | Reciprocity | .11 | .12 | -.26 | .48 |  |
|  |  | Bravery | -.34 | .11 | -.69 | .02 |  |
|  |  | Hierarchy | -.25 | .13 | -.67 | .17 |  |
|  |  | Equality | **.44^*^** | .13 | .03 | .85 |  |
|  |  | Property | **.81^***^** | .14 | .37 | 1.25 |  |
|  |  | Unity & Communal Sharing | -.20 | .12 | -.59 | .19 |  |
|  | Reciprocity | Family | -.11 | .12 | -.48 | .26 |  |
|  |  | Bravery | **-.45^*^** | .13 | -.88 | -.02 |  |
|  |  | Hierarchy | -.36 | .12 | -.75 | .02 |  |
|  |  | Equality | .33 | .16 | -.17 | .83 |  |
|  |  | Property | **.69^***^** | .13 | .28 | 1.11 |  |
|  |  | Unity & Communal Sharing | -.31 | .16 | -.80 | .18 |  |
|  | Bravery | Family | .34 | .11 | -.02 | .69 |  |
|  |  | Reciprocity | **.45^*^** | .13 | .02 | .88 |  |
|  |  | Hierarchy | .09 | .13 | -.34 | .52 |  |
|  |  | Equality | **.78^***^** | .15 | .29 | 1.26 |  |
|  |  | Property | **1.14^***^** | .14 | .69 | 1.60 |  |
|  |  | Unity & Communal Sharing | .14 | .12 | -.23 | .51 |  |
|  | Hierarchy | Family | .25 | .13 | -.17 | .67 |  |
|  |  | Reciprocity | .36 | .12 | -.02 | .75 |  |
|  |  | Bravery | -.09 | .13 | -.52 | .34 |  |
|  |  | Equality | **.69^***^** | .15 | .22 | 1.16 |  |
|  |  | Property | **1.06^***^** | .14 | .61 | 1.51 |  |
|  |  | Unity & Communal Sharing | .05 | .13 | -.38 | .48 |  |
|  | Equality | Family | **-.44^*^** | .13 | -.85 | -.03 |  |
|  |  | Reciprocity | -.33 | .16 | -.83 | .17 |  |
|  |  | Bravery | **-.78^***^** | .15 | -1.26 | -.29 |  |
|  |  | Hierarchy | **-.69^***^** | .15 | -1.16 | -.22 |  |
|  |  | Property | .37 | .15 | -.12 | .86 |  |
|  |  | Unity & Communal Sharing | **-.64^***^** | .14 | -1.08 | -.20 |  |
|  | Property | Family | **-.81^***^** | .14 | -1.25 | -.37 |  |
|  |  | Reciprocity | **-.69^***^** | .13 | -1.11 | -.28 |  |
|  |  | Bravery | **-1.14^***^** | .14 | -1.60 | -.69 |  |
|  |  | Hierarchy | **-1.06^***^** | .14 | -1.51 | -.61 |  |
|  |  | Equality | -.37 | .15 | -.86 | .12 |  |
|  |  | Unity & Communal Sharing | **-1.01^***^** | .15 | -1.49 | -.53 |  |
|  | Unity & Communal Sharing | Family | .20 | .12 | -.19 | .59 |  |
|  |  | Reciprocity | .31 | .16 | -.18 | .80 |  |
|  |  | Bravery | -.14 | .12 | -.51 | .23 |  |
|  |  | Hierarchy | -.05 | .13 | -.48 | .38 |  |
|  |  | Equality | **.64^***^** | .14 | .20 | 1.08 |  |
|  |  | Property | **1.01^***^** | .15 | .53 | 1.49 |  |
| Based on estimated marginal means | | | | | | | |
| *. The mean difference is significant at the .05 level. ***. The mean difference is significant at the .001 level. | | | | | | | |
| b. Adjustment for multiple comparisons: Bonferroni. | | | | | | | |

**Fig B. Dispositional and Situational Attributions by Domain and Valence in Study 1 (No Difference Scores).**

**
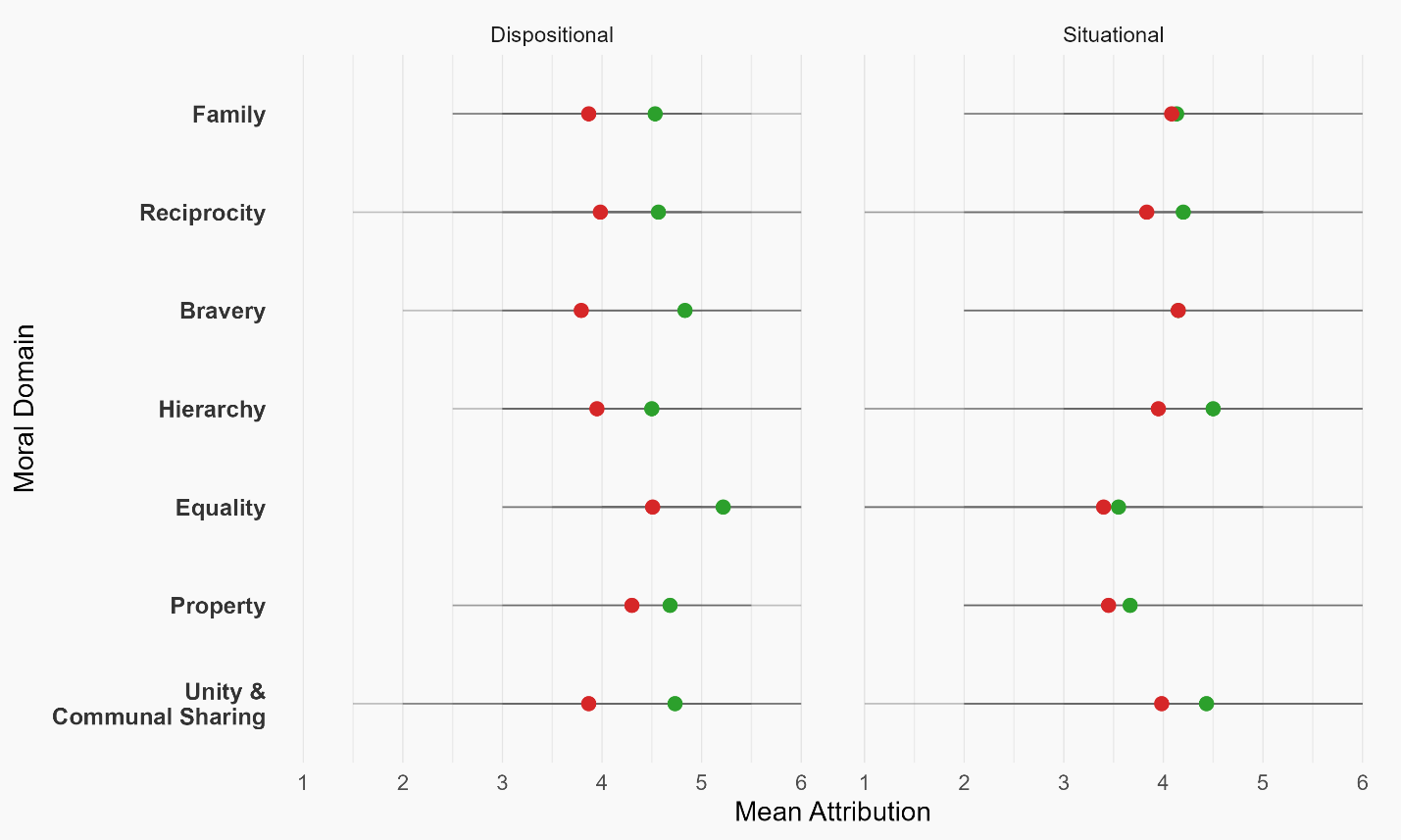
**

Dumbbell plot shows individual and group-level mean ratings of dispositional attribution and situational attribution following positive and negative behaviors in each domain. Participants more readily made judgments of attribution (both dispositional and situational) following positive behaviors. There were main effects of domain such that behaviors related to *Equality* and *Property* statistically differed from other domains: When collapsed across valence, these behaviors were more dispositionally attributed and less situationally attributed, *F_dispositional_* (4.83, 280.38) = 19.18, *η^2^_p_* = .25, *p* < .001, *F_situational_* (5.16, 304.46) = 15.59, *η^2^_p_* = .21, *p* < .001. There were also interaction effects between domain and valence for both types of attribution, showing that the effect of behavior valence on attribution statistically differed across domains, *F_dispositional_* (6, 354) = 2.12, *η^2^_p_* = .04, *p* = .05, *F_situational_* (5.58, 323.63) = 6.11, *η^2^_p_* = .1, *p* < .001. Bonferroni-adjusted multiple comparisons for these analyses show the directions of these trends and are found in Tables B and C below.

**Table B. Multiple Comparisons of Dispositional Attribution in Study 1 (No Difference Scores).**

| Valence | (I) Domain | (J) Domain | Mean Difference (I-J) | Std. Error | 95% Confidence Interval for Difference^b^ | |  |
| --- | --- | --- | --- | --- | --- | --- | --- |
|  |  |  |  |  | Lower Bound | Upper Bound |  |
| Positive | Family | Reciprocity | -.03 | .10 | -.35 | .30 |  |
|  |  | Bravery | **-.30^*^** | .08 | -.54 | -.05 |  |
|  |  | Hierarchy | .04 | .08 | -.22 | .31 |  |
|  |  | Equality | **-.69^***^** | .08 | -.94 | -.43 |  |
|  |  | Property | -.14 | .07 | -.37 | .08 |  |
|  |  | Unity & Communal Sharing | -.20 | .09 | -.49 | .08 |  |
|  | Reciprocity | Family | .03 | .10 | -.30 | .35 |  |
|  |  | Bravery | -.27 | .11 | -.61 | .07 |  |
|  |  | Hierarchy | .07 | .10 | -.26 | .39 |  |
|  |  | Equality | **-.66^***^** | .11 | -1.01 | -.31 |  |
|  |  | Property | -.12 | .10 | -.44 | .20 |  |
|  |  | Unity & Communal Sharing | -.18 | .12 | -.56 | .21 |  |
|  | Bravery | Family | **.30^*^** | .08 | .05 | .54 |  |
|  |  | Reciprocity | .27 | .11 | -.07 | .61 |  |
|  |  | Hierarchy | **.34^*^** | .09 | .06 | .62 |  |
|  |  | Equality | **-.39^***^** | .08 | -.64 | -.13 |  |
|  |  | Property | .15 | .09 | -.13 | .44 |  |
|  |  | Unity & Communal Sharing | .09 | .08 | -.16 | .35 |  |
|  | Hierarchy | Family | -.04 | .08 | -.31 | .22 |  |
|  |  | Reciprocity | -.07 | .10 | -.39 | .26 |  |
|  |  | Bravery | **-.34^*^** | .09 | -.62 | -.06 |  |
|  |  | Equality | **-.73^***^** | .09 | -1.03 | -.43 |  |
|  |  | Property | -.19 | .09 | -.46 | .09 |  |
|  |  | Unity & Communal Sharing | -.25 | .08 | -.51 | .02 |  |
|  | Equality | Family | **.69^***^** | .08 | .43 | .94 |  |
|  |  | Reciprocity | **.66^***^** | .11 | .31 | 1.01 |  |
|  |  | Bravery | **.39^***^** | .08 | .13 | .64 |  |
|  |  | Hierarchy | **.73^***^** | .09 | .43 | 1.03 |  |
|  |  | Property | **.54^***^** | .09 | .25 | .84 |  |
|  |  | Unity & Communal Sharing | **.48^***^** | .09 | .21 | .76 |  |
|  | Property | Family | .14 | .07 | -.08 | .37 |  |
|  |  | Reciprocity | .12 | .10 | -.20 | .44 |  |
|  |  | Bravery | -.15 | .09 | -.44 | .13 |  |
|  |  | Hierarchy | .19 | .09 | -.09 | .46 |  |
|  |  | Equality | **-.54^***^** | .09 | -.84 | -.25 |  |
|  |  | Unity & Communal Sharing | -.06 | .09 | -.36 | .24 |  |
|  | Unity & Communal Sharing | Family | .20 | .09 | -.08 | .49 |  |
|  |  | Reciprocity | .18 | .12 | -.21 | .56 |  |
|  |  | Bravery | -.09 | .08 | -.35 | .16 |  |
|  |  | Hierarchy | .25 | .08 | -.02 | .51 |  |
|  |  | Equality | **-.48^***^** | .09 | -.76 | -.21 |  |
|  |  | Property | .06 | .09 | -.24 | .36 |  |
| Negative | Family | Reciprocity | -.13 | .09 | -.42 | .16 |  |
|  |  | Bravery | .07 | .10 | -.24 | .38 |  |
|  |  | Hierarchy | -.09 | .09 | -.37 | .19 |  |
|  |  | Equality | **-.66^***^** | .12 | -1.04 | -.29 |  |
|  |  | Property | **-.45^***^** | .08 | -.71 | -.18 |  |
|  |  | Unity & Communal Sharing | .00 | .10 | -.32 | .32 |  |
|  | Reciprocity | Family | .13 | .09 | -.16 | .42 |  |
|  |  | Bravery | .19 | .10 | -.14 | .53 |  |
|  |  | Hierarchy | .03 | .10 | -.27 | .34 |  |
|  |  | Equality | **-.53^*^** | .14 | -.98 | -.08 |  |
|  |  | Property | **-.32^*^** | .09 | -.61 | -.04 |  |
|  |  | Unity & Communal Sharing | .13 | .12 | -.24 | .49 |  |
|  | Bravery | Family | -.07 | .10 | -.38 | .24 |  |
|  |  | Reciprocity | -.19 | .10 | -.53 | .14 |  |
|  |  | Hierarchy | -.16 | .10 | -.48 | .15 |  |
|  |  | Equality | **-.73^***^** | .11 | -1.09 | -.37 |  |
|  |  | Property | **-.52^***^** | .09 | -.80 | -.24 |  |
|  |  | Unity & Communal Sharing | -.07 | .11 | -.43 | .29 |  |
|  | Hierarchy | Family | .09 | .09 | -.19 | .37 |  |
|  |  | Reciprocity | -.03 | .10 | -.34 | .27 |  |
|  |  | Bravery | .16 | .10 | -.15 | .48 |  |
|  |  | Equality | **-.57^***^** | .14 | -1.00 | -.14 |  |
|  |  | Property | **-.36^*^** | .10 | -.66 | -.05 |  |
|  |  | Unity & Communal Sharing | .09 | .10 | -.22 | .41 |  |
|  | Equality | Family | **.66^***^** | .12 | .29 | 1.04 |  |
|  |  | Reciprocity | **.53^*^** | .14 | .08 | .98 |  |
|  |  | Bravery | **.73^***^** | .11 | .37 | 1.09 |  |
|  |  | Hierarchy | **.57^***^** | .14 | .14 | 1.00 |  |
|  |  | Property | .21 | .11 | -.15 | .57 |  |
|  |  | Unity & Communal Sharing | **.66^***^** | .12 | .28 | 1.04 |  |
|  | Property | Family | **.45^***^** | .08 | .18 | .71 |  |
|  |  | Reciprocity | **.32^*^** | .09 | .04 | .61 |  |
|  |  | Bravery | **.52^***^** | .09 | .24 | .80 |  |
|  |  | Hierarchy | **.36^*^** | .10 | .05 | .66 |  |
|  |  | Equality | -.21 | .11 | -.57 | .15 |  |
|  |  | Unity & Communal Sharing | **.45^***^** | .09 | .15 | .75 |  |
|  | Unity & Communal Sharing | Family | .00 | .10 | -.32 | .32 |  |
|  |  | Reciprocity | -.13 | .12 | -.49 | .24 |  |
|  |  | Bravery | .07 | .11 | -.29 | .43 |  |
|  |  | Hierarchy | -.09 | .10 | -.41 | .22 |  |
|  |  | Equality | **-.66^***^** | .12 | -1.04 | -.28 |  |
|  |  | Property | **-.45^***^** | .09 | -.75 | -.15 |  |
| Based on estimated marginal means | | | | | | | |
| *. The mean difference is significant at the .05 level. ***. The mean difference is significant at the .001 level. | | | | | | | |
| b. Adjustment for multiple comparisons: Bonferroni. | | | | | | | |

**Table C. Multiple Comparisons of Situational Attribution in Study 1 (No Difference Scores).**

| Valence | (I) Domain | (J) Domain | Mean Difference (I-J) | Std. Error | 95% Confidence Interval for Difference^b^ | |  |
| --- | --- | --- | --- | --- | --- | --- | --- |
|  |  |  |  |  | Lower Bound | Upper Bound |  |
| Positive | Family | Reciprocity | -.07 | .14 | -.51 | .37 |  |
|  |  | Bravery | -.02 | .12 | -.40 | .36 |  |
|  |  | Hierarchy | -.37 | .12 | -.76 | .03 |  |
|  |  | Equality | **.58^***^** | .14 | .14 | 1.03 |  |
|  |  | Property | **.47^*^** | .14 | .03 | .90 |  |
|  |  | Unity & Communal Sharing | -.30 | .15 | -.78 | .18 |  |
|  | Reciprocity | Family | .07 | .14 | -.37 | .51 |  |
|  |  | Bravery | .05 | .16 | -.46 | .56 |  |
|  |  | Hierarchy | -.30 | .15 | -.77 | .17 |  |
|  |  | Equality | **.65^*^** | .18 | .09 | 1.21 |  |
|  |  | Property | .53 | .18 | -.04 | 1.11 |  |
|  |  | Unity & Communal Sharing | -.23 | .16 | -.74 | .28 |  |
|  | Bravery | Family | .02 | .12 | -.36 | .40 |  |
|  |  | Reciprocity | -.05 | .16 | -.56 | .46 |  |
|  |  | Hierarchy | -.35 | .14 | -.80 | .10 |  |
|  |  | Equality | **.60^*^** | .16 | .10 | 1.10 |  |
|  |  | Property | **.48^*^** | .15 | .00 | .96 |  |
|  |  | Unity & Communal Sharing | -.28 | .13 | -.70 | .14 |  |
|  | Hierarchy | Family | .37 | .12 | -.03 | .76 |  |
|  |  | Reciprocity | .30 | .15 | -.17 | .77 |  |
|  |  | Bravery | .35 | .14 | -.10 | .80 |  |
|  |  | Equality | **.95^***^** | .18 | .39 | 1.51 |  |
|  |  | Property | **.83^***^** | .18 | .27 | 1.40 |  |
|  |  | Unity & Communal Sharing | .07 | .16 | -.43 | .56 |  |
|  | Equality | Family | **-.58^***^** | .14 | -1.03 | -.14 |  |
|  |  | Reciprocity | **-.65^*^** | .18 | -1.21 | -.09 |  |
|  |  | Bravery | **-.60^*^** | .16 | -1.10 | -.10 |  |
|  |  | Hierarchy | **-.95^***^** | .18 | -1.51 | -.39 |  |
|  |  | Property | -.12 | .13 | -.52 | .29 |  |
|  |  | Unity & Communal Sharing | **-.88^***^** | .16 | -1.39 | -.38 |  |
|  | Property | Family | **-.47^*^** | .14 | -.90 | -.03 |  |
|  |  | Reciprocity | -.53 | .18 | -1.11 | .04 |  |
|  |  | Bravery | **-.48^*^** | .15 | -.96 | .00 |  |
|  |  | Hierarchy | **-.83^***^** | .18 | -1.40 | -.27 |  |
|  |  | Equality | .12 | .13 | -.29 | .52 |  |
|  |  | Unity & Communal Sharing | **-.77^***^** | .17 | -1.30 | -.24 |  |
|  | Unity & Communal Sharing | Family | .30 | .15 | -.18 | .78 |  |
|  |  | Reciprocity | .23 | .16 | -.28 | .74 |  |
|  |  | Bravery | .28 | .13 | -.14 | .70 |  |
|  |  | Hierarchy | -.07 | .16 | -.56 | .43 |  |
|  |  | Equality | **.88^***^** | .16 | .38 | 1.39 |  |
|  |  | Property | **.77^***^** | .17 | .24 | 1.30 |  |
| Negative | Family | Reciprocity | .25 | .15 | -.23 | .73 |  |
|  |  | Bravery | -.07 | .15 | -.53 | .40 |  |
|  |  | Hierarchy | .13 | .15 | -.36 | .63 |  |
|  |  | Equality | **.68^***^** | .13 | .27 | 1.09 |  |
|  |  | Property | **.63^*^** | .17 | .09 | 1.18 |  |
|  |  | Unity & Communal Sharing | .10 | .15 | -.37 | .57 |  |
|  | Reciprocity | Family | -.25 | .15 | -.73 | .23 |  |
|  |  | Bravery | -.32 | .14 | -.76 | .13 |  |
|  |  | Hierarchy | -.12 | .13 | -.54 | .31 |  |
|  |  | Equality | .43 | .15 | -.05 | .91 |  |
|  |  | Property | .38 | .14 | -.05 | .82 |  |
|  |  | Unity & Communal Sharing | -.15 | .15 | -.61 | .31 |  |
|  | Bravery | Family | .07 | .15 | -.40 | .53 |  |
|  |  | Reciprocity | .32 | .14 | -.13 | .76 |  |
|  |  | Hierarchy | .20 | .14 | -.23 | .63 |  |
|  |  | Equality | **.75^***^** | .15 | .29 | 1.21 |  |
|  |  | Property | **.70^***^** | .16 | .19 | 1.21 |  |
|  |  | Unity & Communal Sharing | .17 | .13 | -.25 | .59 |  |
|  | Hierarchy | Family | -.13 | .15 | -.63 | .36 |  |
|  |  | Reciprocity | .12 | .13 | -.31 | .54 |  |
|  |  | Bravery | -.20 | .14 | -.63 | .23 |  |
|  |  | Equality | **.55^*^** | .16 | .03 | 1.07 |  |
|  |  | Property | .50 | .17 | -.03 | 1.03 |  |
|  |  | Unity & Communal Sharing | -.03 | .14 | -.48 | .41 |  |
|  | Equality | Family | **-.68^***^** | .13 | -1.09 | -.27 |  |
|  |  | Reciprocity | -.43 | .15 | -.91 | .05 |  |
|  |  | Bravery | **-.75^***^** | .15 | -1.21 | -.29 |  |
|  |  | Hierarchy | **-.55^*^** | .16 | -1.07 | -.03 |  |
|  |  | Property | -.05 | .16 | -.55 | .45 |  |
|  |  | Unity & Communal Sharing | **-.58^*^** | .15 | -1.07 | -.10 |  |
|  | Property | Family | **-.63^*^** | .17 | -1.18 | -.09 |  |
|  |  | Reciprocity | -.38 | .14 | -.82 | .05 |  |
|  |  | Bravery | **-.70^***^** | .16 | -1.21 | -.19 |  |
|  |  | Hierarchy | -.50 | .17 | -1.03 | .03 |  |
|  |  | Equality | .05 | .16 | -.45 | .55 |  |
|  |  | Unity & Communal Sharing | **-.53^*^** | .15 | -1.00 | -.07 |  |
|  | Unity & Communal Sharing | Family | -.10 | .15 | -.57 | .37 |  |
|  |  | Reciprocity | .15 | .15 | -.31 | .61 |  |
|  |  | Bravery | -.17 | .13 | -.59 | .25 |  |
|  |  | Hierarchy | .03 | .14 | -.41 | .48 |  |
|  |  | Equality | **.58^*^** | .15 | .10 | 1.07 |  |
|  |  | Property | **.53^*^** | .15 | .07 | 1.00 |  |
| Based on estimated marginal means | | | | | | | |
| *. The mean difference is significant at the .05 level. ***. The mean difference is significant at the .001 level. | | | | | | | |
| b. Adjustment for multiple comparisons: Bonferroni. | | | | | | | |
