# Supplementary material for: Separate and unequal: Moral domains differ in corresponding social judgments of others
Source: PLoS One. 2026 Jan 8;21(1):e0338026. doi: 10.1371/journal.pone.0338026 (PMC12782401; doi:10.1371/journal.pone.0338026)
Supplement: S2 Appendix — (DOCX) [file pone.0338026.s002.docx]

**S2 Appendix. Multiple Comparisons for Reported Analyses in Study 1**

**Table A. Multiple Comparisons of Study 1 Correspondent Inference Difference Scores.**

| (I) Domain | (J) Domain | Mean Difference (I-J) | Std. Error | 95% Confidence Interval for Difference^b^ | |  |
| --- | --- | --- | --- | --- | --- | --- |
|  |  |  |  | Lower Bound | Upper Bound |  |
| Family | Reciprocity | -.06 | .15 | -.54 | .42 |  |
|  | Bravery | .12 | .17 | -.43 | .66 |  |
|  | Hierarchy | .36 | .18 | -.22 | .94 |  |
|  | Equality | **-1.04^***^** | .17 | -1.56 | -.51 |  |
|  | Property | **-1.16^***^** | .16 | -1.65 | -.66 |  |
|  | Unity & Communal Sharing | -.04 | .17 | -.58 | .49 |  |
| Reciprocity | Family | .06 | .15 | -.42 | .54 |  |
|  | Bravery | .18 | .17 | -.37 | .73 |  |
|  | Hierarchy | .42 | .18 | -.16 | 1.01 |  |
|  | Equality | **-.98^***^** | .21 | -1.66 | -.30 |  |
|  | Property | **-1.09^***^** | .17 | -1.63 | -.56 |  |
|  | Unity & Communal Sharing | .02 | .20 | -.61 | .64 |  |
| Bravery | Family | -.12 | .17 | -.66 | .43 |  |
|  | Reciprocity | -.18 | .17 | -.73 | .37 |  |
|  | Hierarchy | .24 | .22 | -.45 | .94 |  |
|  | Equality | **-1.16^***^** | .20 | -1.80 | -.52 |  |
|  | Property | **-1.27^***^** | .17 | -1.82 | -.72 |  |
|  | Unity & Communal Sharing | -.16 | .19 | -.75 | .43 |  |
| Hierarchy | Family | -.36 | .18 | -.94 | .22 |  |
|  | Reciprocity | -.42 | .18 | -1.01 | .16 |  |
|  | Bravery | -.24 | .22 | -.94 | .45 |  |
|  | Equality | **-1.40^***^** | .21 | -2.05 | -.75 |  |
|  | Property | **-1.52^***^** | .18 | -2.09 | -.95 |  |
|  | Unity & Communal Sharing | -.41 | .18 | -.99 | .17 |  |
| Equality | Family | **1.04^***^** | .17 | .51 | 1.56 |  |
|  | Reciprocity | **.98^***^** | .21 | .30 | 1.66 |  |
|  | Bravery | **1.16^***^** | .20 | .52 | 1.80 |  |
|  | Hierarchy | **1.40^***^** | .21 | .75 | 2.05 |  |
|  | Property | -.12 | .19 | -.73 | .49 |  |
|  | Unity & Communal Sharing | **.99^***^** | .19 | .40 | 1.59 |  |
| Property | Family | **1.16^***^** | .16 | .66 | 1.65 |  |
|  | Reciprocity | **1.09^***^** | .17 | .56 | 1.63 |  |
|  | Bravery | **1.27^***^** | .17 | .72 | 1.82 |  |
|  | Hierarchy | **1.52^***^** | .18 | .95 | 2.09 |  |
|  | Equality | .12 | .19 | -.49 | .73 |  |
|  | Unity & Communal Sharing | **1.11^***^** | .19 | .52 | 1.71 |  |
| Unity & Communal Sharing | Family | .04 | .17 | -.49 | .58 |  |
|  | Reciprocity | -.02 | .20 | -.64 | .61 |  |
|  | Bravery | .16 | .19 | -.43 | .75 |  |
|  | Hierarchy | .41 | .18 | -.17 | .99 |  |
|  | Equality | **-.99^***^** | .19 | -1.59 | -.40 |  |
|  | Property | **-1.11^***^** | .19 | -1.71 | -.52 |  |
| Based on estimated marginal means | | | | | | |
| ***. The mean difference is significant at the < .001 level. | | | | | | |
| b. Adjustment for multiple comparisons: Bonferroni. | | | | | | |

**Table B. Multiple Comparisons of Attribution Difference Scores in Study 1.**

| Valence | (I) Domain | (J) Domain | Mean Difference (I-J) | Std. Error | 95% Confidence Interval for Difference^b^ | |  |
| --- | --- | --- | --- | --- | --- | --- | --- |
|  |  |  |  |  | Lower Bound | Upper Bound |  |
| Positive | Family | Reciprocity | .04 | .21 | -.61 | .70 |  |
|  |  | Bravery | -.28 | .15 | -.76 | .20 |  |
|  |  | Hierarchy | .42 | .18 | -.15 | .98 |  |
|  |  | Equality | **-1.28^***^** | .18 | -1.84 | -.72 |  |
|  |  | Property | **-.62^***^** | .16 | -1.11 | -.12 |  |
|  |  | Unity & Communal Sharing | .10 | .19 | -.50 | .70 |  |
|  | Reciprocity | Family | -.04 | .21 | -.70 | .61 |  |
|  |  | Bravery | -.32 | .22 | -1.03 | .38 |  |
|  |  | Hierarchy | .37 | .21 | -.29 | 1.04 |  |
|  |  | Equality | **-1.32^***^** | .24 | -2.09 | -.55 |  |
|  |  | Property | -.66 | .24 | -1.41 | .09 |  |
|  |  | Unity & Communal Sharing | .06 | .22 | -.64 | .76 |  |
|  | Bravery | Family | .28 | .15 | -.20 | .76 |  |
|  |  | Reciprocity | .32 | .22 | -.38 | 1.03 |  |
|  |  | Hierarchy | **.69^*^** | .19 | .09 | 1.30 |  |
|  |  | Equality | **-1.00^***^** | .19 | -1.60 | -.40 |  |
|  |  | Property | -.34 | .17 | -.88 | .20 |  |
|  |  | Unity & Communal Sharing | .38 | .15 | -.11 | .87 |  |
|  | Hierarchy | Family | -.42 | .18 | -.98 | .15 |  |
|  |  | Reciprocity | -.37 | .21 | -1.04 | .29 |  |
|  |  | Bravery | **-.69^*^** | .19 | -1.30 | -.09 |  |
|  |  | Equality | **-1.69^***^** | .23 | -2.43 | -.96 |  |
|  |  | Property | **-1.03^***^** | .22 | -1.73 | -.34 |  |
|  |  | Unity & Communal Sharing | -.31 | .20 | -.95 | .32 |  |
|  | Equality | Family | **1.28^***^** | .18 | .72 | 1.84 |  |
|  |  | Reciprocity | **1.32^***^** | .24 | .55 | 2.09 |  |
|  |  | Bravery | **1.00^***^** | .19 | .40 | 1.60 |  |
|  |  | Hierarchy | **1.69^***^** | .23 | .96 | 2.43 |  |
|  |  | Property | **.66^***^** | .15 | .18 | 1.14 |  |
|  |  | Unity & Communal Sharing | **1.38^***^** | .19 | .78 | 1.98 |  |
|  | Property | Family | **.62^***^** | .16 | .12 | 1.11 |  |
|  |  | Reciprocity | .66 | .24 | -.09 | 1.41 |  |
|  |  | Bravery | .34 | .17 | -.20 | .88 |  |
|  |  | Hierarchy | **1.03^***^** | .22 | .34 | 1.73 |  |
|  |  | Equality | **-.66^***^** | .15 | -1.14 | -.18 |  |
|  |  | Unity & Communal Sharing | .72^*^ | .19 | .13 | 1.31 |  |
|  | Unity & Communal Sharing | Family | -.10 | .19 | -.70 | .50 |  |
|  |  | Reciprocity | -.06 | .22 | -.76 | .64 |  |
|  |  | Bravery | -.38 | .15 | -.87 | .11 |  |
|  |  | Hierarchy | .31 | .20 | -.32 | .95 |  |
|  |  | Equality | **-1.38^***^** | .19 | -1.98 | -.78 |  |
|  |  | Property | -.72^*^ | .19 | -1.31 | -.13 |  |
| Negative | Family | Reciprocity | -.38 | .19 | -.98 | .22 |  |
|  |  | Bravery | .12 | .19 | -.49 | .72 |  |
|  |  | Hierarchy | -.25 | .20 | -.89 | .40 |  |
|  |  | Equality | **-1.36^***^** | .20 | -1.98 | -.73 |  |
|  |  | Property | **-1.09^***^** | .22 | -1.80 | -.39 |  |
|  |  | Unity & Communal Sharing | -.12 | .19 | -.74 | .50 |  |
|  | Reciprocity | Family | .38 | .19 | -.22 | .98 |  |
|  |  | Bravery | .50 | .20 | -.13 | 1.13 |  |
|  |  | Hierarchy | .14 | .17 | -.40 | .67 |  |
|  |  | Equality | **-.97^***^** | .24 | -1.75 | -.20 |  |
|  |  | Property | **-.71^*^** | .19 | -1.30 | -.12 |  |
|  |  | Unity & Communal Sharing | .26 | .21 | -.42 | .94 |  |
|  | Bravery | Family | -.12 | .19 | -.72 | .49 |  |
|  |  | Reciprocity | -.50 | .20 | -1.13 | .13 |  |
|  |  | Hierarchy | -.36 | .18 | -.94 | .21 |  |
|  |  | Equality | **-1.47^***^** | .21 | -2.15 | -.79 |  |
|  |  | Property | **-1.21^***^** | .22 | -1.92 | -.51 |  |
|  |  | Unity & Communal Sharing | -.24 | .21 | -.89 | .42 |  |
|  | Hierarchy | Family | .25 | .20 | -.40 | .89 |  |
|  |  | Reciprocity | -.14 | .17 | -.67 | .40 |  |
|  |  | Bravery | .36 | .18 | -.21 | .94 |  |
|  |  | Equality | **-1.11^***^** | .25 | -1.90 | -.32 |  |
|  |  | Property | **-.85^***^** | .21 | -1.53 | -.17 |  |
|  |  | Unity & Communal Sharing | .13 | .20 | -.51 | .76 |  |
|  | Equality | Family | **1.36^***^** | .20 | .73 | 1.98 |  |
|  |  | Reciprocity | **.97^***^** | .24 | .20 | 1.75 |  |
|  |  | Bravery | **1.47^***^** | .21 | .79 | 2.15 |  |
|  |  | Hierarchy | **1.11^***^** | .25 | .32 | 1.90 |  |
|  |  | Property | .26 | .22 | -.44 | .96 |  |
|  |  | Unity & Communal Sharing | **1.24^***^** | .23 | .51 | 1.96 |  |
|  | Property | Family | **1.09^***^** | .22 | .39 | 1.80 |  |
|  |  | Reciprocity | **.71^*^** | .19 | .12 | 1.30 |  |
|  |  | Bravery | **1.21^***^** | .22 | .51 | 1.92 |  |
|  |  | Hierarchy | **.85^***^** | .21 | .17 | 1.53 |  |
|  |  | Equality | -.26 | .22 | -.96 | .44 |  |
|  |  | Unity & Communal Sharing | **.97^***^** | .19 | .37 | 1.58 |  |
|  | Unity & Communal Sharing | Family | .12 | .19 | -.50 | .74 |  |
|  |  | Reciprocity | -.26 | .21 | -.94 | .42 |  |
|  |  | Bravery | .24 | .21 | -.42 | .89 |  |
|  |  | Hierarchy | -.13 | .20 | -.76 | .51 |  |
|  |  | Equality | **-1.24^***^** | .23 | -1.96 | -.51 |  |
|  |  | Property | **-.97^***^** | .19 | -1.58 | -.37 |  |
| Based on estimated marginal means | | | | | | | |
| *. The mean difference is significant at the .05 level. ***. The mean difference is significant at the .001 level. | | | | | | | |
| b. Adjustment for multiple comparisons: Bonferroni. | | | | | | | |
