# Supplementary material for: Separate and unequal: Moral domains differ in corresponding social judgments of others
Source: PLoS One. 2026 Jan 8;21(1):e0338026. doi: 10.1371/journal.pone.0338026 (PMC12782401; doi:10.1371/journal.pone.0338026)
Supplement: S3 Appendix — (DOCX) [file pone.0338026.s003.docx]

**S3 Appendix. Study 2 Analyses Not Using Difference Scores.**

**Fig A. Correspondent Inferences by Domain and Valence in Study 2 (No Difference Scores).**

**
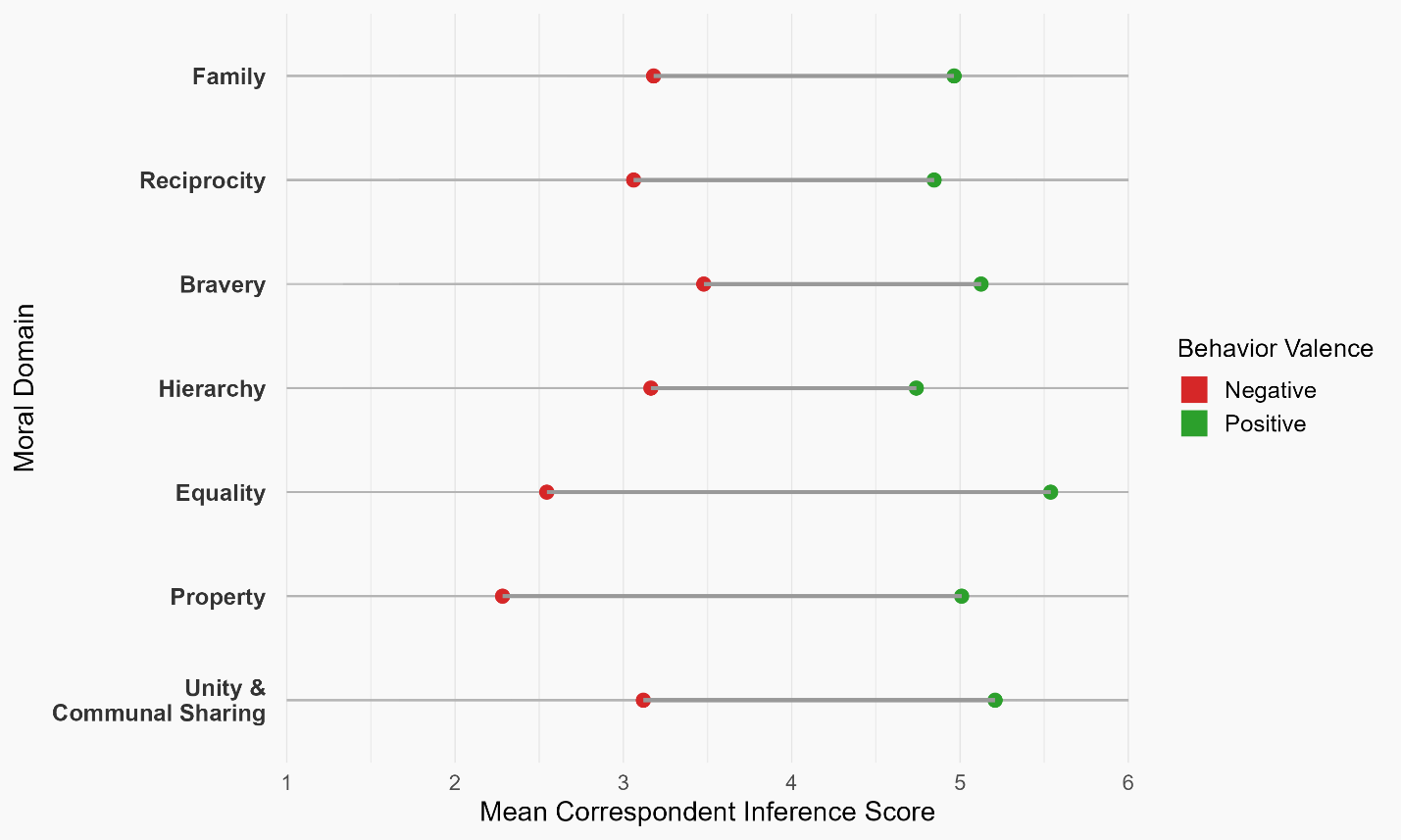
**

Dumbbell plot shows individual and group-level mean ratings of how principled, ethical, and morally upstanding participants found social targets following positive and negative behaviors in each domain. As expected, positive behaviors were met with higher character ratings (*M* = 5.06, *SD* = .83) than negative behaviors (*M* = 2.98, *SD* = 1.03), *F*(1, 103) = 640.08, *η^2^_p_* = .86, *p* < .001. There was also an interaction between valence and domain, *F*(5.57, 573.93) = 20.90, *η^2^_p_* = .17, *p* < .001. Once again there were larger differences in inferences following positive and negative behaviors in the *Equality* and *Property* domains. Bonferroni-adjusted multiple comparisons of ratings across domains by behavior valence are shown in Table A below.

**Table A. Multiple Comparisons of Correspondent Inferences in Study 2 (No Difference Scores).**

| Valence | (I) Domain | (J) Domain | Mean Difference (I-J) | Std. Error | 95% Confidence Interval for Difference^b^ | |  |
| --- | --- | --- | --- | --- | --- | --- | --- |
|  |  |  |  |  | Lower Bound | Upper Bound |  |
| Positive | Family | Reciprocity | .12 | .10 | -.19 | .43 |  |
|  |  | Bravery | -.16 | .08 | -.42 | .10 |  |
|  |  | Hierarchy | .22 | .10 | -.09 | .54 |  |
|  |  | Equality | **-.57^***^** | .07 | -.79 | -.36 |  |
|  |  | Property | -.04 | .09 | -.33 | .24 |  |
|  |  | Unity & Communal Sharing | -.24 | .09 | -.53 | .04 |  |
|  | Reciprocity | Family | -.12 | .10 | -.43 | .19 |  |
|  |  | Bravery | -.28 | .09 | -.57 | .01 |  |
|  |  | Hierarchy | .11 | .11 | -.24 | .45 |  |
|  |  | Equality | **-.69^***^** | .10 | -1.01 | -.38 |  |
|  |  | Property | -.16 | .11 | -.50 | .17 |  |
|  |  | Unity & Communal Sharing | -.36 | .12 | -.73 | .01 |  |
|  | Bravery | Family | .16 | .08 | -.10 | .42 |  |
|  |  | Reciprocity | .28 | .09 | -.01 | .57 |  |
|  |  | Hierarchy | **.38^*^** | .10 | .07 | .70 |  |
|  |  | Equality | **-.41^***^** | .08 | -.67 | -.16 |  |
|  |  | Property | .12 | .08 | -.14 | .37 |  |
|  |  | Unity & Communal Sharing | -.08 | .09 | -.36 | .20 |  |
|  | Hierarchy | Family | -.22 | .10 | -.54 | .09 |  |
|  |  | Reciprocity | -.11 | .11 | -.45 | .24 |  |
|  |  | Bravery | **-.38^*^** | .10 | -.70 | -.07 |  |
|  |  | Equality | **-.80^***^** | .10 | -1.12 | -.48 |  |
|  |  | Property | -.27 | .11 | -.61 | .07 |  |
|  |  | Unity & Communal Sharing | **-.47^***^** | .10 | -.78 | -.16 |  |
|  | Equality | Family | **.57^***^** | .07 | .36 | .79 |  |
|  |  | Reciprocity | **.69^***^** | .10 | .38 | 1.01 |  |
|  |  | Bravery | **.41^***^** | .08 | .16 | .67 |  |
|  |  | Hierarchy | **.80^***^** | .10 | .48 | 1.12 |  |
|  |  | Property | **.53^***^** | .09 | .24 | .82 |  |
|  |  | Unity & Communal Sharing | **.33^***^** | .08 | .08 | .58 |  |
|  | Property | Family | .04 | .09 | -.24 | .33 |  |
|  |  | Reciprocity | .16 | .11 | -.17 | .50 |  |
|  |  | Bravery | -.12 | .08 | -.37 | .14 |  |
|  |  | Hierarchy | .27 | .11 | -.07 | .61 |  |
|  |  | Equality | **-.53^***^** | .09 | -.82 | -.24 |  |
|  |  | Unity & Communal Sharing | -.20 | .10 | -.50 | .10 |  |
|  | Unity & Communal Sharing | Family | .24 | .09 | -.04 | .53 |  |
|  |  | Reciprocity | .36 | .12 | -.01 | .73 |  |
|  |  | Bravery | .08 | .09 | -.20 | .36 |  |
|  |  | Hierarchy | **.47^***^** | .10 | .16 | .78 |  |
|  |  | Equality | **-.33^***^** | .08 | -.58 | -.08 |  |
|  |  | Property | .20 | .10 | -.10 | .50 |  |
| Negative | Family | Reciprocity | .12 | .11 | -.22 | .45 |  |
|  |  | Bravery | -.30 | .11 | -.63 | .03 |  |
|  |  | Hierarchy | .02 | .13 | -.38 | .41 |  |
|  |  | Equality | **.63^***^** | .14 | .19 | 1.08 |  |
|  |  | Property | **.90^***^** | .12 | .51 | 1.29 |  |
|  |  | Unity & Communal Sharing | .06 | .13 | -.34 | .46 |  |
|  | Reciprocity | Family | -.12 | .11 | -.45 | .22 |  |
|  |  | Bravery | **-.42^*^** | .11 | -.76 | -.07 |  |
|  |  | Hierarchy | -.10 | .13 | -.49 | .29 |  |
|  |  | Equality | **.52^*^** | .14 | .07 | .96 |  |
|  |  | Property | **.78^***^** | .12 | .40 | 1.15 |  |
|  |  | Unity & Communal Sharing | -.06 | .14 | -.49 | .37 |  |
|  | Bravery | Family | .30 | .11 | -.03 | .63 |  |
|  |  | Reciprocity | **.42^*^** | .11 | .07 | .76 |  |
|  |  | Hierarchy | .31 | .14 | -.11 | .73 |  |
|  |  | Equality | **.93^***^** | .14 | .48 | 1.38 |  |
|  |  | Property | **1.20^***^** | .13 | .79 | 1.60 |  |
|  |  | Unity & Communal Sharing | .36 | .13 | -.04 | .76 |  |
|  | Hierarchy | Family | -.02 | .13 | -.41 | .38 |  |
|  |  | Reciprocity | .10 | .13 | -.29 | .49 |  |
|  |  | Bravery | -.31 | .14 | -.73 | .11 |  |
|  |  | Equality | **.62^***^** | .13 | .20 | 1.03 |  |
|  |  | Property | **.88^***^** | .10 | .57 | 1.19 |  |
|  |  | Unity & Communal Sharing | .04 | .12 | -.31 | .40 |  |
|  | Equality | Family | **-.63^***^** | .14 | -1.08 | -.19 |  |
|  |  | Reciprocity | **-.52^*^** | .14 | -.96 | -.07 |  |
|  |  | Bravery | **-.93^***^** | .14 | -1.38 | -.48 |  |
|  |  | Hierarchy | **-.62^***^** | .13 | -1.03 | -.20 |  |
|  |  | Property | .26 | .13 | -.14 | .67 |  |
|  |  | Unity & Communal Sharing | **-.57^***^** | .14 | -1.00 | -.14 |  |
|  | Property | Family | **-.90^***^** | .12 | -1.29 | -.51 |  |
|  |  | Reciprocity | **-.78^***^** | .12 | -1.15 | -.40 |  |
|  |  | Bravery | **-1.20^***^** | .13 | -1.60 | -.79 |  |
|  |  | Hierarchy | **-.88^***^** | .10 | -1.19 | -.57 |  |
|  |  | Equality | -.26 | .13 | -.67 | .14 |  |
|  |  | Unity & Communal Sharing | **-.84^***^** | .11 | -1.18 | -.50 |  |
|  | Unity & Communal Sharing | Family | -.06 | .13 | -.46 | .34 |  |
|  |  | Reciprocity | .06 | .14 | -.37 | .49 |  |
|  |  | Bravery | -.36 | .13 | -.76 | .04 |  |
|  |  | Hierarchy | -.04 | .12 | -.40 | .31 |  |
|  |  | Equality | **.57^***^** | .14 | .14 | 1.00 |  |
|  |  | Property | **.84^***^** | .11 | .50 | 1.18 |  |
| Based on estimated marginal means | | | | | | | |
| *. The mean difference is significant at the .05 level. ***. The mean difference is significant at the .001 level. | | | | | | | |
| b. Adjustment for multiple comparisons: Bonferroni. | | | | | | | |

**Fig B. Dispositional and Situational Attributions by Domain and Valence in Study 2 (No Difference Scores).**

*
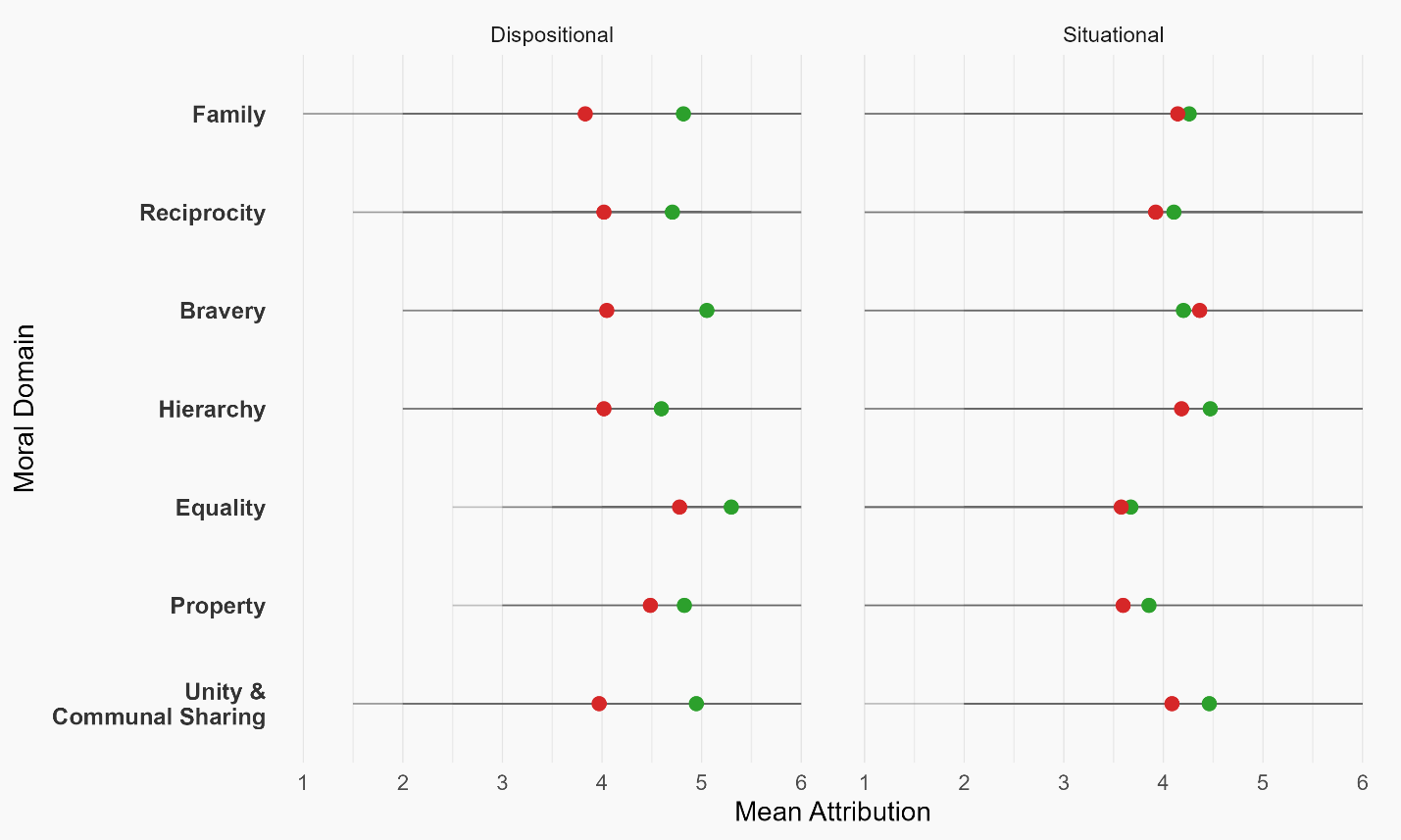
*

Dumbbell plot shows individual and group-level mean ratings of dispositional attribution and situational attribution following positive and negative behaviors in each domain. Behaviors related to *Equality* and *Property* were more dispositionally attributed than most other domains and less situationally attributed than all other domains, *F_dispositional_* (5.62, 578.91) = 30.84, *η^2^_p_* = .23, *p* < .001, *F_situational_* (5.70, 587.40) = 19.72, *η^2^_p_* = .16, *p* < .001. There were also interaction effects between domain and valence for both types of attribution, showing that the effect of behavior valence on attribution statistically differed across domains, *F_dispositional_* (5.46, 562.82) = 10.04, *η^2^_p_* = .09, *p* < .001, *F_situational_* (5.55, 572.09) = 2.15, *η^2^_p_* = .02, *p* = .05. For comparisons of attribution by valence and domain see Tables B and C below.

**Table B. Multiple Comparisons of Dispositional Attribution in Study 2 (No Difference Scores).**

| Valence | (I) Domain | (J) Domain | Mean Difference (I-J) | Std. Error | 95% Confidence Interval for Difference^b^ | |  |
| --- | --- | --- | --- | --- | --- | --- | --- |
|  |  |  |  |  | Lower Bound | Upper Bound |  |
| Positive | Family | Reciprocity | .11 | .08 | -.13 | .35 |  |
|  |  | Bravery | **-.24^*^** | .07 | -.45 | -.02 |  |
|  |  | Hierarchy | **.22^*^** | .07 | .01 | .43 |  |
|  |  | Equality | **-.48^***^** | .06 | -.68 | -.28 |  |
|  |  | Property | -.01 | .06 | -.20 | .18 |  |
|  |  | Unity & Communal Sharing | -.13 | .06 | -.33 | .07 |  |
|  | Reciprocity | Family | -.11 | .08 | -.35 | .13 |  |
|  |  | Bravery | **-.35^***^** | .07 | -.56 | -.14 |  |
|  |  | Hierarchy | .11 | .10 | -.20 | .42 |  |
|  |  | Equality | **-.59^***^** | .08 | -.83 | -.35 |  |
|  |  | Property | -.12 | .08 | -.36 | .12 |  |
|  |  | Unity & Communal Sharing | -.24 | .09 | -.51 | .02 |  |
|  | Bravery | Family | **.24^*^** | .07 | .02 | .45 |  |
|  |  | Reciprocity | **.35^***^** | .07 | .14 | .56 |  |
|  |  | Hierarchy | **.46^***^** | .07 | .23 | .69 |  |
|  |  | Equality | **-.25^***^** | .06 | -.44 | -.05 |  |
|  |  | Property | **.23^***^** | .06 | .05 | .41 |  |
|  |  | Unity & Communal Sharing | .11 | .06 | -.09 | .30 |  |
|  | Hierarchy | Family | **-.22^*^** | .07 | -.43 | -.01 |  |
|  |  | Reciprocity | -.11 | .10 | -.42 | .20 |  |
|  |  | Bravery | **-.46^***^** | .07 | -.69 | -.23 |  |
|  |  | Equality | **-.70^***^** | .09 | -.97 | -.44 |  |
|  |  | Property | -.23 | .08 | -.49 | .02 |  |
|  |  | Unity & Communal Sharing | **-.35^***^** | .08 | -.61 | -.09 |  |
|  | Equality | Family | **.48^***^** | .06 | .28 | .68 |  |
|  |  | Reciprocity | **.59^***^** | .08 | .35 | .83 |  |
|  |  | Bravery | **.25^***^** | .06 | .05 | .44 |  |
|  |  | Hierarchy | **.70^***^** | .09 | .44 | .97 |  |
|  |  | Property | **.47^***^** | .07 | .26 | .68 |  |
|  |  | Unity & Communal Sharing | **.35^***^** | .06 | .17 | .54 |  |
|  | Property | Family | .01 | .06 | -.18 | .20 |  |
|  |  | Reciprocity | .12 | .08 | -.12 | .36 |  |
|  |  | Bravery | **-.23^***^** | .06 | -.41 | -.05 |  |
|  |  | Hierarchy | .23 | .08 | -.02 | .49 |  |
|  |  | Equality | **-.47^***^** | .07 | -.68 | -.26 |  |
|  |  | Unity & Communal Sharing | -.12 | .06 | -.32 | .08 |  |
|  | Unity & Communal Sharing | Family | .13 | .06 | -.07 | .33 |  |
|  |  | Reciprocity | .24 | .09 | -.02 | .51 |  |
|  |  | Bravery | -.11 | .06 | -.30 | .09 |  |
|  |  | Hierarchy | **.35^***^** | .08 | .09 | .61 |  |
|  |  | Equality | **-.35^***^** | .06 | -.54 | -.17 |  |
|  |  | Property | .12 | .06 | -.08 | .32 |  |
| Negative | Family | Reciprocity | -.19 | .09 | -.48 | .10 |  |
|  |  | Bravery | -.22 | .12 | -.58 | .14 |  |
|  |  | Hierarchy | -.19 | .11 | -.53 | .15 |  |
|  |  | Equality | **-.95^***^** | .11 | -1.30 | -.60 |  |
|  |  | Property | **-.65^***^** | .11 | -1.01 | -.30 |  |
|  |  | Unity & Communal Sharing | -.14 | .12 | -.50 | .22 |  |
|  | Reciprocity | Family | .19 | .09 | -.10 | .48 |  |
|  |  | Bravery | -.03 | .10 | -.35 | .29 |  |
|  |  | Hierarchy | .00 | .11 | -.34 | .34 |  |
|  |  | Equality | **-.76^***^** | .10 | -1.08 | -.44 |  |
|  |  | Property | **-.47^***^** | .09 | -.75 | -.18 |  |
|  |  | Unity & Communal Sharing | .05 | .10 | -.27 | .36 |  |
|  | Bravery | Family | .22 | .12 | -.14 | .58 |  |
|  |  | Reciprocity | .03 | .10 | -.29 | .35 |  |
|  |  | Hierarchy | .03 | .10 | -.28 | .34 |  |
|  |  | Equality | **-.73^***^** | .10 | -1.06 | -.41 |  |
|  |  | Property | **-.44^***^** | .09 | -.72 | -.15 |  |
|  |  | Unity & Communal Sharing | .08 | .10 | -.25 | .40 |  |
|  | Hierarchy | Family | .19 | .11 | -.15 | .53 |  |
|  |  | Reciprocity | .00 | .11 | -.34 | .34 |  |
|  |  | Bravery | -.03 | .10 | -.34 | .28 |  |
|  |  | Equality | **-.76^***^** | .10 | -1.07 | -.45 |  |
|  |  | Property | **-.47^***^** | .09 | -.73 | -.20 |  |
|  |  | Unity & Communal Sharing | .05 | .09 | -.24 | .34 |  |
|  | Equality | Family | **.95^***^** | .11 | .60 | 1.30 |  |
|  |  | Reciprocity | **.76^***^** | .10 | .44 | 1.08 |  |
|  |  | Bravery | **.73^***^** | .10 | .41 | 1.06 |  |
|  |  | Hierarchy | **.76^***^** | .10 | .45 | 1.07 |  |
|  |  | Property | **.29^*^** | .09 | .01 | .57 |  |
|  |  | Unity & Communal Sharing | **.81^***^** | .11 | .48 | 1.14 |  |
|  | Property | Family | **.65^***^** | .11 | .30 | 1.01 |  |
|  |  | Reciprocity | **.47^***^** | .09 | .18 | .75 |  |
|  |  | Bravery | **.44^***^** | .09 | .15 | .72 |  |
|  |  | Hierarchy | **.47^***^** | .09 | .20 | .73 |  |
|  |  | Equality | **-.29^*^** | .09 | -.57 | -.01 |  |
|  |  | Unity & Communal Sharing | **.51^***^** | .09 | .24 | .79 |  |
|  | Unity & Communal Sharing | Family | .14 | .12 | -.22 | .50 |  |
|  |  | Reciprocity | -.05 | .10 | -.36 | .27 |  |
|  |  | Bravery | -.08 | .10 | -.40 | .25 |  |
|  |  | Hierarchy | -.05 | .09 | -.34 | .24 |  |
|  |  | Equality | **-.81^***^** | .11 | -1.14 | -.48 |  |
|  |  | Property | **-.51^***^** | .09 | -.79 | -.24 |  |
| Based on estimated marginal means | | | | | | | |
| *. The mean difference is significant at the .05 level. ***. The mean difference is significant at the .001 level. | | | | | | | |
| b. Adjustment for multiple comparisons: Bonferroni. | | | | | | | |

**Table C. Multiple Comparisons of Situational Attribution in Study 2 (No Difference Scores).**

| Valence | (I) Domain | (J) Domain | Mean Difference (I-J) | Std. Error | 95% Confidence Interval for Difference^b^ | |  |
| --- | --- | --- | --- | --- | --- | --- | --- |
|  |  |  |  |  | Lower Bound | Upper Bound |  |
| Positive | Family | Reciprocity | .15 | .11 | -.19 | .50 |  |
|  |  | Bravery | .06 | .11 | -.29 | .41 |  |
|  |  | Hierarchy | -.21 | .12 | -.59 | .17 |  |
|  |  | Equality | **.59^***^** | .11 | .26 | .92 |  |
|  |  | Property | **.40^*^** | .11 | .06 | .75 |  |
|  |  | Unity & Communal Sharing | -.20 | .10 | -.51 | .11 |  |
|  | Reciprocity | Family | -.15 | .11 | -.50 | .19 |  |
|  |  | Bravery | -.10 | .12 | -.48 | .28 |  |
|  |  | Hierarchy | -.37 | .13 | -.78 | .05 |  |
|  |  | Equality | **.43^*^** | .14 | .01 | .86 |  |
|  |  | Property | .25 | .13 | -.15 | .65 |  |
|  |  | Unity & Communal Sharing | -.36 | .12 | -.72 | .01 |  |
|  | Bravery | Family | -.06 | .11 | -.41 | .29 |  |
|  |  | Reciprocity | .10 | .12 | -.28 | .48 |  |
|  |  | Hierarchy | -.27 | .14 | -.70 | .16 |  |
|  |  | Equality | **.53^***^** | .13 | .13 | .93 |  |
|  |  | Property | .35 | .14 | -.07 | .77 |  |
|  |  | Unity & Communal Sharing | -.26 | .12 | -.64 | .12 |  |
|  | Hierarchy | Family | .21 | .12 | -.17 | .59 |  |
|  |  | Reciprocity | .37 | .13 | -.05 | .78 |  |
|  |  | Bravery | .27 | .14 | -.16 | .70 |  |
|  |  | Equality | **.80^***^** | .15 | .34 | 1.26 |  |
|  |  | Property | **.62^***^** | .14 | .17 | 1.06 |  |
|  |  | Unity & Communal Sharing | .01 | .11 | -.34 | .36 |  |
|  | Equality | Family | **-.59^***^** | .11 | -.92 | -.26 |  |
|  |  | Reciprocity | **-.43^*^** | .14 | -.86 | -.01 |  |
|  |  | Bravery | **-.53^***^** | .13 | -.93 | -.13 |  |
|  |  | Hierarchy | **-.80^***^** | .15 | -1.26 | -.34 |  |
|  |  | Property | -.18 | .13 | -.59 | .22 |  |
|  |  | Unity & Communal Sharing | **-.79^***^** | .13 | -1.19 | -.39 |  |
|  | Property | Family | **-.40^*^** | .11 | -.75 | -.06 |  |
|  |  | Reciprocity | -.25 | .13 | -.65 | .15 |  |
|  |  | Bravery | -.35 | .14 | -.77 | .07 |  |
|  |  | Hierarchy | **-.62^***^** | .14 | -1.06 | -.17 |  |
|  |  | Equality | .18 | .13 | -.22 | .59 |  |
|  |  | Unity & Communal Sharing | **-.61^***^** | .13 | -1.00 | -.21 |  |
|  | Unity & Communal Sharing | Family | .20 | .10 | -.11 | .51 |  |
|  |  | Reciprocity | .36 | .12 | -.01 | .72 |  |
|  |  | Bravery | .26 | .12 | -.12 | .64 |  |
|  |  | Hierarchy | -.01 | .11 | -.36 | .34 |  |
|  |  | Equality | **.79^***^** | .13 | .39 | 1.19 |  |
|  |  | Property | **.61^***^** | .13 | .21 | 1.00 |  |
| Negative | Family | Reciprocity | .22 | .09 | -.05 | .49 |  |
|  |  | Bravery | -.22 | .13 | -.61 | .17 |  |
|  |  | Hierarchy | -.04 | .13 | -.44 | .37 |  |
|  |  | Equality | **.57^***^** | .14 | .14 | 1.00 |  |
|  |  | Property | **.55^***^** | .14 | .13 | .97 |  |
|  |  | Unity & Communal Sharing | .06 | .13 | -.35 | .46 |  |
|  | Reciprocity | Family | -.22 | .09 | -.49 | .05 |  |
|  |  | Bravery | **-.44^*^** | .12 | -.81 | -.07 |  |
|  |  | Hierarchy | -.26 | .13 | -.68 | .16 |  |
|  |  | Equality | .35 | .13 | -.04 | .74 |  |
|  |  | Property | .33 | .12 | -.04 | .70 |  |
|  |  | Unity & Communal Sharing | -.16 | .12 | -.54 | .21 |  |
|  | Bravery | Family | .22 | .13 | -.17 | .61 |  |
|  |  | Reciprocity | **.44^*^** | .12 | .07 | .81 |  |
|  |  | Hierarchy | .18 | .12 | -.19 | .55 |  |
|  |  | Equality | **.79^***^** | .13 | .39 | 1.19 |  |
|  |  | Property | **.77^***^** | .12 | .39 | 1.15 |  |
|  |  | Unity & Communal Sharing | .28 | .10 | -.02 | .58 |  |
|  | Hierarchy | Family | .04 | .13 | -.37 | .44 |  |
|  |  | Reciprocity | .26 | .13 | -.16 | .68 |  |
|  |  | Bravery | -.18 | .12 | -.55 | .19 |  |
|  |  | Equality | **.61^***^** | .13 | .20 | 1.01 |  |
|  |  | Property | **.59^***^** | .11 | .23 | .94 |  |
|  |  | Unity & Communal Sharing | .10 | .12 | -.27 | .46 |  |
|  | Equality | Family | **-.57^***^** | .14 | -1.00 | -.14 |  |
|  |  | Reciprocity | -.35 | .13 | -.74 | .04 |  |
|  |  | Bravery | **-.79^***^** | .13 | -1.19 | -.39 |  |
|  |  | Hierarchy | **-.61^***^** | .13 | -1.01 | -.20 |  |
|  |  | Property | -.02 | .13 | -.42 | .38 |  |
|  |  | Unity & Communal Sharing | **-.51^*^** | .14 | -.93 | -.09 |  |
|  | Property | Family | **-.55^***^** | .14 | -.97 | -.13 |  |
|  |  | Reciprocity | -.33 | .12 | -.70 | .04 |  |
|  |  | Bravery | **-.77^***^** | .12 | -1.15 | -.39 |  |
|  |  | Hierarchy | **-.59^***^** | .11 | -.94 | -.23 |  |
|  |  | Equality | .02 | .13 | -.38 | .42 |  |
|  |  | Unity & Communal Sharing | **-.49^***^** | .12 | -.87 | -.11 |  |
|  | Unity & Communal Sharing | Family | -.06 | .13 | -.46 | .35 |  |
|  |  | Reciprocity | .16 | .12 | -.21 | .54 |  |
|  |  | Bravery | -.28 | .10 | -.58 | .02 |  |
|  |  | Hierarchy | -.10 | .12 | -.46 | .27 |  |
|  |  | Equality | **.51^*^** | .14 | .09 | .93 |  |
|  |  | Property | **.49^***^** | .12 | .11 | .87 |  |
| Based on estimated marginal means | | | | | | | |
| *. The mean difference is significant at the .05 level. ***. The mean difference is significant at the .001 level. | | | | | | | |
| b. Adjustment for multiple comparisons: Bonferroni. | | | | | | | |

**Fig C. Willingness to Cooperate by Domain and Valence in Study 2 (No Difference Scores).**

*
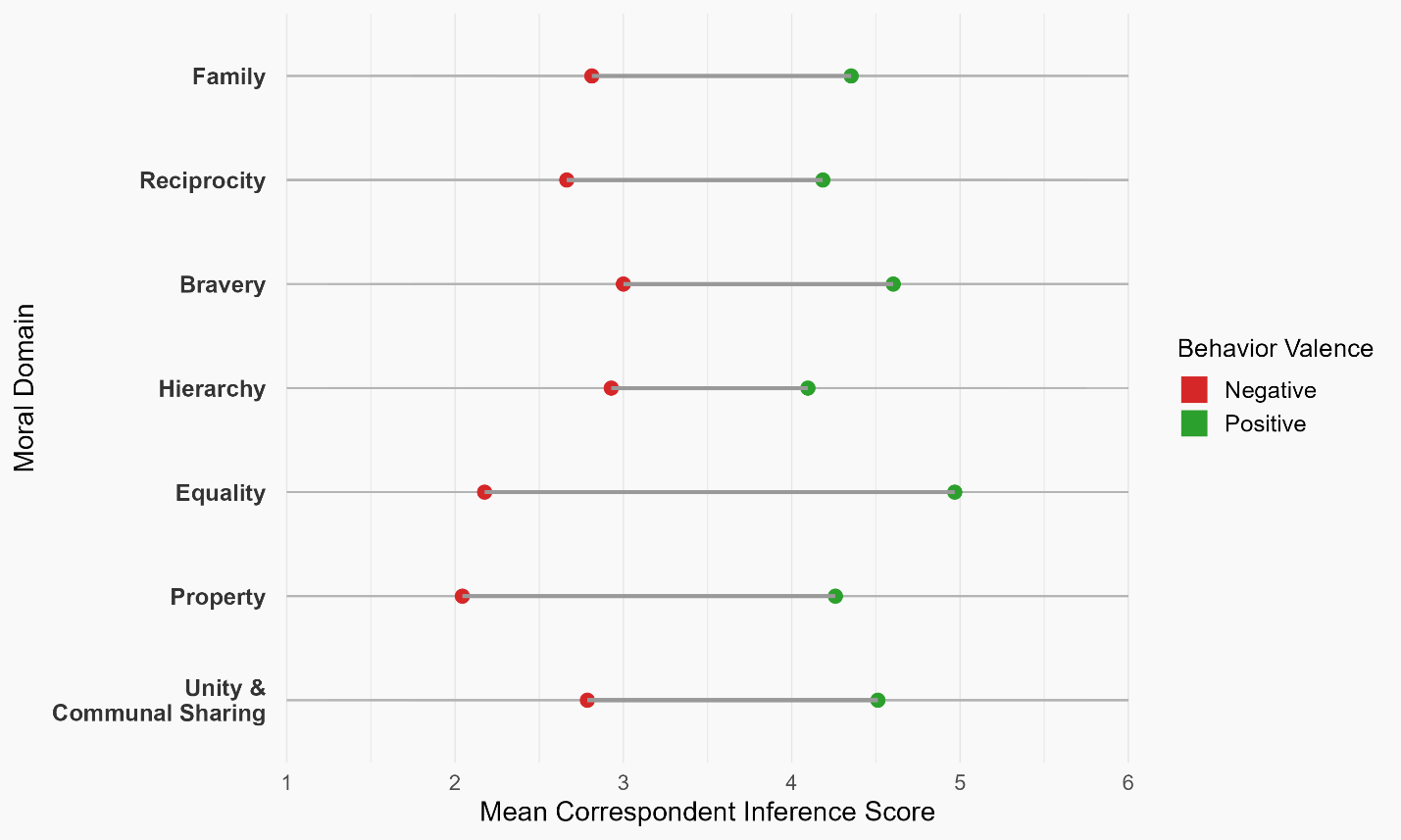
*

Dumbbell plot shows individual and group-level mean ratings of how willing participants would be to do the following with a social target: tell them a personal secret, trust them to solve a dispute, seek advice from them, or share a car/ride with them. In general, participants were more likely to cooperate with targets following positive behaviors (*M* = 4.43, *SD* = .92) than negative behaviors (*M* = 2.63, *SD* = 1.03), *F*(1, 103) = 485.58, *η^2^_p_* = .83, *p* < .001. An interaction between valence and domain, *F*(5.26, 541.55) = 24.64, *η^2^_p_* = .19, *p* < .001, showed that some domains were more sensitive to valence than others. The directions of these effects are shown in the Bonferroni-adjusted multiple comparisons in Table D below.

**Table D. Multiple Comparisons of Willingness to Cooperate with Targets in Study 2 (No Difference Scores).**

| Valence | (I) Domain | (J) Domain | Mean Difference (I-J) | Std. Error | 95% Confidence Interval for Difference^b^ | |  |
| --- | --- | --- | --- | --- | --- | --- | --- |
|  |  |  |  |  | Lower Bound | Upper Bound |  |
| Positive | Family | Reciprocity | .17 | .09 | -.10 | .43 |  |
|  |  | Bravery | -.25 | .08 | -.50 | .00 |  |
|  |  | Hierarchy | .26 | .08 | -.01 | .52 |  |
|  |  | Equality | **-.62^***^** | .07 | -.83 | -.40 |  |
|  |  | Property | .09 | .08 | -.15 | .34 |  |
|  |  | Unity & Communal Sharing | -.16 | .09 | -.44 | .13 |  |
|  | Reciprocity | Family | -.17 | .09 | -.43 | .10 |  |
|  |  | Bravery | **-.42^***^** | .09 | -.70 | -.13 |  |
|  |  | Hierarchy | .09 | .10 | -.23 | .41 |  |
|  |  | Equality | **-.78^***^** | .10 | -1.09 | -.48 |  |
|  |  | Property | -.07 | .09 | -.37 | .22 |  |
|  |  | Unity & Communal Sharing | **-.33^*^** | .10 | -.65 | .00 |  |
|  | Bravery | Family | .25 | .08 | .00 | .50 |  |
|  |  | Reciprocity | **.42^***^** | .09 | .13 | .70 |  |
|  |  | Hierarchy | **.51^***^** | .09 | .23 | .78 |  |
|  |  | Equality | **-.37^***^** | .08 | -.62 | -.11 |  |
|  |  | Property | **.34^***^** | .07 | .11 | .58 |  |
|  |  | Unity & Communal Sharing | .09 | .08 | -.17 | .35 |  |
|  | Hierarchy | Family | -.26 | .08 | -.52 | .01 |  |
|  |  | Reciprocity | -.09 | .10 | -.41 | .23 |  |
|  |  | Bravery | **-.51^***^** | .09 | -.78 | -.23 |  |
|  |  | Equality | **-.87^***^** | .08 | -1.14 | -.61 |  |
|  |  | Property | -.16 | .09 | -.44 | .12 |  |
|  |  | Unity & Communal Sharing | **-.42^***^** | .09 | -.68 | -.15 |  |
|  | Equality | Family | **.62^***^** | .07 | .40 | .83 |  |
|  |  | Reciprocity | **.78^***^** | .10 | .48 | 1.09 |  |
|  |  | Bravery | **.37^***^** | .08 | .11 | .62 |  |
|  |  | Hierarchy | **.87^***^** | .08 | .61 | 1.14 |  |
|  |  | Property | **.71^***^** | .09 | .44 | .98 |  |
|  |  | Unity & Communal Sharing | **.46^***^** | .08 | .19 | .72 |  |
|  | Property | Family | -.09 | .08 | -.34 | .15 |  |
|  |  | Reciprocity | .07 | .09 | -.22 | .37 |  |
|  |  | Bravery | **-.34^***^** | .07 | -.58 | -.11 |  |
|  |  | Hierarchy | .16 | .09 | -.12 | .44 |  |
|  |  | Equality | **-.71^***^** | .09 | -.98 | -.44 |  |
|  |  | Unity & Communal Sharing | -.25 | .09 | -.52 | .01 |  |
|  | Unity & Communal Sharing | Family | .16 | .09 | -.13 | .44 |  |
|  |  | Reciprocity | **.33^*^** | .10 | .00 | .65 |  |
|  |  | Bravery | -.09 | .08 | -.35 | .17 |  |
|  |  | Hierarchy | **.42^***^** | .09 | .15 | .68 |  |
|  |  | Equality | **-.46^***^** | .08 | -.72 | -.19 |  |
|  |  | Property | .25 | .09 | -.01 | .52 |  |
| Negative | Family | Reciprocity | .15 | .08 | -.11 | .40 |  |
|  |  | Bravery | -.19 | .09 | -.47 | .10 |  |
|  |  | Hierarchy | -.12 | .11 | -.47 | .24 |  |
|  |  | Equality | **.64^***^** | .13 | .24 | 1.04 |  |
|  |  | Property | **.77^***^** | .10 | .45 | 1.09 |  |
|  |  | Unity & Communal Sharing | .03 | .12 | -.34 | .40 |  |
|  | Reciprocity | Family | -.15 | .08 | -.40 | .11 |  |
|  |  | Bravery | **-.34^*^** | .10 | -.64 | -.03 |  |
|  |  | Hierarchy | -.26 | .12 | -.65 | .12 |  |
|  |  | Equality | **.49^*^** | .14 | .07 | .91 |  |
|  |  | Property | **.62^***^** | .10 | .29 | .95 |  |
|  |  | Unity & Communal Sharing | -.12 | .12 | -.50 | .26 |  |
|  | Bravery | Family | .19 | .09 | -.10 | .47 |  |
|  |  | Reciprocity | **.34^*^** | .10 | .03 | .64 |  |
|  |  | Hierarchy | .07 | .13 | -.32 | .46 |  |
|  |  | Equality | **.82^***^** | .12 | .44 | 1.21 |  |
|  |  | Property | **.96^***^** | .10 | .64 | 1.28 |  |
|  |  | Unity & Communal Sharing | .21 | .12 | -.15 | .57 |  |
|  | Hierarchy | Family | .12 | .11 | -.24 | .47 |  |
|  |  | Reciprocity | .26 | .12 | -.12 | .65 |  |
|  |  | Bravery | -.07 | .13 | -.46 | .32 |  |
|  |  | Equality | **.75^***^** | .13 | .36 | 1.14 |  |
|  |  | Property | **.88^***^** | .09 | .60 | 1.17 |  |
|  |  | Unity & Communal Sharing | .14 | .10 | -.17 | .45 |  |
|  | Equality | Family | **-.64^***^** | .13 | -1.04 | -.24 |  |
|  |  | Reciprocity | **-.49^*^** | .14 | -.91 | -.07 |  |
|  |  | Bravery | **-.82^***^** | .12 | -1.21 | -.44 |  |
|  |  | Hierarchy | **-.75^***^** | .13 | -1.14 | -.36 |  |
|  |  | Property | .13 | .12 | -.23 | .49 |  |
|  |  | Unity & Communal Sharing | **-.61^***^** | .13 | -1.01 | -.21 |  |
|  | Property | Family | **-.77^***^** | .10 | -1.09 | -.45 |  |
|  |  | Reciprocity | **-.62^***^** | .10 | -.95 | -.29 |  |
|  |  | Bravery | **-.96^***^** | .10 | -1.28 | -.64 |  |
|  |  | Hierarchy | **-.88^***^** | .09 | -1.17 | -.60 |  |
|  |  | Equality | -.13 | .12 | -.49 | .23 |  |
|  |  | Unity & Communal Sharing | **-.74^***^** | .09 | -1.01 | -.48 |  |
|  | Unity & Communal Sharing | Family | -.03 | .12 | -.40 | .34 |  |
|  |  | Reciprocity | .12 | .12 | -.26 | .50 |  |
|  |  | Bravery | -.21 | .12 | -.57 | .15 |  |
|  |  | Hierarchy | -.14 | .10 | -.45 | .17 |  |
|  |  | Equality | **.61^***^** | .13 | .21 | 1.01 |  |
|  |  | Property | **.74^***^** | .09 | .48 | 1.01 |  |
| Based on estimated marginal means | | | | | | | |
| *. The mean difference is significant at the .05 level. ***. The mean difference is significant at the < .001 level. | | | | | | | |
| b. Adjustment for multiple comparisons: Bonferroni. | | | | | | | |
