# Supplementary material for: Separate and unequal: Moral domains differ in corresponding social judgments of others
Source: PLoS One. 2026 Jan 8;21(1):e0338026. doi: 10.1371/journal.pone.0338026 (PMC12782401; doi:10.1371/journal.pone.0338026)
Supplement: S4 Appendix — (DOCX) [file pone.0338026.s004.docx]

**S4 Appendix. Multiple Comparisons for Reported Analyses in Study 2.**

**Table A. Multiple Comparisons of Study 2 Correspondent Inference Difference Scores.**

| (I) Domain | (J) Domain | Mean Difference (I-J) | Std. Error | 95% Confidence Interval for Difference^b^ | |  |
| --- | --- | --- | --- | --- | --- | --- |
|  |  |  |  | Lower Bound | Upper Bound |  |
| Family | Reciprocity | 4.44E-16 | .16 | -.49 | .49 |  |
|  | Bravery | .14 | .14 | -.29 | .57 |  |
|  | Hierarchy | .21 | .19 | -.38 | .80 |  |
|  | Equality | **-1.21^***^** | .17 | -1.73 | -.69 |  |
|  | Property | **-.94^***^** | .17 | -1.47 | -.41 |  |
|  | Unity & Communal Sharing | -.30 | .17 | -.84 | .23 |  |
| Reciprocity | Family | -4.44E-16 | .16 | -.49 | .49 |  |
|  | Bravery | .14 | .14 | -.31 | .59 |  |
|  | Hierarchy | .21 | .19 | -.40 | .81 |  |
|  | Equality | **-1.21^***^** | .18 | -1.76 | -.66 |  |
|  | Property | **-.94^***^** | .18 | -1.49 | -.40 |  |
|  | Unity & Communal Sharing | -.30 | .20 | -.92 | .31 |  |
| Bravery | Family | -.14 | .14 | -.57 | .29 |  |
|  | Reciprocity | -.14 | .14 | -.59 | .31 |  |
|  | Hierarchy | .07 | .19 | -.53 | .67 |  |
|  | Equality | **-1.35^***^** | .16 | -1.86 | -.83 |  |
|  | Property | **-1.08^***^** | .16 | -1.59 | -.57 |  |
|  | Unity & Communal Sharing | -.44 | .17 | -.97 | .09 |  |
| Hierarchy | Family | -.21 | .19 | -.80 | .38 |  |
|  | Reciprocity | -.21 | .19 | -.81 | .40 |  |
|  | Bravery | -.07 | .19 | -.67 | .53 |  |
|  | Equality | **-1.42^***^** | .19 | -2.02 | -.81 |  |
|  | Property | **-1.15^***^** | .16 | -1.66 | -.64 |  |
|  | Unity & Communal Sharing | -.51 | .17 | -1.03 | .01 |  |
| Equality | Family | **1.21^***^** | .17 | .69 | 1.73 |  |
|  | Reciprocity | **1.21^***^** | .18 | .66 | 1.76 |  |
|  | Bravery | **1.35^***^** | .16 | .83 | 1.86 |  |
|  | Hierarchy | **1.42^***^** | .19 | .81 | 2.02 |  |
|  | Property | .27 | .17 | -.28 | .81 |  |
|  | Unity & Communal Sharing | **.90^***^** | .18 | .35 | 1.46 |  |
| Property | Family | **.94^***^** | .17 | .41 | 1.47 |  |
|  | Reciprocity | **.94^***^** | .18 | .40 | 1.49 |  |
|  | Bravery | **1.08^***^** | .16 | .57 | 1.59 |  |
|  | Hierarchy | **1.15^***^** | .16 | .64 | 1.66 |  |
|  | Equality | -.27 | .17 | -.81 | .28 |  |
|  | Unity & Communal Sharing | **.64^***^** | .16 | .13 | 1.15 |  |
| Unity & Communal Sharing | Family | .30 | .17 | -.23 | .84 |  |
|  | Reciprocity | .30 | .20 | -.31 | .92 |  |
|  | Bravery | .44 | .17 | -.09 | .97 |  |
|  | Hierarchy | .51 | .17 | -.01 | 1.03 |  |
|  | Equality | **-.90^***^** | .18 | -1.46 | -.35 |  |
|  | Property | **-.64^***^** | .16 | -1.15 | -.13 |  |
| Based on estimated marginal means | | | | | | |
| ***. The mean difference is significant at the .001 level. | | | | | | |
| b. Adjustment for multiple comparisons: Bonferroni. | | | | | | |

**Table B. Multiple Comparisons of Attribution Difference Scores in Study 2.**

| Valence | (I) Domain | (J) Domain | Mean Difference (I-J) | Std. Error | 95% Confidence Interval for Difference^b^ | |  |
| --- | --- | --- | --- | --- | --- | --- | --- |
|  |  |  |  |  | Lower Bound | Upper Bound |  |
| Positive | Family | Reciprocity | -.04 | .14 | -.49 | .40 |  |
|  |  | Bravery | -.29 | .13 | -.69 | .10 |  |
|  |  | Hierarchy | .43 | .14 | .00 | .87 |  |
|  |  | Equality | **-1.07^***^** | .13 | -1.47 | -.67 |  |
|  |  | Property | **-.41^*^** | .12 | -.80 | -.03 |  |
|  |  | Unity & Communal Sharing | .07 | .11 | -.27 | .42 |  |
|  | Reciprocity | Family | .04 | .14 | -.40 | .49 |  |
|  |  | Bravery | -.25 | .14 | -.70 | .20 |  |
|  |  | Hierarchy | .48 | .18 | -.10 | 1.05 |  |
|  |  | Equality | **-1.02^***^** | .16 | -1.53 | -.52 |  |
|  |  | Property | -.37 | .16 | -.86 | .12 |  |
|  |  | Unity & Communal Sharing | .12 | .14 | -.34 | .57 |  |
|  | Bravery | Family | .29 | .13 | -.10 | .69 |  |
|  |  | Reciprocity | .25 | .14 | -.20 | .70 |  |
|  |  | Hierarchy | **.73^***^** | .17 | .21 | 1.25 |  |
|  |  | Equality | **-.77^***^** | .14 | -1.21 | -.34 |  |
|  |  | Property | -.12 | .14 | -.55 | .31 |  |
|  |  | Unity & Communal Sharing | .37 | .13 | -.05 | .78 |  |
|  | Hierarchy | Family | -.43 | .14 | -.87 | .00 |  |
|  |  | Reciprocity | -.48 | .18 | -1.05 | .10 |  |
|  |  | Bravery | **-.73^***^** | .17 | -1.25 | -.21 |  |
|  |  | Equality | **-1.50^***^** | .19 | -2.08 | -.92 |  |
|  |  | Property | **-.85^***^** | .17 | -1.37 | -.32 |  |
|  |  | Unity & Communal Sharing | -.36 | .15 | -.83 | .10 |  |
|  | Equality | Family | **1.07^***^** | .13 | .67 | 1.47 |  |
|  |  | Reciprocity | **1.02^***^** | .16 | .52 | 1.53 |  |
|  |  | Bravery | **.77^***^** | .14 | .34 | 1.21 |  |
|  |  | Hierarchy | **1.50^***^** | .19 | .92 | 2.08 |  |
|  |  | Property | **.65^***^** | .15 | .20 | 1.11 |  |
|  |  | Unity & Communal Sharing | **1.14^***^** | .14 | .70 | 1.58 |  |
|  | Property | Family | .41^*^ | .12 | .03 | .80 |  |
|  |  | Reciprocity | .37 | .16 | -.12 | .86 |  |
|  |  | Bravery | .12 | .14 | -.31 | .55 |  |
|  |  | Hierarchy | **.85^***^** | .17 | .32 | 1.37 |  |
|  |  | Equality | **-.65^***^** | .15 | -1.11 | -.20 |  |
|  |  | Unity & Communal Sharing | .49^*^ | .15 | .03 | .94 |  |
|  | Unity & Communal Sharing | Family | -.07 | .11 | -.42 | .27 |  |
|  |  | Reciprocity | -.12 | .14 | -.57 | .34 |  |
|  |  | Bravery | -.37 | .13 | -.78 | .05 |  |
|  |  | Hierarchy | .36 | .15 | -.10 | .83 |  |
|  |  | Equality | **-1.14^***^** | .14 | -1.58 | -.70 |  |
|  |  | Property | **-.49^*^** | .15 | -.94 | -.03 |  |
| Negative | Family | Reciprocity | -.41 | .14 | -.85 | .04 |  |
|  |  | Bravery | .00 | .19 | -.58 | .59 |  |
|  |  | Hierarchy | -.15 | .20 | -.77 | .47 |  |
|  |  | Equality | **-1.51^***^** | .20 | -2.13 | -.90 |  |
|  |  | Property | **-1.20^***^** | .21 | -1.86 | -.54 |  |
|  |  | Unity & Communal Sharing | -.20 | .20 | -.82 | .43 |  |
|  | Reciprocity | Family | .41 | .14 | -.04 | .85 |  |
|  |  | Bravery | .41 | .16 | -.10 | .93 |  |
|  |  | Hierarchy | .26 | .20 | -.35 | .87 |  |
|  |  | Equality | **-1.11^***^** | .16 | -1.62 | -.59 |  |
|  |  | Property | **-.79^***^** | .16 | -1.29 | -.30 |  |
|  |  | Unity & Communal Sharing | .21 | .17 | -.31 | .73 |  |
|  | Bravery | Family | .00 | .19 | -.59 | .58 |  |
|  |  | Reciprocity | -.41 | .16 | -.93 | .10 |  |
|  |  | Hierarchy | -.15 | .18 | -.71 | .40 |  |
|  |  | Equality | **-1.52^***^** | .18 | -2.07 | -.97 |  |
|  |  | Property | **-1.21^***^** | .16 | -1.71 | -.70 |  |
|  |  | Unity & Communal Sharing | -.20 | .15 | -.66 | .25 |  |
|  | Hierarchy | Family | .15 | .20 | -.47 | .77 |  |
|  |  | Reciprocity | -.26 | .20 | -.87 | .35 |  |
|  |  | Bravery | .15 | .18 | -.40 | .71 |  |
|  |  | Equality | **-1.37^***^** | .19 | -1.95 | -.78 |  |
|  |  | Property | **-1.05^***^** | .16 | -1.56 | -.54 |  |
|  |  | Unity & Communal Sharing | -.05 | .16 | -.54 | .44 |  |
|  | Equality | Family | **1.51^***^** | .20 | .90 | 2.13 |  |
|  |  | Reciprocity | **1.11^***^** | .16 | .59 | 1.62 |  |
|  |  | Bravery | **1.52^***^** | .18 | .97 | 2.07 |  |
|  |  | Hierarchy | **1.37^***^** | .19 | .78 | 1.95 |  |
|  |  | Property | .31 | .16 | -.20 | .82 |  |
|  |  | Unity & Communal Sharing | **1.32^***^** | .19 | .72 | 1.91 |  |
|  | Property | Family | **1.20^***^** | .21 | .54 | 1.86 |  |
|  |  | Reciprocity | **.79^***^** | .16 | .30 | 1.29 |  |
|  |  | Bravery | **1.21^***^** | .16 | .70 | 1.71 |  |
|  |  | Hierarchy | **1.05^***^** | .16 | .54 | 1.56 |  |
|  |  | Equality | -.31 | .16 | -.82 | .20 |  |
|  |  | Unity & Communal Sharing | **1.00^***^** | .17 | .49 | 1.52 |  |
|  | Unity & Communal Sharing | Family | .20 | .20 | -.43 | .82 |  |
|  |  | Reciprocity | -.21 | .17 | -.73 | .31 |  |
|  |  | Bravery | .20 | .15 | -.25 | .66 |  |
|  |  | Hierarchy | .05 | .16 | -.44 | .54 |  |
|  |  | Equality | **-1.32^***^** | .19 | -1.91 | -.72 |  |
|  |  | Property | **-1.00^***^** | .17 | -1.52 | -.49 |  |
| Based on estimated marginal means | | | | | | | |
| *. The mean difference is significant at the .05 level. ***. The mean difference is significant at the .001 level. | | | | | | | |
| b. Adjustment for multiple comparisons: Bonferroni. | | | | | | | |

**Table C. Multiple Comparisons of Cooperation Difference Scores in Study 2.**

| (I) Domain | (J) Domain | Mean Difference (I-J) | Std. Error | 95% Confidence Interval for Difference^b^ | |  |
| --- | --- | --- | --- | --- | --- | --- |
|  |  |  |  | Lower Bound | Upper Bound |  |
| Family | Reciprocity | .02 | .12 | -.36 | .40 |  |
|  | Bravery | -.06 | .12 | -.44 | .31 |  |
|  | Hierarchy | .37 | .17 | -.16 | .90 |  |
|  | Equality | **-1.25^***^** | .16 | -1.74 | -.77 |  |
|  | Property | **-.68^***^** | .14 | -1.10 | -.25 |  |
|  | Unity & Communal Sharing | -.19 | .15 | -.67 | .30 |  |
| Reciprocity | Family | -.02 | .12 | -.40 | .36 |  |
|  | Bravery | -.08 | .13 | -.48 | .32 |  |
|  | Hierarchy | .35 | .18 | -.21 | .92 |  |
|  | Equality | **-1.27^***^** | .18 | -1.82 | -.72 |  |
|  | Property | **-.69^***^** | .15 | -1.17 | -.22 |  |
|  | Unity & Communal Sharing | -.20 | .17 | -.73 | .32 |  |
| Bravery | Family | .06 | .12 | -.31 | .44 |  |
|  | Reciprocity | .08 | .13 | -.32 | .48 |  |
|  | Hierarchy | .44 | .17 | -.10 | .97 |  |
|  | Equality | **-1.19^***^** | .16 | -1.69 | -.69 |  |
|  | Property | **-.61^***^** | .13 | -1.03 | -.19 |  |
|  | Unity & Communal Sharing | -.12 | .15 | -.59 | .34 |  |
| Hierarchy | Family | -.37 | .17 | -.90 | .16 |  |
|  | Reciprocity | -.35 | .18 | -.92 | .21 |  |
|  | Bravery | -.44 | .17 | -.97 | .10 |  |
|  | Equality | **-1.62^***^** | .17 | -2.17 | -1.08 |  |
|  | Property | **-1.05^***^** | .14 | -1.49 | -.61 |  |
|  | Unity & Communal Sharing | **-.56^***^** | .15 | -1.01 | -.10 |  |
| Equality | Family | **1.25^***^** | .16 | .77 | 1.74 |  |
|  | Reciprocity | **1.27^***^** | .18 | .72 | 1.82 |  |
|  | Bravery | **1.19^***^** | .16 | .69 | 1.69 |  |
|  | Hierarchy | **1.63^***^** | .17 | 1.08 | 2.17 |  |
|  | Property | **.58^*^** | .16 | .09 | 1.07 |  |
|  | Unity & Communal Sharing | **1.07^***^** | .17 | .54 | 1.59 |  |
| Property | Family | **.68^***^** | .14 | .25 | 1.10 |  |
|  | Reciprocity | **.69^***^** | .15 | .22 | 1.17 |  |
|  | Bravery | **.61^***^** | .13 | .19 | 1.03 |  |
|  | Hierarchy | **1.05^***^** | .14 | .61 | 1.49 |  |
|  | Equality | **-.58^*^** | .16 | -1.07 | -.09 |  |
|  | Unity & Communal Sharing | **.49^*^** | .13 | .07 | .91 |  |
| Unity & Communal Sharing | Family | .19 | .15 | -.30 | .67 |  |
|  | Reciprocity | .20 | .17 | -.32 | .73 |  |
|  | Bravery | .12 | .15 | -.34 | .59 |  |
|  | Hierarchy | **.56^***^** | .15 | .10 | 1.01 |  |
|  | Equality | **-1.07^***^** | .17 | -1.59 | -.54 |  |
|  | Property | **-.49^*^** | .13 | -.91 | -.07 |  |
| Based on estimated marginal means | | | | | | |
| *. The mean difference is significant at the .05 level. ***. The mean difference is significant at the .001 level. | | | | | | |
| b. Adjustment for multiple comparisons: Bonferroni. | | | | | | |
