# Supplementary material for: Separate and unequal: Moral domains differ in corresponding social judgments of others
Source: PLoS One. 2026 Jan 8;21(1):e0338026. doi: 10.1371/journal.pone.0338026 (PMC12782401; doi:10.1371/journal.pone.0338026)
Supplement: S5 Appendix — (DOCX) [file pone.0338026.s005.docx]

**S5 Appendix. Study 3 Analyses Not Using Difference Scores.**

**Fig A. Correspondent Inferences by Domain and Valence in Study 3 (No Difference Scores).**


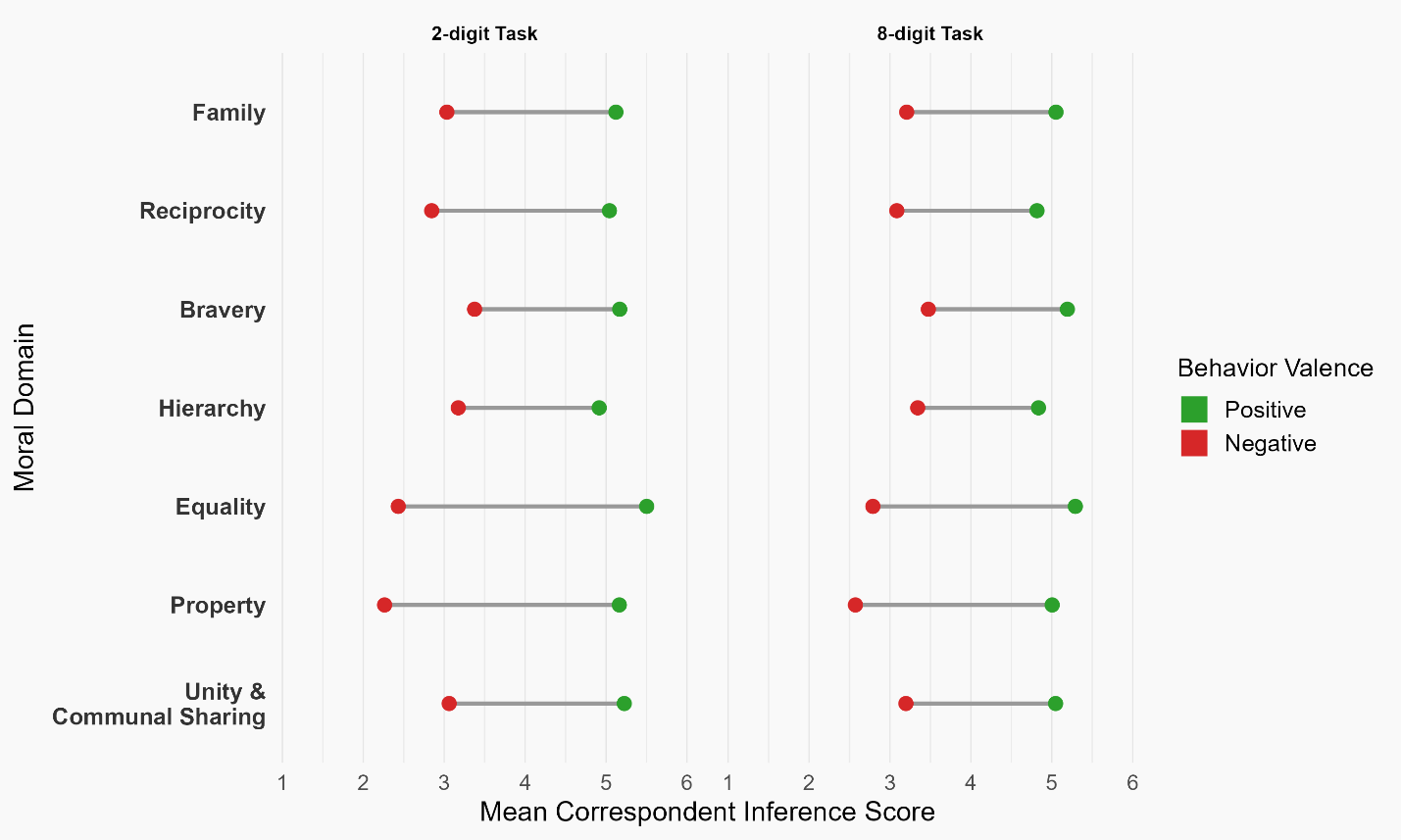


Dumbbell plot shows individual and group-level mean ratings of how principled, ethical, and morally upstanding participants found social targets following each behavior, split by digit task condition. There were no differences in inferences across conditions. As in Study 2, there were main effects of domain and valence such that participants were more likely to make higher ratings about targets’ character following positive behaviors (*M* = 5.06, *SD* = .87) than following negative behaviors (*M* = 2.98, *SD* = 1.09), *F*(1, 103) = 640.08, *η^2^_p_* = .86, *p* < .001.. There was also an interaction effect between valence and domain showing more sensitivity to valence in some domains), *F*(1, 103) = 640.08, *η^2^_p_* = .86, *p* < .001. The directions of these effects collapsed across digit tasks conditions can be seen in Table A.

**Table A. Multiple Comparisons of Study 3 Correspondent Inferences Collapsed Across Conditions (No Difference Scores).**

| Valence | (I) Domain | (J) Domain | Mean Difference (I-J) | Std. Error | 95% Confidence Interval for Difference^b^ | |  |
| --- | --- | --- | --- | --- | --- | --- | --- |
|  |  |  |  |  | Lower Bound | Upper Bound |  |
| Positive | Family | Reciprocity | .16 | .07 | -.07 | .39 |  |
|  |  | Bravery | -.09 | .06 | -.29 | .10 |  |
|  |  | Hierarchy | .21 | .07 | .00 | .42 |  |
|  |  | Equality | **-.31^***^** | .06 | -.49 | -.13 |  |
|  |  | Property | .00 | .06 | -.19 | .19 |  |
|  |  | Unity & Communal Sharing | -.05 | .06 | -.23 | .13 |  |
|  | Reciprocity | Family | -.16 | .07 | -.39 | .07 |  |
|  |  | Bravery | **-.25^*^** | .08 | -.49 | -.01 |  |
|  |  | Hierarchy | .05 | .08 | -.20 | .31 |  |
|  |  | Equality | **-.47^***^** | .07 | -.69 | -.25 |  |
|  |  | Property | -.15 | .07 | -.39 | .08 |  |
|  |  | Unity & Communal Sharing | -.21 | .07 | -.43 | .02 |  |
|  | Bravery | Family | .09 | .06 | -.10 | .29 |  |
|  |  | Reciprocity | **.25^*^** | .08 | .01 | .49 |  |
|  |  | Hierarchy | **.31^***^** | .07 | .09 | .52 |  |
|  |  | Equality | **-.22^***^** | .05 | -.38 | -.06 |  |
|  |  | Property | .10 | .06 | -.09 | .28 |  |
|  |  | Unity & Communal Sharing | .04 | .06 | -.13 | .22 |  |
|  | Hierarchy | Family | -.21 | .07 | -.42 | .00 |  |
|  |  | Reciprocity | -.05 | .08 | -.31 | .20 |  |
|  |  | Bravery | **-.31^***^** | .07 | -.52 | -.09 |  |
|  |  | Equality | **-.52^***^** | .07 | -.74 | -.31 |  |
|  |  | Property | -.21 | .07 | -.43 | .01 |  |
|  |  | Unity & Communal Sharing | -.26^*^ | .07 | -.48 | -.04 |  |
|  | Equality | Family | **.31^***^** | .06 | .13 | .49 |  |
|  |  | Reciprocity | **.47^***^** | .07 | .25 | .69 |  |
|  |  | Bravery | **.22^***^** | .05 | .06 | .38 |  |
|  |  | Hierarchy | **.52^***^** | .07 | .31 | .74 |  |
|  |  | Property | **.31^***^** | .06 | .13 | .49 |  |
|  |  | Unity & Communal Sharing | **.26^***^** | .05 | .12 | .41 |  |
|  | Property | Family | .00 | .06 | -.19 | .19 |  |
|  |  | Reciprocity | .15 | .07 | -.08 | .39 |  |
|  |  | Bravery | -.10 | .06 | -.28 | .09 |  |
|  |  | Hierarchy | .21 | .07 | -.01 | .43 |  |
|  |  | Equality | **-.31^***^** | .06 | -.49 | -.13 |  |
|  |  | Unity & Communal Sharing | -.05 | .06 | -.23 | .13 |  |
|  | Unity & Communal Sharing | Family | .05 | .06 | -.13 | .23 |  |
|  |  | Reciprocity | .21 | .07 | -.02 | .43 |  |
|  |  | Bravery | -.04 | .06 | -.22 | .13 |  |
|  |  | Hierarchy | **.26^*^** | .07 | .04 | .48 |  |
|  |  | Equality | **-.26^***^** | .05 | -.41 | -.12 |  |
|  |  | Property | .05 | .06 | -.13 | .23 |  |
| Negative | Family | Reciprocity | .15 | .09 | -.13 | .44 |  |
|  |  | Bravery | **-.31^*^** | .10 | -.61 | .00 |  |
|  |  | Hierarchy | -.14 | .09 | -.43 | .16 |  |
|  |  | Equality | **.51^***^** | .11 | .18 | .84 |  |
|  |  | Property | **.70^***^** | .09 | .42 | .98 |  |
|  |  | Unity & Communal Sharing | -.01 | .09 | -.30 | .28 |  |
|  | Reciprocity | Family | -.15 | .09 | -.44 | .13 |  |
|  |  | Bravery | **-.46^***^** | .09 | -.74 | -.18 |  |
|  |  | Hierarchy | -.29 | .10 | -.60 | .01 |  |
|  |  | Equality | **.35^*^** | .10 | .05 | .66 |  |
|  |  | Property | **.55^***^** | .08 | .29 | .81 |  |
|  |  | Unity & Communal Sharing | -.16 | .10 | -.46 | .13 |  |
|  | Bravery | Family | **.31^*^** | .10 | .00 | .61 |  |
|  |  | Reciprocity | **.46^***^** | .09 | .18 | .74 |  |
|  |  | Hierarchy | .17 | .11 | -.17 | .50 |  |
|  |  | Equality | **.81^***^** | .12 | .45 | 1.18 |  |
|  |  | Property | **1.01^***^** | .10 | .70 | 1.32 |  |
|  |  | Unity & Communal Sharing | .30 | .10 | -.02 | .61 |  |
|  | Hierarchy | Family | .14 | .09 | -.16 | .43 |  |
|  |  | Reciprocity | .29 | .10 | -.01 | .60 |  |
|  |  | Bravery | -.17 | .11 | -.50 | .17 |  |
|  |  | Equality | **.65^***^** | .12 | .28 | 1.01 |  |
|  |  | Property | **.84^***^** | .09 | .56 | 1.13 |  |
|  |  | Unity & Communal Sharing | .13 | .11 | -.21 | .46 |  |
|  | Equality | Family | **-.51^***^** | .11 | -.84 | -.18 |  |
|  |  | Reciprocity | **-.35^*^** | .10 | -.66 | -.05 |  |
|  |  | Bravery | **-.81^***^** | .12 | -1.18 | -.45 |  |
|  |  | Hierarchy | **-.65^***^** | .12 | -1.01 | -.28 |  |
|  |  | Property | .19 | .10 | -.11 | .49 |  |
|  |  | Unity & Communal Sharing | **-.52^***^** | .11 | -.86 | -.17 |  |
|  | Property | Family | **-.70^***^** | .09 | -.98 | -.42 |  |
|  |  | Reciprocity | **-.55^***^** | .08 | -.81 | -.29 |  |
|  |  | Bravery | **-1.01^***^** | .10 | -1.32 | -.70 |  |
|  |  | Hierarchy | **-.84^***^** | .09 | -1.13 | -.56 |  |
|  |  | Equality | -.19 | .10 | -.49 | .11 |  |
|  |  | Unity & Communal Sharing | **-.71^***^** | .10 | -1.02 | -.40 |  |
|  | Unity & Communal Sharing | Family | .01 | .09 | -.28 | .30 |  |
|  |  | Reciprocity | .16 | .10 | -.13 | .46 |  |
|  |  | Bravery | -.30 | .10 | -.61 | .02 |  |
|  |  | Hierarchy | -.13 | .11 | -.46 | .21 |  |
|  |  | Equality | **.52^***^** | .11 | .17 | .86 |  |
|  |  | Property | **.71^***^** | .10 | .40 | 1.02 |  |
| Based on estimated marginal means. Table shows means collapsed across digit task conditions. | | | | | | | |
| *. The mean difference is significant at the .05 level. ***. The mean difference is significant at the .001 level. | | | | | | | |
| b. Adjustment for multiple comparisons: Bonferroni. | | | | | | | |

**Fig B. Dispositional and Situational Attributions by Domain and Valence in Study 3 (No Difference Scores).**


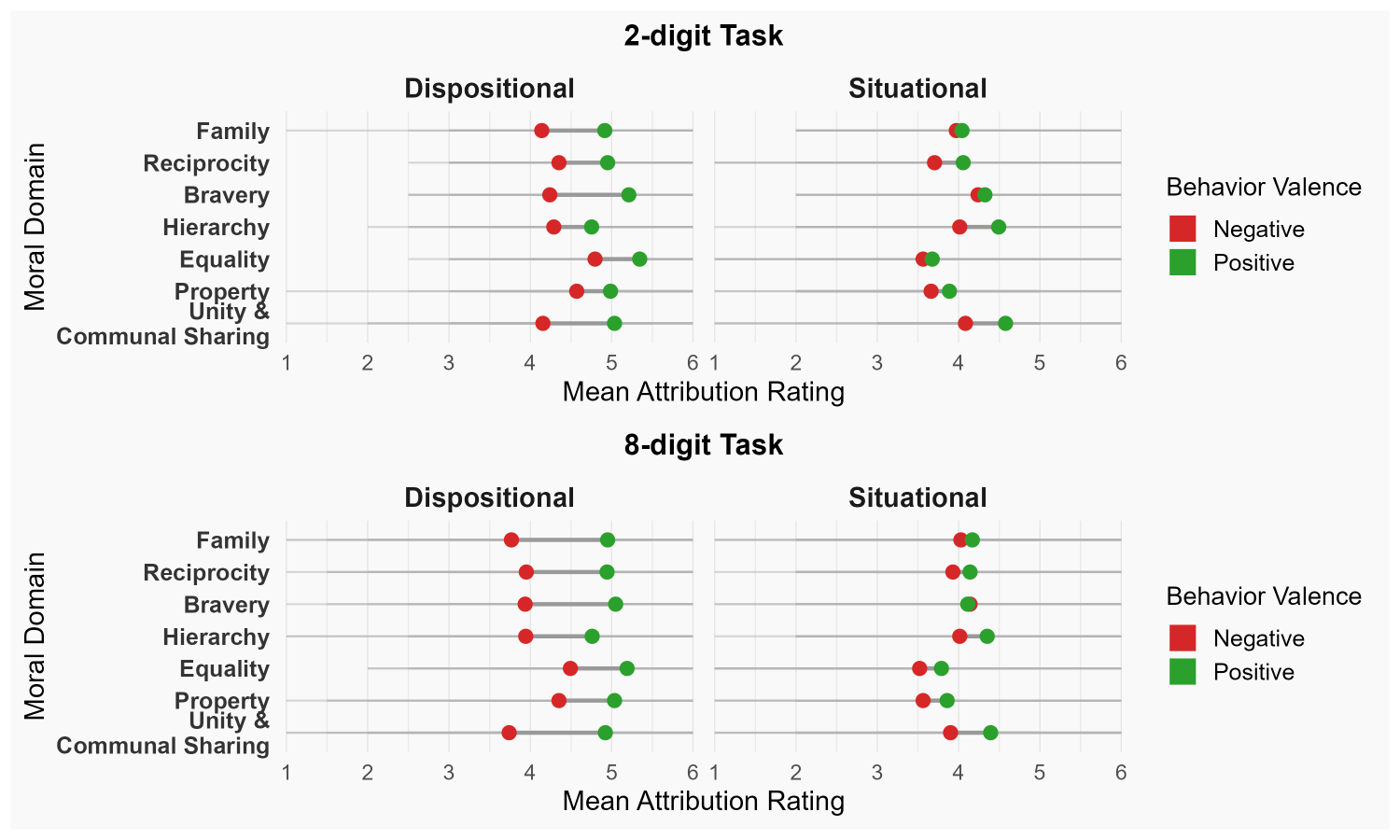


Dumbbell plot shows dispositional and situational attributions across behaviors split by digit task condition. When separating out dispositional and situational attribution, there was an observable main effect of condition such that participants who were in the 2-digit task were more likely to make dispositional attributions (*M* = 4.69, *SD* = .84) than those in the 8-digit task (*M* = 4.60, *SD* = .91), *F*(1, 140) = 4.59, *p* = .03, *η^2^_p_* = .03. There was also an interaction between condition and valence indicating that the differences in dispositional attribution between conditions were more pronounced for negative behaviors, with those in the 2-digit task (*M* = 4.36, *SD* = .93) making stronger dispositional attributions following negative behaviors than those in the 8-digit task (*M* = 4.03, *SD* = 1.03), *F*(1, 140) = 6.58, *p* = .01, *η^2^_p_* = .05. There were no differences between conditions when looking solely at situational attribution. Our use of difference scores in the main text provides an overall sense of dispositional attribution when accounting for situational attribution, rendering these minor attribution-level differences moot. However, these small differences in dispositional attribution of negative behaviors by condition may be in line with literature suggesting that individuals less burdened by cognitive load may engage in more deliberate social characterization and cognitive corrections of social impressions [1][2]. When not under cognitive burden, individuals may be more likely to make stronger dispositional judgments of negative traits in line with findings that negative information may matter more to characterizations of others and be more impactful in belief updating [3][4][5]. Dispositional and situational attributions collapsed across conditions are shown in Tables B and C below.

**Table B. Multiple Comparisons of Study 3 Dispositional Attribution Collapsed Across Conditions (No Difference Scores).**

| Valence | (I) Domain | (J) Domain | Mean Difference (I-J) | Std. Error | 95% Confidence Interval for Difference^b^ | |  |
| --- | --- | --- | --- | --- | --- | --- | --- |
|  |  |  |  |  | Lower Bound | Upper Bound |  |
| Positive | Family | Reciprocity | -.01 | .05 | -.18 | .16 |  |
|  |  | Bravery | **-.20^*^** | .06 | -.37 | -.03 |  |
|  |  | Hierarchy | .18 | .06 | -.01 | .36 |  |
|  |  | Equality | **-.33^***^** | .06 | -.51 | -.16 |  |
|  |  | Property | -.08 | .06 | -.25 | .10 |  |
|  |  | Unity & Communal Sharing | -.05 | .06 | -.22 | .13 |  |
|  | Reciprocity | Family | .01 | .05 | -.16 | .18 |  |
|  |  | Bravery | **-.18^*^** | .05 | -.35 | -.01 |  |
|  |  | Hierarchy | **.19^*^** | .06 | .01 | .37 |  |
|  |  | Equality | **-.32^***^** | .05 | -.49 | -.15 |  |
|  |  | Property | -.06 | .06 | -.25 | .12 |  |
|  |  | Unity & Communal Sharing | -.03 | .06 | -.20 | .14 |  |
|  | Bravery | Family | **.20^*^** | .06 | .03 | .37 |  |
|  |  | Reciprocity | **.18^*^** | .05 | .01 | .35 |  |
|  |  | Hierarchy | **.37^***^** | .06 | .17 | .57 |  |
|  |  | Equality | -.14 | .05 | -.28 | .01 |  |
|  |  | Property | .12 | .06 | -.05 | .29 |  |
|  |  | Unity & Communal Sharing | .15 | .05 | .00 | .30 |  |
|  | Hierarchy | Family | -.18 | .06 | -.36 | .01 |  |
|  |  | Reciprocity | **-.19^*^** | .06 | -.37 | -.01 |  |
|  |  | Bravery | **-.37^***^** | .06 | -.57 | -.17 |  |
|  |  | Equality | **-.51^***^** | .06 | -.71 | -.31 |  |
|  |  | Property | **-.25^***^** | .06 | -.44 | -.07 |  |
|  |  | Unity & Communal Sharing | **-.22^*^** | .06 | -.42 | -.03 |  |
|  | Equality | Family | **.33^***^** | .06 | .16 | .51 |  |
|  |  | Reciprocity | **.32^***^** | .05 | .15 | .49 |  |
|  |  | Bravery | .14 | .05 | -.01 | .28 |  |
|  |  | Hierarchy | **.51^***^** | .06 | .31 | .71 |  |
|  |  | Property | **.26^***^** | .06 | .09 | .43 |  |
|  |  | Unity & Communal Sharing | **.29^***^** | .05 | .14 | .44 |  |
|  | Property | Family | .08 | .06 | -.10 | .25 |  |
|  |  | Reciprocity | .06 | .06 | -.12 | .25 |  |
|  |  | Bravery | -.12 | .06 | -.29 | .05 |  |
|  |  | Hierarchy | **.25^***^** | .06 | .07 | .44 |  |
|  |  | Equality | **-.26^***^** | .06 | -.43 | -.09 |  |
|  |  | Unity & Communal Sharing | .03 | .06 | -.16 | .22 |  |
|  | Unity & Communal Sharing | Family | .05 | .06 | -.13 | .22 |  |
|  |  | Reciprocity | .03 | .06 | -.14 | .20 |  |
|  |  | Bravery | -.15 | .05 | -.30 | .00 |  |
|  |  | Hierarchy | **.22^*^** | .06 | .03 | .42 |  |
|  |  | Equality | **-.29^***^** | .05 | -.44 | -.14 |  |
|  |  | Property | -.03 | .06 | -.22 | .16 |  |
| Negative | Family | Reciprocity | -.20 | .08 | -.43 | .04 |  |
|  |  | Bravery | -.13 | .10 | -.43 | .16 |  |
|  |  | Hierarchy | -.16 | .09 | -.46 | .13 |  |
|  |  | Equality | **-.69^***^** | .09 | -.98 | -.40 |  |
|  |  | Property | **-.51^***^** | .09 | -.78 | -.24 |  |
|  |  | Unity & Communal Sharing | .01 | .09 | -.28 | .30 |  |
|  | Reciprocity | Family | .20 | .08 | -.04 | .43 |  |
|  |  | Bravery | .06 | .07 | -.17 | .30 |  |
|  |  | Hierarchy | .04 | .09 | -.24 | .31 |  |
|  |  | Equality | **-.49^***^** | .08 | -.75 | -.24 |  |
|  |  | Property | **-.31^***^** | .08 | -.56 | -.06 |  |
|  |  | Unity & Communal Sharing | .20 | .09 | -.08 | .48 |  |
|  | Bravery | Family | .13 | .10 | -.16 | .43 |  |
|  |  | Reciprocity | -.06 | .07 | -.30 | .17 |  |
|  |  | Hierarchy | -.03 | .08 | -.28 | .23 |  |
|  |  | Equality | **-.56^***^** | .09 | -.83 | -.29 |  |
|  |  | Property | **-.37^***^** | .10 | -.67 | -.08 |  |
|  |  | Unity & Communal Sharing | .14 | .09 | -.15 | .43 |  |
|  | Hierarchy | Family | .16 | .09 | -.13 | .46 |  |
|  |  | Reciprocity | -.04 | .09 | -.31 | .24 |  |
|  |  | Bravery | .03 | .08 | -.23 | .28 |  |
|  |  | Equality | **-.53^***^** | .09 | -.81 | -.25 |  |
|  |  | Property | **-.35^***^** | .09 | -.62 | -.07 |  |
|  |  | Unity & Communal Sharing | .17 | .10 | -.14 | .47 |  |
|  | Equality | Family | **.69^***^** | .09 | .40 | .98 |  |
|  |  | Reciprocity | **.49^***^** | .08 | .24 | .75 |  |
|  |  | Bravery | **.56^***^** | .09 | .29 | .83 |  |
|  |  | Hierarchy | **.53^***^** | .09 | .25 | .81 |  |
|  |  | Property | .18 | .08 | -.07 | .44 |  |
|  |  | Unity & Communal Sharing | **.70^***^** | .10 | .40 | .99 |  |
|  | Property | Family | **.51^***^** | .09 | .24 | .78 |  |
|  |  | Reciprocity | **.31^***^** | .08 | .06 | .56 |  |
|  |  | Bravery | **.37^***^** | .10 | .08 | .67 |  |
|  |  | Hierarchy | **.35^***^** | .09 | .07 | .62 |  |
|  |  | Equality | -.18 | .08 | -.44 | .07 |  |
|  |  | Unity & Communal Sharing | **.51^***^** | .10 | .22 | .81 |  |
|  | Unity & Communal Sharing | Family | -.01 | .09 | -.30 | .28 |  |
|  |  | Reciprocity | -.20 | .09 | -.48 | .08 |  |
|  |  | Bravery | -.14 | .09 | -.43 | .15 |  |
|  |  | Hierarchy | -.17 | .10 | -.47 | .14 |  |
|  |  | Equality | **-.70^***^** | .10 | -.99 | -.40 |  |
|  |  | Property | **-.51^***^** | .10 | -.81 | -.22 |  |
| Based on estimated marginal means. Table shows means collapsed across digit task conditions. | | | | | | | |
| *. The mean difference is significant at the .05 level. ***. The mean difference is significant at the .001 level. | | | | | | | |
| b. Adjustment for multiple comparisons: Bonferroni. | | | | | | | |

**Table C. Multiple Comparisons of Study 3 Situational Attribution Collapsed Across Conditions (No Difference Scores).**

| Valence | (I) Domain | (J) Domain | Mean Difference (I-J) | Std. Error | 95% Confidence Interval for Difference^b^ | |  |
| --- | --- | --- | --- | --- | --- | --- | --- |
|  |  |  |  |  | Lower Bound | Upper Bound |  |
| Positive | Family | Reciprocity | .01 | .11 | -.33 | .34 |  |
|  |  | Bravery | -.11 | .11 | -.46 | .23 |  |
|  |  | Hierarchy | -.32 | .11 | -.67 | .04 |  |
|  |  | Equality | .37 | .12 | -3.96E-6 | .75 |  |
|  |  | Property | .23 | .09 | -.06 | .52 |  |
|  |  | Unity & Communal Sharing | **-.38^*^** | .11 | -.71 | -.05 |  |
|  | Reciprocity | Family | -.01 | .11 | -.34 | .33 |  |
|  |  | Bravery | -.12 | .11 | -.46 | .22 |  |
|  |  | Hierarchy | **-.32^*^** | .10 | -.62 | -.02 |  |
|  |  | Equality | .37 | .13 | -.02 | .75 |  |
|  |  | Property | .23 | .11 | -.10 | .55 |  |
|  |  | Unity & Communal Sharing | **-.39^***^** | .10 | -.70 | -.08 |  |
|  | Bravery | Family | .11 | .11 | -.23 | .46 |  |
|  |  | Reciprocity | .12 | .11 | -.22 | .46 |  |
|  |  | Hierarchy | -.20 | .10 | -.52 | .11 |  |
|  |  | Equality | **.49^***^** | .12 | .11 | .86 |  |
|  |  | Property | **.35^*^** | .11 | .00 | .69 |  |
|  |  | Unity & Communal Sharing | -.27 | .11 | -.60 | .07 |  |
|  | Hierarchy | Family | .32 | .11 | -.04 | .67 |  |
|  |  | Reciprocity | .**32^*^** | .10 | .02 | .62 |  |
|  |  | Bravery | .20 | .10 | -.11 | .52 |  |
|  |  | Equality | **.69^***^** | .12 | .33 | 1.05 |  |
|  |  | Property | **.55^***^** | .11 | .20 | .90 |  |
|  |  | Unity & Communal Sharing | -.06 | .10 | -.39 | .26 |  |
|  | Equality | Family | -.37 | .12 | -.75 | 3.96E-6 |  |
|  |  | Reciprocity | -.37 | .13 | -.75 | .02 |  |
|  |  | Bravery | **-.49^***^** | .12 | -.86 | -.11 |  |
|  |  | Hierarchy | **-.69^***^** | .12 | -1.05 | -.33 |  |
|  |  | Property | -.14 | .11 | -.48 | .20 |  |
|  |  | Unity & Communal Sharing | **-.75^***^** | .13 | -1.15 | -.35 |  |
|  | Property | Family | -.23 | .09 | -.52 | .06 |  |
|  |  | Reciprocity | -.23 | .11 | -.55 | .10 |  |
|  |  | Bravery | **-.35^*^** | .11 | -.69 | .00 |  |
|  |  | Hierarchy | **-.55^***^** | .11 | -.90 | -.20 |  |
|  |  | Equality | .14 | .11 | -.20 | .48 |  |
|  |  | Unity & Communal Sharing | **-.61^***^** | .11 | -.97 | -.26 |  |
|  | Unity & Communal Sharing | Family | **.38^*^** | .11 | .05 | .71 |  |
|  |  | Reciprocity | **.39^***^** | .10 | .08 | .70 |  |
|  |  | Bravery | .27 | .11 | -.07 | .60 |  |
|  |  | Hierarchy | .06 | .10 | -.26 | .39 |  |
|  |  | Equality | **.75^***^** | .13 | .35 | 1.15 |  |
|  |  | Property | **.61^***^** | .11 | .26 | .97 |  |
| Negative | Family | Reciprocity | .18 | .11 | -.14 | .51 |  |
|  |  | Bravery | -.19 | .12 | -.56 | .18 |  |
|  |  | Hierarchy | -.01 | .12 | -.38 | .35 |  |
|  |  | Equality | **.46^*^** | .12 | .08 | .83 |  |
|  |  | Property | **.39^*^** | .11 | .06 | .71 |  |
|  |  | Unity & Communal Sharing | .01 | .11 | -.34 | .35 |  |
|  | Reciprocity | Family | -.18 | .11 | -.51 | .14 |  |
|  |  | Bravery | **-.37^*^** | .11 | -.70 | -.05 |  |
|  |  | Hierarchy | -.20 | .10 | -.52 | .12 |  |
|  |  | Equality | .27 | .10 | -.04 | .59 |  |
|  |  | Property | .20 | .10 | -.11 | .52 |  |
|  |  | Unity & Communal Sharing | -.18 | .10 | -.50 | .15 |  |
|  | Bravery | Family | .19 | .12 | -.18 | .56 |  |
|  |  | Reciprocity | **.37^*^** | .11 | .05 | .70 |  |
|  |  | Hierarchy | .18 | .10 | -.14 | .49 |  |
|  |  | Equality | **.65^***^** | .11 | .30 | 1.00 |  |
|  |  | Property | **.58^***^** | .12 | .22 | .93 |  |
|  |  | Unity & Communal Sharing | .20 | .11 | -.15 | .55 |  |
|  | Hierarchy | Family | .01 | .12 | -.35 | .38 |  |
|  |  | Reciprocity | .20 | .10 | -.12 | .52 |  |
|  |  | Bravery | -.18 | .10 | -.49 | .14 |  |
|  |  | Equality | **.47^***^** | .12 | .10 | .84 |  |
|  |  | Property | **.40^*^** | .12 | .04 | .76 |  |
|  |  | Unity & Communal Sharing | .02 | .11 | -.33 | .38 |  |
|  | Equality | Family | **-.46^*^** | .12 | -.83 | -.08 |  |
|  |  | Reciprocity | -.27 | .10 | -.59 | .04 |  |
|  |  | Bravery | **-.65^***^** | .11 | -1.00 | -.30 |  |
|  |  | Hierarchy | **-.47^***^** | .12 | -.84 | -.10 |  |
|  |  | Property | -.07 | .11 | -.41 | .27 |  |
|  |  | Unity & Communal Sharing | **-.45^***^** | .12 | -.81 | -.09 |  |
|  | Property | Family | **-.39^*^** | .11 | -.71 | -.06 |  |
|  |  | Reciprocity | -.20 | .10 | -.52 | .11 |  |
|  |  | Bravery | **-.58^***^** | .12 | -.93 | -.22 |  |
|  |  | Hierarchy | **-.40^*^** | .12 | -.76 | -.04 |  |
|  |  | Equality | .07 | .11 | -.27 | .41 |  |
|  |  | Unity & Communal Sharing | **-.38^*^** | .12 | -.74 | -.02 |  |
|  | Unity & Communal Sharing | Family | -.01 | .11 | -.35 | .34 |  |
|  |  | Reciprocity | .18 | .10 | -.15 | .50 |  |
|  |  | Bravery | -.20 | .11 | -.55 | .15 |  |
|  |  | Hierarchy | -.02 | .11 | -.38 | .33 |  |
|  |  | Equality | **.45^***^** | .12 | .09 | .81 |  |
|  |  | Property | **.38^*^** | .12 | .02 | .74 |  |
| Based on estimated marginal means. Table shows means collapsed across digit task conditions. | | | | | | | |
| *. The mean difference is significant at the .05 level. ***. The mean difference is significant at the .001 level. | | | | | | | |
| b. Adjustment for multiple comparisons: Bonferroni. | | | | | | | |

**References**

1. Gilbert DT, Osborne RE. Thinking backward: Some curable and incurable consequences of cognitive busyness. *J Pers Soc Psychol.* 1989;57(6):940–9. doi: 10.1037/0022-3514.57.6.940.
2. Gilbert DT, Pelham BW, Krull DS. On cognitive busyness: When person perceivers meet persons perceived. *J Pers Soc Psychol.* 1988;54(5):733–40. doi: 10.1037/0022-3514.54.5.733.
3. Brambilla M, Carraro L, Castelli L, Sacchi S. Changing impressions: moral character dominates impression updating. *J Exp Soc Psychol*. 2019;82:103843. doi:10.1016/j.jesp.2019.01.003.
4. Wojciszke B, Bazinska R, Jaworski M. On the dominance of moral categories in impression formation. *Pers Soc Psychol Bull.* 1998;24(12):1251–63. doi: 10.1177/01461672982412001.
5. Ybarra O. Naive causal understanding of valenced behaviors and its implications for social information processing. *Psychol Bull.* 2002;128(3):421–41. doi: 10.1037/0033-2909.128.3.421.
