# Supplementary material for: Separate and unequal: Moral domains differ in corresponding social judgments of others
Source: PLoS One. 2026 Jan 8;21(1):e0338026. doi: 10.1371/journal.pone.0338026 (PMC12782401; doi:10.1371/journal.pone.0338026)
Supplement: S6 Appendix — (DOCX) [file pone.0338026.s006.docx]

**S6 Appendix. Multiple Comparisons for Reported Analyses in Study 3.**

**Table A. Multiple Comparisons of Study 3 Correspondent Inference Difference Scores.**

| Condition | (I) Domain | (J) Domain | Mean Difference (I-J) | | Std. Error | 95% Confidence Interval for Difference^b^ | |  |
| --- | --- | --- | --- | --- | --- | --- | --- | --- |
|  |  |  |  |  |  | Lower Bound | Upper Bound |  |
| 2-digit Task | Family | Reciprocity | | -.11 | .18 | -.65 | .44 |  |
|  |  | Bravery | | .30 | .18 | -.25 | .84 |  |
|  |  | Hierarchy | | .35 | .19 | -.23 | .93 |  |
|  |  | Equality | | **-.98^***^** | .17 | -1.52 | -.45 |  |
|  |  | Property | | **-.81^***^** | .16 | -1.32 | -.31 |  |
|  |  | Unity & Communal Sharing | | -.08 | .18 | -.62 | .47 |  |
|  | Reciprocity | Family | | .11 | .18 | -.44 | .65 |  |
|  |  | Bravery | | .40 | .18 | -.15 | .95 |  |
|  |  | Hierarchy | | .46 | .21 | -.20 | 1.11 |  |
|  |  | Equality | | **-.87^***^** | .19 | -1.46 | -.28 |  |
|  |  | Property | | **-.70^***^** | .18 | -1.26 | -.14 |  |
|  |  | Unity & Communal Sharing | | .03 | .18 | -.52 | .58 |  |
|  | Bravery | Family | | -.30 | .18 | -.84 | .25 |  |
|  |  | Reciprocity | | -.40 | .18 | -.95 | .15 |  |
|  |  | Hierarchy | | .05 | .21 | -.60 | .70 |  |
|  |  | Equality | | **-1.28^***^** | .18 | -1.83 | -.72 |  |
|  |  | Property | | **-1.11^***^** | .18 | -1.66 | -.55 |  |
|  |  | Unity & Communal Sharing | | -.37 | .17 | -.90 | .16 |  |
|  | Hierarchy | Family | | -.35 | .19 | -.93 | .23 |  |
|  |  | Reciprocity | | -.46 | .21 | -1.11 | .20 |  |
|  |  | Bravery | | -.05 | .21 | -.70 | .60 |  |
|  |  | Equality | | **-1.33^***^** | .22 | -2.00 | -.66 |  |
|  |  | Property | | **-1.16^***^** | .19 | -1.74 | -.58 |  |
|  |  | Unity & Communal Sharing | | -.42 | .21 | -1.08 | .23 |  |
|  | Equality | Family | | **.98^***^** | .17 | .45 | 1.52 |  |
|  |  | Reciprocity | | **.87^***^** | .19 | .28 | 1.46 |  |
|  |  | Bravery | | **1.28^***^** | .18 | .72 | 1.83 |  |
|  |  | Hierarchy | | **1.33^***^** | .22 | .66 | 2.00 |  |
|  |  | Property | | .17 | .17 | -.34 | .68 |  |
|  |  | Unity & Communal Sharing | | **.91^***^** | .17 | .37 | 1.44 |  |
|  | Property | Family | | **.81^***^** | .16 | .31 | 1.32 |  |
|  |  | Reciprocity | | **.70^***^** | .18 | .14 | 1.26 |  |
|  |  | Bravery | | **1.11^***^** | .18 | .55 | 1.66 |  |
|  |  | Hierarchy | | **1.16^***^** | .19 | .58 | 1.74 |  |
|  |  | Equality | | -.17 | .17 | -.68 | .34 |  |
|  |  | Unity & Communal Sharing | | **.74^***^** | .17 | .20 | 1.28 |  |
|  | Unity & Communal Sharing | Family | | .08 | .18 | -.47 | .62 |  |
|  |  | Reciprocity | | -.03 | .18 | -.58 | .52 |  |
|  |  | Bravery | | .37 | .17 | -.16 | .90 |  |
|  |  | Hierarchy | | .42 | .21 | -.23 | 1.08 |  |
|  |  | Equality | | **-.91^***^** | .17 | -1.44 | -.37 |  |
|  |  | Property | | **-.74^***^** | .17 | -1.28 | -.20 |  |
| 8-digit Task | Family | Reciprocity | | .11 | .18 | -.43 | .66 |  |
|  |  | Bravery | | .13 | .18 | -.42 | .67 |  |
|  |  | Hierarchy | | .35 | .19 | -.23 | .93 |  |
|  |  | Equality | | **-.66^***^** | .17 | -1.19 | -.12 |  |
|  |  | Property | | **-.59^*^** | .16 | -1.09 | -.08 |  |
|  |  | Unity & Communal Sharing | | .00 | .18 | -.55 | .54 |  |
|  | Reciprocity | Family | | -.11 | .18 | -.66 | .43 |  |
|  |  | Bravery | | .01 | .18 | -.54 | .56 |  |
|  |  | Hierarchy | | .24 | .21 | -.42 | .90 |  |
|  |  | Equality | | **-.77^***^** | .19 | -1.36 | -.18 |  |
|  |  | Property | | **-.70^***^** | .18 | -1.26 | -.14 |  |
|  |  | Unity & Communal Sharing | | -.12 | .18 | -.67 | .43 |  |
|  | Bravery | Family | | -.13 | .18 | -.67 | .42 |  |
|  |  | Reciprocity | | -.01 | .18 | -.56 | .54 |  |
|  |  | Hierarchy | | .23 | .21 | -.43 | .88 |  |
|  |  | Equality | | **-.78^***^** | .18 | -1.34 | -.23 |  |
|  |  | Property | | **-.71^***^** | .18 | -1.27 | -.16 |  |
|  |  | Unity & Communal Sharing | | -.13 | .17 | -.66 | .40 |  |
|  | Hierarchy | Family | | -.35 | .19 | -.93 | .23 |  |
|  |  | Reciprocity | | -.24 | .21 | -.90 | .42 |  |
|  |  | Bravery | | -.23 | .21 | -.88 | .43 |  |
|  |  | Equality | | **-1.01^***^** | .22 | -1.68 | -.34 |  |
|  |  | Property | | **-.94^***^** | .19 | -1.52 | -.36 |  |
|  |  | Unity & Communal Sharing | | -.36 | .21 | -1.01 | .30 |  |
|  | Equality | Family | | **.66^***^** | .17 | .12 | 1.19 |  |
|  |  | Reciprocity | | **.77^***^** | .19 | .18 | 1.36 |  |
|  |  | Bravery | | **.78^***^** | .18 | .23 | 1.34 |  |
|  |  | Hierarchy | | **1.01^***^** | .22 | .34 | 1.68 |  |
|  |  | Property | | .07 | .17 | -.44 | .58 |  |
|  |  | Unity & Communal Sharing | | **.65^*^** | .17 | .11 | 1.19 |  |
|  | Property | Family | | **.59^*^** | .16 | .08 | 1.09 |  |
|  |  | Reciprocity | | **.70^***^** | .18 | .14 | 1.26 |  |
|  |  | Bravery | | **.71^***^** | .18 | .16 | 1.27 |  |
|  |  | Hierarchy | | **.94^***^** | .19 | .36 | 1.52 |  |
|  |  | Equality | | -.07 | .17 | -.58 | .44 |  |
|  |  | Unity & Communal Sharing | | **.58^*^** | .17 | .04 | 1.12 |  |
|  | Unity & Communal Sharing | Family | | .00 | .18 | -.54 | .55 |  |
|  |  | Reciprocity | | .12 | .18 | -.43 | .67 |  |
|  |  | Bravery | | .13 | .17 | -.40 | .66 |  |
|  |  | Hierarchy | | .36 | .21 | -.30 | 1.01 |  |
|  |  | Equality | | **-.65^*^** | .17 | -1.19 | -.11 |  |
|  |  | Property | | **-.58^*^** | .17 | -1.12 | -.04 |  |
| Based on estimated marginal means | | | | | | | | |
| *. The mean difference is significant at the .05 level. ***. The mean difference is significant at the .001 level. | | | | | | | | |
| b. Adjustment for multiple comparisons: Bonferroni. | | | | | | | | |

**Table B. Multiple Comparisons of Attribution Difference Scores in Study 3.**

| Condition | (I) Domain | (J) Domain | Mean Difference (I-J) | Std. Error | 95% Confidence Interval for Difference^b^ | |  |
| --- | --- | --- | --- | --- | --- | --- | --- |
|  |  |  |  |  | Lower Bound | Upper Bound |  |
| 2-digit Task | Family | Reciprocity | -.25 | .14 | -.68 | .18 |  |
|  |  | Bravery | .08 | .16 | -.41 | .57 |  |
|  |  | Hierarchy | .25 | .17 | -.27 | .78 |  |
|  |  | Equality | **-.93^***^** | .19 | -1.53 | -.33 |  |
|  |  | Property | **-.48^*^** | .14 | -.90 | -.06 |  |
|  |  | Unity & Communal Sharing | .26 | .16 | -.22 | .74 |  |
|  | Reciprocity | Family | .25 | .14 | -.18 | .68 |  |
|  |  | Bravery | .33 | .13 | -.09 | .74 |  |
|  |  | Hierarchy | **.50^*^** | .14 | .07 | .93 |  |
|  |  | Equality | **-.68^***^** | .15 | -1.16 | -.20 |  |
|  |  | Property | -.23 | .13 | -.64 | .18 |  |
|  |  | Unity & Communal Sharing | **.51^*^** | .15 | .04 | .97 |  |
|  | Bravery | Family | -.08 | .16 | -.57 | .41 |  |
|  |  | Reciprocity | -.33 | .13 | -.74 | .09 |  |
|  |  | Hierarchy | .18 | .15 | -.28 | .63 |  |
|  |  | Equality | **-1.01^***^** | .16 | -1.52 | -.50 |  |
|  |  | Property | **-.56^*^** | .16 | -1.05 | -.07 |  |
|  |  | Unity & Communal Sharing | .18 | .14 | -.26 | .62 |  |
|  | Hierarchy | Family | -.25 | .17 | -.78 | .27 |  |
|  |  | Reciprocity | **-.50^*^** | .14 | -.93 | -.07 |  |
|  |  | Bravery | -.18 | .15 | -.63 | .28 |  |
|  |  | Equality | **-1.18^***^** | .18 | -1.75 | -.61 |  |
|  |  | Property | **-.74^***^** | .17 | -1.26 | -.21 |  |
|  |  | Unity & Communal Sharing | .00 | .17 | -.52 | .53 |  |
|  | Equality | Family | **.93^***^** | .19 | .33 | 1.53 |  |
|  |  | Reciprocity | **.68^***^** | .15 | .20 | 1.16 |  |
|  |  | Bravery | **1.01^***^** | .16 | .50 | 1.52 |  |
|  |  | Hierarchy | **1.18^***^** | .18 | .61 | 1.75 |  |
|  |  | Property | .45 | .16 | -.06 | .96 |  |
|  |  | Unity & Communal Sharing | **1.19^***^** | .18 | .63 | 1.74 |  |
|  | Property | Family | **.48^*^** | .14 | .06 | .90 |  |
|  |  | Reciprocity | .23 | .13 | -.18 | .64 |  |
|  |  | Bravery | **.56^*^** | .16 | .07 | 1.05 |  |
|  |  | Hierarchy | **.74^*^** | .17 | .21 | 1.26 |  |
|  |  | Equality | -.45 | .16 | -.96 | .06 |  |
|  |  | Unity & Communal Sharing | **.74^***^** | .17 | .22 | 1.26 |  |
|  | Unity & Communal Sharing | Family | -.26 | .16 | -.74 | .22 |  |
|  |  | Reciprocity | **-.51^*^** | .15 | -.97 | -.04 |  |
|  |  | Bravery | -.18 | .14 | -.62 | .26 |  |
|  |  | Hierarchy | .00 | .17 | -.53 | .52 |  |
|  |  | Equality | **-1.19^***^** | .18 | -1.74 | -.63 |  |
|  |  | Property | **-.74^***^** | .17 | -1.26 | -.22 |  |
| 8-digit Task | Family | Reciprocity | -.15 | .14 | -.58 | .28 |  |
|  |  | Bravery | -.11 | .16 | -.59 | .38 |  |
|  |  | Hierarchy | .09 | .17 | -.43 | .61 |  |
|  |  | Equality | **-.93^***^** | .19 | -1.53 | -.32 |  |
|  |  | Property | **-.72^***^** | .14 | -1.14 | -.30 |  |
|  |  | Unity & Communal Sharing | .08 | .16 | -.40 | .56 |  |
|  | Reciprocity | Family | .15 | .14 | -.28 | .58 |  |
|  |  | Bravery | .05 | .13 | -.37 | .46 |  |
|  |  | Hierarchy | .24 | .14 | -.19 | .67 |  |
|  |  | Equality | **-.77^***^** | .15 | -1.25 | -.30 |  |
|  |  | Property | **-.57^***^** | .13 | -.98 | -.16 |  |
|  |  | Unity & Communal Sharing | .23 | .15 | -.24 | .69 |  |
|  | Bravery | Family | .11 | .16 | -.38 | .59 |  |
|  |  | Reciprocity | -.05 | .13 | -.46 | .37 |  |
|  |  | Hierarchy | .20 | .15 | -.26 | .66 |  |
|  |  | Equality | **-.82^***^** | .16 | -1.33 | -.31 |  |
|  |  | Property | **-.62^***^** | .16 | -1.11 | -.12 |  |
|  |  | Unity & Communal Sharing | .18 | .14 | -.26 | .62 |  |
|  | Hierarchy | Family | -.09 | .17 | -.61 | .43 |  |
|  |  | Reciprocity | -.24 | .14 | -.67 | .19 |  |
|  |  | Bravery | -.20 | .15 | -.66 | .26 |  |
|  |  | Equality | **-1.02^***^** | .18 | -1.59 | -.45 |  |
|  |  | Property | **-.81^***^** | .17 | -1.34 | -.29 |  |
|  |  | Unity & Communal Sharing | -.01 | .17 | -.54 | .51 |  |
|  | Equality | Family | **.93^***^** | .19 | .32 | 1.53 |  |
|  |  | Reciprocity | **.77^***^** | .15 | .30 | 1.25 |  |
|  |  | Bravery | **.82^***^** | .16 | .31 | 1.33 |  |
|  |  | Hierarchy | **1.02^***^** | .18 | .45 | 1.59 |  |
|  |  | Property | .20 | .16 | -.30 | .71 |  |
|  |  | Unity & Communal Sharing | **1.00^***^** | .18 | .45 | 1.56 |  |
|  | Property | Family | **.72^***^** | .14 | .30 | 1.14 |  |
|  |  | Reciprocity | **.57^***^** | .13 | .16 | .98 |  |
|  |  | Bravery | **.62^***^** | .16 | .12 | 1.11 |  |
|  |  | Hierarchy | **.81^***^** | .17 | .29 | 1.34 |  |
|  |  | Equality | -.20 | .16 | -.71 | .30 |  |
|  |  | Unity & Communal Sharing | **.80^***^** | .17 | .28 | 1.32 |  |
|  | Unity & Communal Sharing | Family | -.08 | .16 | -.56 | .40 |  |
|  |  | Reciprocity | -.23 | .15 | -.69 | .24 |  |
|  |  | Bravery | -.18 | .14 | -.62 | .26 |  |
|  |  | Hierarchy | .01 | .17 | -.51 | .54 |  |
|  |  | Equality | **-1.00^***^** | .18 | -1.56 | -.45 |  |
|  |  | Property | **-.80^***^** | .17 | -1.32 | -.28 |  |
| Based on estimated marginal means | | | | | | | |
| *. The mean difference is significant at the .05 level. ***. The mean difference is significant at the .001 level. | | | | | | | |
| b. Adjustment for multiple comparisons: Bonferroni. | | | | | | | |
