# Supplementary material for: Separate and unequal: Moral domains differ in corresponding social judgments of others
Source: PLoS One. 2026 Jan 8;21(1):e0338026. doi: 10.1371/journal.pone.0338026 (PMC12782401; doi:10.1371/journal.pone.0338026)
Supplement: S7 Appendix — (DOCX) [file pone.0338026.s007.docx]

**Appendix 7. Means and Correlations Between Measures in Studies 1 – 3**

**Table A. Means for Individual Variables in Studies 1 – 3.**

|  | | |  | Study 1 | | |  | Study 2 | | | Study 3 | | | |
| --- | --- | --- | --- | --- | --- | --- | --- | --- | --- | --- | --- | --- | --- | --- |
|  | | Variable | *N* | | *M* | *SD* | *N* | | *M* | *SD* | | *N* | *M* | *SD* |
|  | How Principled Target is | | 60 | | 4.05 | .43 | 104 | | 4.04 | .39 | | 142 | 4.04 | .40 |
|  | How Ethical Target is | | 60 | | 4.08 | .44 | 104 | | 4.03 | .39 | | 142 | 4.05 | .39 |
|  | How Morally Upstanding Target is | | 60 | | 4.05 | .46 | 104 | | 3.99 | .37 | | 142 | 4.04 | .44 |
|  | Dispositional Attribution (base item) | | 60 | | 4.50 | .45 | 104 | | 4.65 | .51 | | 142 | 4.67 | .57 |
|  | Representativeness of Behavior | | 60 | | 4.26 | .54 | 104 | | 4.41 | .56 | | 142 | 4.53 | .57 |
|  | Situational Attribution | | 60 | | 3.96 | .66 | 104 | | 4.06 | .74 | | 142 | 4.01 | .71 |
|  | Willingness to share a secret | |  | |  |  | 104 | | 3.02 | .83 | |  |  |  |
|  | Trust to solve a dispute | |  | |  |  | 104 | | 3.54 | .48 | |  |  |  |
|  | Willingness to seek advice | |  | |  |  | 104 | | 3.63 | .47 | |  |  |  |
|  | Willingness to share a car | |  | |  |  | 104 | | 3.92 | .66 | |  |  |  |

**Table B. Correlation Table of Measures Included in Study 1.**

|  | How Principled Target is | How Ethical Target is | How Morally Upstanding Target is | Dispositional Attribution (base item) | Representativeness of Behavior | Situational Attribution |
| --- | --- | --- | --- | --- | --- | --- |
| How Principled Target Is | 1 | **.94^***^** | **.93^***^** | .05 | -.20 | **.39^**^** |
| How Ethical Target is | **.94^***^** | 1 | **.95^***^** | .00 | -.24 | **.41^**^** |
| How Morally Upstanding Target is | **.93^***^** | **.95^***^** | 1 | .00 | -.22 | **.39^**^** |
| Dispositional Attribution (base item) | .05 | .00 | .00 | 1 | **.69^***^** | .11 |
| Representativeness of Behavior | -.20 | -.24 | -.22 | **.69^**^** | 1 | -.15 |
| Situational Attribution | **.39^**^** | **.41^**^** | **.39^**^** | .11 | -.15 | 1 |

Pearson r correlations for measures included in Study 1. *N* = 60.

**. Correlation is significant at the 0.01 level (2-tailed). ***. Correlation is significant at the .001 level.

**Table C. Correlation Table of Measures Included in Study 2.**

|  | How Principled Target is | How Ethical Target is | How Morally Upstanding Target is | Disp. Attribution (base item) | Represent. of Behavior | Sit. Attribution | Willing to Share Secret | Trust to Solve Dispute | Willing to Seek Advice | Willing to Share Car |
| --- | --- | --- | --- | --- | --- | --- | --- | --- | --- | --- |
| How Principled Target Is | 1 | **.90***** | **.89***** | .09 | -.13 | **.24*** | .03 | **.35***** | **.53***** | **.32***** |
| How Ethical Target is | **.90***** | 1 | **.89***** | .11 | -.11 | .17 | .03 | **.36***** | **.50***** | **.38***** |
| How Morally Upstanding Target is | **.89***** | **.89***** | 1 | **.19*** | -.04 | **.19*** | .08 | **.43***** | **.54***** | **.40***** |
| Disp. Attribution (base item) | .09 | .11 | .19* | 1 | **.77***** | .11 | -.10 | -.08 | -.09 | .05 |
| Represent. of Behavior | -.13 | -.11 | -.04 | **.77***** | 1 | -.08 | -.12 | -.18 | **-.25**** | -.11 |
| Sit. Attribution | **.24*** | .17 | .**19*** | .11 | -.08 | 1 | .03 | .03 | .06 | .15 |
| Willing to Share Secret | .03 | .03 | .08 | -.10 | -.12 | .03 | 1 | **.61***** | **.45***** | **.28**** |
| Trust to Solve Dispute | **.35***** | **.36***** | **.43***** | -.08 | -.18 | .03 | **.61***** | 1 | **.78***** | **.59***** |
| Willing to Seek Advice | **.53***** | **.50***** | **.54***** | -.09 | **-.25**** | .06 | **.48***** | **.78***** | 1 | **.64***** |
| Willing to Share Car | **.32***** | **.38***** | **.40***** | .05 | -.11 | .15 | **.28**** | **.59***** | **.64***** | 1 |

Pearson r correlations for measures included in Study 1. *N* = 104.

*. Correlation is significant at the .05 level (2-tailed). **. Correlation is significant at the 0.01 level (2-tailed). ***. Correlation is significant at the .001 level (2-tailed).

**Table D. Correlation Table of Measures Included in Study 3.**

|  | How Principled Target is | How Ethical Target is | | How Morally Upstanding Target is | Dispositional Attribution (base item) | Representativeness of Behavior | Situational Attribution |
| --- | --- | --- | --- | --- | --- | --- | --- |
| How Principled Target Is | 1 | | **.89***** | **.90***** | **.25**** | **.18*** | **.23**** |
| How Ethical Target is | .**89***** | | 1 | **.86***** | **.27***** | **.20*** | **.21*** |
| How Morally Upstanding Target is | **.90***** | | **.86***** | 1 | **.28***** | **.23**** | **.26**** |
| Dispositional Attribution (base item) | **.25**** | | **.27**** | **.28***** | 1 | **.81***** | .16 |
| Representativeness of Behavior | **.18*** | | **.20*** | **.23**** | **.81***** | 1 | .10 |
| Situational Attribution | **.23**** | | **.21*** | **.26**** | .16 | .10 | 1 |

Pearson r correlations for measures included in Study 1. *N* = 142.

*. Correlation is significant at the .05 level (2-tailed). **. Correlation is significant at the 0.01 level (2-tailed). ***. Correlation is significant at the .001 level (2-tailed).
