# Supplementary material for: Separate and unequal: Moral domains differ in corresponding social judgments of others
Source: PLoS One. 2026 Jan 8;21(1):e0338026. doi: 10.1371/journal.pone.0338026 (PMC12782401; doi:10.1371/journal.pone.0338026)
Supplement: S1 Text — (DOCX) [file pone.0338026.s009.docx]

**S1 Text. Study 1 Supplemental Analyses – Domain Comparisons.**

Given the results of Study 1, we wanted to ensure that the variance of judgments based on *Equality* and *Property* behaviors were not due to any peculiar qualities of the domains themselves outside of a social judgment context. Thus, participants were asked to rate the base stimuli for each moral domain on multiple relevant dimensions. These dimensions included the ease with which individuals were able to think of examples of behaviors falling within each domain, their perceived confirmability (i.e., how many times one would require seeing a behavior within each domain before attributing it to a person), the frequency with which one acts in ways that correspond to each domain and the frequency with which they would expect others to do the same, and whether individuals felt each domain was broad or narrow. These dimensions were chosen based on research on personality and trait formation. More specifically, these dimensions comprise attributes and features of traits that have been theorized to lead to differences in how traits are applied and judged. We wanted to assess whether these dimensions of person perception may be different across moral domains in a way that might explain the patterns found in Study 1. We did not expect to find any distinction of *Equality* and *Property* on any of the dimensions tested.

**Method**

**Participants**

We recruited 60 participants from Prolific Academic using the same power calculations as in Study 1. Once again, we aimed to be able to observe small to medium effects for a within-subjects repeated measures ANOVA, *d* = .3. Three participants were excluded from analyses due to failing the cognition check, resulting in a final sample of *N* = 57.

**Procedure**

Participants were told that they would be asked to evaluate various types of behaviors that one might carry out. They were then shown one of 14 behaviors pertaining to our moral domains and asked to evaluate it on each of our dimensions. All behavior descriptions were shown in random order and were not associated with a target person’s name (e.g., “X failed to help a member of their family” instead of “Dave failed to help a member of his family”).

**Materials**

**Moral Domains.** We used the base stimuli for our moral domains in Study 1, split into behaviors consistent with and in violation of each domain, resulting in seven positive and seven negative behaviors total.

**Ease.** Ease in this context refers to the extent to which someone can easily imagine or recall behaviors relating to a moral domain. Literature on personality and the formation of attitudes toward others has suggested that the use of traits to describe others may be directly related to the imaginability of that trait and its accessibility in memory [1][2]. Therefore, we anticipate that the extent to which someone may be able to attribute a moral domain to another individual may be influenced by how easy it may be to think of behaviors within those domains and relevant evaluations.

For each behavior within a moral domain, participants were asked to answer the question “How easy is it to imagine *specific, observable* instances of this type of behavior in everyday life?” on a 5-point scale (1 = *Extremely Difficult*, 5 = *Extremely Easy*).

**Frequency.** Similar to the ease of thinking of behaviors related to a domain and their associated evaluations, moral categories may possess varying levels of accessibility originating from repeated exercise of judgments or acts relating to those categories [2] [3]. We anticipated that more frequently encountered behaviors may lead to more normalization of those behaviors, while nonnormative behaviors may lead to stronger attributions as a function of them sticking out in one’s mind [4].

We asked participants to indicate how often they would expect a specific behavior to be enacted by others and by oneself. This was measured via two items, one asking “In the course of day-to-day life, how frequently would you expect others in the population to [behavior]?” and the other asking “How frequently would you say that *you* [behavior]?”. Both items used a 5-point scale with 1 = *Never* and 5 = *Frequently*. How frequently one encounters or would expect to encounter a particular category of behavior (acted upon by themselves or others) may influence the degree to which associated evaluations may be accessible in memory.

**Confirmability.** When it comes to describing individuals and groups, variations in the extent to which one may confidently confirm the presence or absence of a trait in someone else may impact the impressions formed about them [5] [6]. Behaviors requiring fewer instances to be inferred as part of someone’s character (lower confirmability) should produce stronger character attributions.

Our measure of confirmability was inspired by Rothbart and Park [5]: “Before you would say that someone is the *type of person* who [behavior], how many times would you have to see the person behave that way?”. Participants responded on a 5-point scale, with 1 = *“1 - 2 times”*, 2 = *“3 - 4 times”*, 3 = *“5 - 6 times”*, 4 = *“7 - 8 times”*, and 5 = *“9 - 10 times”*.

**Breadth.** Personality researchers have suggested that behavioral categories may differ in their breadth, or the extent to which individuals may manifest a single behavioral category in different ways [7]. In a social context, applications of traits or behaviors to one’s character may vary as a function of breadth, operationalized in past research as the level of concreteness/specificity of trait categories [8][9] or the number of acts which may fall into them [10]. Broader domains with more diverse behavioral exemplars may produce less coherent and weaker inferences, reducing polarization.

The operationalization for the breadth of moral categories in the current research is in line with that of Hampson and colleagues [11] for personality traits: the conceptual rating of how many behaviors may fall under a moral category and the relative ratings of breadth between pairs of categories. Much in the way that categories of dispositional traits may differ in the inclusivity of potential manifestations, so might categories of moral behaviors. This may have implications for the ease with which one might conceptualize and apply this category to a situation.

We aimed to succinctly ascertain whether some moral domains were perceived as having more behaviors that could fall within their classification than others. To do so, we asked participants to identify whether they perceived the domains as broad or narrow relative to others (cf. [10][11]). Participants were shown the following prompt:

“Some behavioral categories may be broader than others. That is, some categories may feel as though they include a wider range of behaviors that could fall within their boundaries than other categories. Take a look at the categories below and rank them based on how many behaviors you think might fall within their boundaries.

Please keep in mind that you may feel they are all equally broad or equally narrow, and you may put them all into the same Domain.”

Following this prompt, participants were able to select each moral domain and sort it into one of the following categories: Narrow, Neither Broad nor Narrow, or Broad. Each of these categories was assigned a value of 1, 2, and 3, respectively. Importantly, we did not ascertain breadth for both positive and negative behaviors within each domain. Instead, we asked participants to judge breadth using a more holistic description of each domain (e.g., “Helping or failing to help a member of one’s family”).

**Results**

If the distinctiveness of *Equality* and *Property* stemmed from variations in the mechanics of these domains when stripped of a social context, we expected to see similar patterns in participants’ assessments of the domains when not attributed to a social target. However, while there were some differences in these attributional dimensions across our domains, no patterns emerged to suggest that the *Equality* and *Property* domains were distinct in any way that could confound the results of Study 1. All results are included below. These results suggest that the distinct judgments following behaviors in the *Equality* and *Property* domains were not prompted by any peculiarities in the makeup of these domains outside of their use in a social context.

**Table A. Repeated Measures ANOVA Results for Study 1 Supplemental Analyses.**

|  | *SS* | *dfwithin* | *dferror* | *MS* | *F* | η^2^*_p_* |
| --- | --- | --- | --- | --- | --- | --- |
| Ease |  |  |  |  |  |  |
| Domain | 42.89 | 6 | 336 | 7.15 | **9.57***** | .15 |
| Valence | 1.04 | 1 | 56 | 1.81 | .83 | .02 |
| Domain x Valence | 25.61 | 6 | 336 | 4.27 | **6.26***** | .08 |
| Confirmability |  |  |  |  |  |  |
| Domain | 17.63 | 5.57 | 312.06 | 3.16 | **4.26***** | .07 |
| Valence | 15.45 | 1 | 56 | 14.35 | **13.95***** | .20 |
| Domain x Valence | 15.07 | 5.45 | 304.89 | 2.77 | **3.64**** | .06 |
| Frequency (Self) |  |  |  |  |  |  |
| Domain | 9.40 | 6 | 336 | 1.57 | **4.34***** | .07 |
| Valence | 384.61 | 1 | 56 | 384.61 | **141.07**** | .72 |
| Domain x Valence | 179.10 | 5.14 | 287.80 | 34.85 | **33.81***** | .38 |
| Frequency (Others) |  |  |  |  |  |  |
| Domain | 9.45 | 5.66 | 316.87 | 1.67 | **4.79***** | .08 |
| Valence | 51.13 | 1 | 56 | 51.13 | **13.16***** | .19 |
| Domain x Valence | 71.13 | 5.43 | 304.205 | 13.09 | **20.41***** | .27 |
| Breadth of Domain |  |  |  |  |  |  |
| Domain | 17.46 | 6 | 336 | 2.91 | **3.76**** | .06 |

* indicates significance at the p < .05 level. ** indicates significance at the p < .01 level and *** indicates significance at the p < .001 level. In the case of sphericity violations, Hyunh-Feldt adjustments were used.

**Fig A. How Easy is it to Think of Specific, Observable Behaviors in Each Domain?**


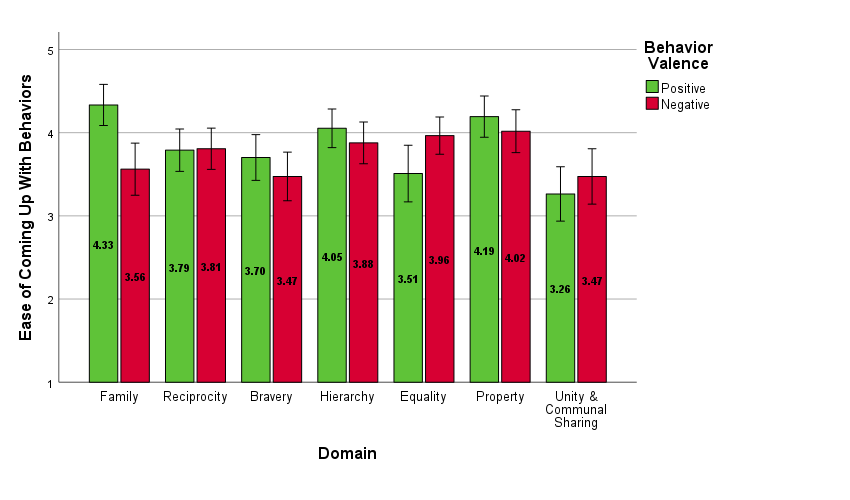


For each domain, participants were asked “How easy is it to imagine specific, observable instances of this type of behavior in everyday life?” from 1 = *Extremely difficult* to 5 = *Extremely easy.* While there were differences across domains, no systematic differences emerged. More specifically, no differences emerged that suggest it is easier to think of examples of behaviors relating to *Equality* and *Property* behaviors over other domains. The Bonferroni-corrected multiple comparisons showing the directions of these effects are included in Table B below.

**Table B. Multiple Comparisons of Ease of Thinking of Domain-Relevant Behaviors.**

| Valence | (I) Domain | (J) Domain | Mean Difference (I-J) | Std. Error | 95% Confidence Interval for Difference^b^ | |  |
| --- | --- | --- | --- | --- | --- | --- | --- |
|  |  |  |  |  | Lower Bound | Upper Bound |  |
| Positive | Family | Reciprocity | **.54^*^** | .15 | .07 | 1.02 |  |
|  |  | Bravery | **.63^*^** | .16 | .12 | 1.15 |  |
|  |  | Hierarchy | .28 | .15 | -.18 | .74 |  |
|  |  | Equality | **.82^***^** | .18 | .24 | 1.41 |  |
|  |  | Property | .14 | .13 | -.27 | .55 |  |
|  |  | Unity & Communal Sharing | **1.07^***^** | .18 | .51 | 1.63 |  |
|  | Reciprocity | Family | **-.54^*^** | .15 | -1.02 | -.07 |  |
|  |  | Bravery | .09 | .13 | -.31 | .49 |  |
|  |  | Hierarchy | -.26 | .17 | -.80 | .27 |  |
|  |  | Equality | .28 | .20 | -.36 | .92 |  |
|  |  | Property | -.40 | .15 | -.89 | .08 |  |
|  |  | Unity & Communal Sharing | .53 | .17 | -.01 | 1.06 |  |
|  | Bravery | Family | **-.63^*^** | .16 | -1.15 | -.12 |  |
|  |  | Reciprocity | -.09 | .13 | -.49 | .31 |  |
|  |  | Hierarchy | -.35 | .18 | -.91 | .21 |  |
|  |  | Equality | .19 | .21 | -.46 | .85 |  |
|  |  | Property | -.49 | .16 | -1.00 | .01 |  |
|  |  | Unity & Communal Sharing | .44 | .19 | -.15 | 1.03 |  |
|  | Hierarchy | Family | -.28 | .15 | -.74 | .18 |  |
|  |  | Reciprocity | .26 | .17 | -.27 | .80 |  |
|  |  | Bravery | .35 | .18 | -.21 | .91 |  |
|  |  | Equality | .54 | .18 | -.03 | 1.11 |  |
|  |  | Property | -.14 | .15 | -.61 | .33 |  |
|  |  | Unity & Communal Sharing | **.79^***^** | .18 | .23 | 1.35 |  |
|  | Equality | Family | **-.82^***^** | .18 | -1.41 | -.24 |  |
|  |  | Reciprocity | -.28 | .20 | -.92 | .36 |  |
|  |  | Bravery | -.19 | .21 | -.85 | .46 |  |
|  |  | Hierarchy | -.54 | .18 | -1.11 | .03 |  |
|  |  | Property | **-.68^***^** | .17 | -1.22 | -.15 |  |
|  |  | Unity & Communal Sharing | .25 | .18 | -.31 | .80 |  |
|  | Property | Family | -.14 | .13 | -.55 | .27 |  |
|  |  | Reciprocity | .40 | .15 | -.08 | .89 |  |
|  |  | Bravery | .49 | .16 | -.01 | 1.00 |  |
|  |  | Hierarchy | .14 | .15 | -.33 | .61 |  |
|  |  | Equality | **.68^***^** | .17 | .15 | 1.22 |  |
|  |  | Unity & Communal Sharing | **.93^***^** | .17 | .40 | 1.46 |  |
|  | Unity & Communal Sharing | Family | **-1.07^***^** | .18 | -1.63 | -.51 |  |
|  |  | Reciprocity | -.53 | .17 | -1.06 | .01 |  |
|  |  | Bravery | -.44 | .19 | -1.03 | .15 |  |
|  |  | Hierarchy | **-.79^***^** | .18 | -1.35 | -.23 |  |
|  |  | Equality | -.25 | .18 | -.80 | .31 |  |
|  |  | Property | **-.93^***^** | .17 | -1.46 | -.40 |  |
| Negative | Family | Reciprocity | -.25 | .14 | -.68 | .19 |  |
|  |  | Bravery | .09 | .19 | -.52 | .69 |  |
|  |  | Hierarchy | -.32 | .14 | -.77 | .14 |  |
|  |  | Equality | -.40 | .14 | -.85 | .04 |  |
|  |  | Property | -.46 | .16 | -.97 | .05 |  |
|  |  | Unity & Communal Sharing | .09 | .16 | -.41 | .59 |  |
|  | Reciprocity | Family | .25 | .14 | -.19 | .68 |  |
|  |  | Bravery | .33 | .17 | -.20 | .86 |  |
|  |  | Hierarchy | -.07 | .11 | -.43 | .28 |  |
|  |  | Equality | -.16 | .12 | -.55 | .24 |  |
|  |  | Property | -.21 | .13 | -.63 | .21 |  |
|  |  | Unity & Communal Sharing | .33 | .14 | -.10 | .76 |  |
|  | Bravery | Family | -.09 | .19 | -.69 | .52 |  |
|  |  | Reciprocity | -.33 | .17 | -.86 | .20 |  |
|  |  | Hierarchy | -.40 | .17 | -.94 | .13 |  |
|  |  | Equality | **-.49^*^** | .15 | -.97 | -.01 |  |
|  |  | Property | **-.54^*^** | .17 | -1.07 | -.01 |  |
|  |  | Unity & Communal Sharing | .00 | .16 | -.51 | .51 |  |
|  | Hierarchy | Family | .32 | .14 | -.14 | .77 |  |
|  |  | Reciprocity | .07 | .11 | -.28 | .43 |  |
|  |  | Bravery | .40 | .17 | -.13 | .94 |  |
|  |  | Equality | -.09 | .13 | -.51 | .34 |  |
|  |  | Property | -.14 | .11 | -.49 | .21 |  |
|  |  | Unity & Communal Sharing | .40 | .15 | -.06 | .87 |  |
|  | Equality | Family | .40 | .14 | -.04 | .85 |  |
|  |  | Reciprocity | .16 | .12 | -.24 | .55 |  |
|  |  | Bravery | **.49^*^** | .15 | .01 | .97 |  |
|  |  | Hierarchy | .09 | .13 | -.34 | .51 |  |
|  |  | Property | -.05 | .13 | -.48 | .37 |  |
|  |  | Unity & Communal Sharing | .49 | .15 | .00 | .98 |  |
|  | Property | Family | .46 | .16 | -.05 | .97 |  |
|  |  | Reciprocity | .21 | .13 | -.21 | .63 |  |
|  |  | Bravery | **.54^*^** | .17 | .01 | 1.07 |  |
|  |  | Hierarchy | .14 | .11 | -.21 | .49 |  |
|  |  | Equality | .05 | .13 | -.37 | .48 |  |
|  |  | Unity & Communal Sharing | **.54^*^** | .17 | .01 | 1.08 |  |
|  | Unity & Communal Sharing | Family | -.09 | .16 | -.59 | .41 |  |
|  |  | Reciprocity | -.33 | .14 | -.76 | .10 |  |
|  |  | Bravery | .00 | .16 | -.51 | .51 |  |
|  |  | Hierarchy | -.40 | .15 | -.87 | .06 |  |
|  |  | Equality | -.49 | .15 | -.98 | .00 |  |
|  |  | Property | **-.54^*^** | .17 | -1.08 | -.01 |  |
| Based on estimated marginal means | | | | | | | |
| *. The mean difference is significant at the .05 level. ***. The mean difference is significant at the .001 level. | | | | | | | |
| b. Adjustment for multiple comparisons: Bonferroni. | | | | | | | |

**Fig B. How Often Participant Engages in Domain-Relevant Behaviors.**


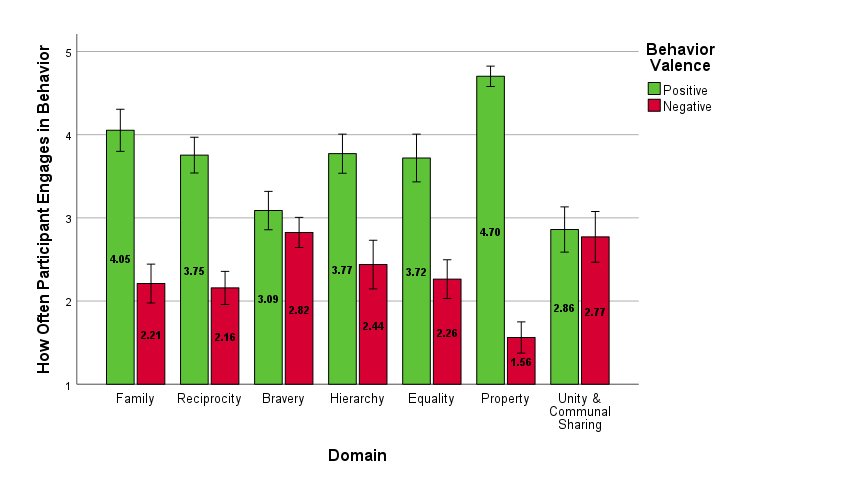


For each domain, participants were asked, “How frequently would you say that you [behavior]?” Responses were on a 5-point scale from 1 = *Never* and 5 = *Frequently*. There were variations across domains (see S6 Table), and Bonferroni-adjusted multiple comparisons (shown in Table C below) show that participants were most likely to engage in Property-relevant behaviors. However, there were no systematic differences among the other domains explaining the results of Study 1.

**Table C. Multiple Comparisons of Participant Engaging in Domain-Relevant Behaviors.**

| Valence | (I) Domain | (J) Domain | Mean Difference (I-J) | Std. Error | 95% Confidence Interval for Difference^b^ | |  |
| --- | --- | --- | --- | --- | --- | --- | --- |
|  |  |  |  |  | Lower Bound | Upper Bound |  |
| Positive | Family | Reciprocity | .30 | .11 | -.07 | .66 |  |
|  |  | Bravery | **.96^***^** | .16 | .45 | 1.47 |  |
|  |  | Hierarchy | .28 | .13 | -.14 | .70 |  |
|  |  | Equality | .33 | .16 | -.17 | .84 |  |
|  |  | Property | **-.65^***^** | .14 | -1.09 | -.21 |  |
|  |  | Unity & Communal Sharing | **1.19^***^** | .18 | .63 | 1.76 |  |
|  | Reciprocity | Family | -.30 | .11 | -.66 | .07 |  |
|  |  | Bravery | **.67^***^** | .12 | .27 | 1.06 |  |
|  |  | Hierarchy | -.02 | .13 | -.44 | .41 |  |
|  |  | Equality | .04 | .15 | -.44 | .51 |  |
|  |  | Property | **-.95^***^** | .11 | -1.31 | -.59 |  |
|  |  | Unity & Communal Sharing | **.89^***^** | .17 | .36 | 1.43 |  |
|  | Bravery | Family | **-.96^***^** | .16 | -1.47 | -.45 |  |
|  |  | Reciprocity | **-.67^***^** | .12 | -1.06 | -.27 |  |
|  |  | Hierarchy | **-.68^*^** | .18 | -1.25 | -.12 |  |
|  |  | Equality | **-.63^*^** | .16 | -1.15 | -.11 |  |
|  |  | Property | **-1.61^***^** | .12 | -2.00 | -1.23 |  |
|  |  | Unity & Communal Sharing | .23 | .16 | -.28 | .74 |  |
|  | Hierarchy | Family | -.28 | .13 | -.70 | .14 |  |
|  |  | Reciprocity | .02 | .13 | -.41 | .44 |  |
|  |  | Bravery | **.68^*^** | .18 | .12 | 1.25 |  |
|  |  | Equality | .05 | .14 | -.39 | .50 |  |
|  |  | Property | **-.93^***^** | .13 | -1.34 | -.52 |  |
|  |  | Unity & Communal Sharing | **.91^***^** | .17 | .38 | 1.45 |  |
|  | Equality | Family | -.33 | .16 | -.84 | .17 |  |
|  |  | Reciprocity | -.04 | .15 | -.51 | .44 |  |
|  |  | Bravery | **.63^*^** | .16 | .11 | 1.15 |  |
|  |  | Hierarchy | -.05 | .14 | -.50 | .39 |  |
|  |  | Property | **-.98^***^** | .15 | -1.45 | -.51 |  |
|  |  | Unity & Communal Sharing | **.86^***^** | .16 | .34 | 1.38 |  |
|  | Property | Family | **.65^***^** | .14 | .21 | 1.09 |  |
|  |  | Reciprocity | **.95^***^** | .11 | .59 | 1.31 |  |
|  |  | Bravery | **1.61^***^** | .12 | 1.23 | 2.00 |  |
|  |  | Hierarchy | **.93^***^** | .13 | .52 | 1.34 |  |
|  |  | Equality | **.98^***^** | .15 | .51 | 1.45 |  |
|  |  | Unity & Communal Sharing | **1.84^***^** | .16 | 1.34 | 2.34 |  |
|  | Unity & Communal Sharing | Family | **-1.19^***^** | .18 | -1.76 | -.63 |  |
|  |  | Reciprocity | **-.89^***^** | .17 | -1.43 | -.36 |  |
|  |  | Bravery | -.23 | .16 | -.74 | .28 |  |
|  |  | Hierarchy | **-.91^***^** | .17 | -1.45 | -.38 |  |
|  |  | Equality | **-.86^***^** | .16 | -1.38 | -.34 |  |
|  |  | Property | **-1.84^***^** | .16 | -2.34 | -1.34 |  |
| Negative | Family | Reciprocity | .05 | .12 | -.33 | .44 |  |
|  |  | Bravery | **-.61^***^** | .13 | -1.02 | -.21 |  |
|  |  | Hierarchy | -.23 | .16 | -.73 | .27 |  |
|  |  | Equality | -.05 | .13 | -.48 | .37 |  |
|  |  | Property | **.65^***^** | .13 | .25 | 1.05 |  |
|  |  | Unity & Communal Sharing | **-.56^*^** | .17 | -1.10 | -.02 |  |
|  | Reciprocity | Family | -.05 | .12 | -.44 | .33 |  |
|  |  | Bravery | **-.67^***^** | .11 | -1.02 | -.32 |  |
|  |  | Hierarchy | -.28 | .16 | -.80 | .23 |  |
|  |  | Equality | -.11 | .14 | -.54 | .33 |  |
|  |  | Property | **.60^***^** | .11 | .25 | .94 |  |
|  |  | Unity & Communal Sharing | **-.61^*^** | .17 | -1.17 | -.06 |  |
|  | Bravery | Family | **.61^***^** | .13 | .21 | 1.02 |  |
|  |  | Reciprocity | **.67^***^** | .11 | .32 | 1.02 |  |
|  |  | Hierarchy | .39 | .15 | -.09 | .86 |  |
|  |  | Equality | **.56^***^** | .13 | .14 | .98 |  |
|  |  | Property | **1.26^***^** | .12 | .88 | 1.65 |  |
|  |  | Unity & Communal Sharing | .05 | .18 | -.52 | .63 |  |
|  | Hierarchy | Family | .23 | .16 | -.27 | .73 |  |
|  |  | Reciprocity | .28 | .16 | -.23 | .80 |  |
|  |  | Bravery | -.39 | .15 | -.86 | .09 |  |
|  |  | Equality | .18 | .16 | -.34 | .69 |  |
|  |  | Property | **.88^***^** | .15 | .40 | 1.36 |  |
|  |  | Unity & Communal Sharing | -.33 | .16 | -.86 | .19 |  |
|  | Equality | Family | .05 | .13 | -.37 | .48 |  |
|  |  | Reciprocity | .11 | .14 | -.33 | .54 |  |
|  |  | Bravery | **-.56^***^** | .13 | -.98 | -.14 |  |
|  |  | Hierarchy | -.18 | .16 | -.69 | .34 |  |
|  |  | Property | **.70^***^** | .13 | .30 | 1.11 |  |
|  |  | Unity & Communal Sharing | -.51 | .17 | -1.04 | .02 |  |
|  | Property | Family | **-.65^***^** | .13 | -1.05 | -.25 |  |
|  |  | Reciprocity | **-.60^***^** | .11 | -.94 | -.25 |  |
|  |  | Bravery | **-1.26^***^** | .12 | -1.65 | -.88 |  |
|  |  | Hierarchy | **-.88^***^** | .15 | -1.36 | -.40 |  |
|  |  | Equality | **-.70^***^** | .13 | -1.11 | -.30 |  |
|  |  | Unity & Communal Sharing | **-1.21^***^** | .17 | -1.74 | -.68 |  |
|  | Unity & Communal Sharing | Family | **.56^*^** | .17 | .02 | 1.10 |  |
|  |  | Reciprocity | **.61^*^** | .17 | .06 | 1.17 |  |
|  |  | Bravery | -.05 | .18 | -.63 | .52 |  |
|  |  | Hierarchy | .33 | .16 | -.19 | .86 |  |
|  |  | Equality | .51 | .17 | -.02 | 1.04 |  |
|  |  | Property | **1.21^***^** | .17 | .68 | 1.74 |  |
| Based on estimated marginal means | | | | | | | |
| *. The mean difference is significant at the .05 level. ***. The mean difference is significant at the .001 level. | | | | | | | |
| b. Adjustment for multiple comparisons: Bonferroni. | | | | | | | |

**Fig C. Expected Frequency of Others Engaging In Domain-Relevant Behaviors.**


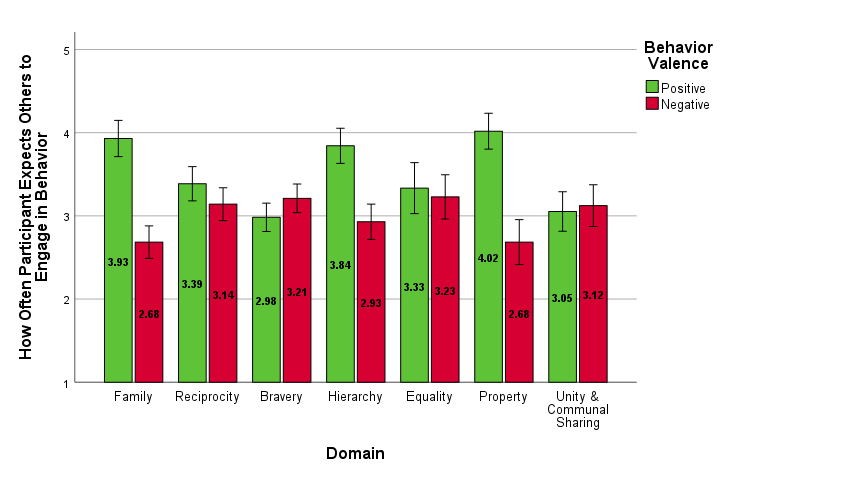


For each domain, participants were asked, “In the course of day to day life, how frequently would you expect others in the population to [behavior]?” Responses were on a 5-point scale from 1 = *Never* and 5 = *Frequently*. Our repeated measures ANOVA showed differences across domains (see S6 Table), but once again no systematic differences. Bonferroni-adjusted multiple comparisons are included below in Table D.

**Table D. Multiple Comparisons of Expected Frequency Others Engage in Domain-Relevant Behaviors.**

| Valence | (I) Domain | (J) Domain | Mean Difference (I-J) | Std. Error | 95% Confidence Interval for Difference^b^ | |  |
| --- | --- | --- | --- | --- | --- | --- | --- |
|  |  |  |  |  | Lower Bound | Upper Bound |  |
| Positive | Family | Reciprocity | **.54^***^** | .11 | .21 | .88 |  |
|  |  | Bravery | **.95^***^** | .11 | .60 | 1.30 |  |
|  |  | Hierarchy | .09 | .13 | -.32 | .50 |  |
|  |  | Equality | **.60^***^** | .13 | .17 | 1.02 |  |
|  |  | Property | -.09 | .13 | -.50 | .32 |  |
|  |  | Unity & Communal Sharing | **.88^***^** | .14 | .44 | 1.31 |  |
|  | Reciprocity | Family | **-.54^***^** | .11 | -.88 | -.21 |  |
|  |  | Bravery | **.40^*^** | .11 | .05 | .76 |  |
|  |  | Hierarchy | **-.46^*^** | .14 | -.89 | -.02 |  |
|  |  | Equality | .05 | .13 | -.36 | .46 |  |
|  |  | Property | **-.63^***^** | .11 | -.98 | -.29 |  |
|  |  | Unity & Communal Sharing | .33 | .14 | -.12 | .79 |  |
|  | Bravery | Family | **-.95^***^** | .11 | -1.30 | -.60 |  |
|  |  | Reciprocity | **-.40^*^** | .11 | -.76 | -.05 |  |
|  |  | Hierarchy | **-.86^***^** | .13 | -1.28 | -.43 |  |
|  |  | Equality | -.35 | .14 | -.81 | .10 |  |
|  |  | Property | **-1.04^***^** | .12 | -1.42 | -.65 |  |
|  |  | Unity & Communal Sharing | -.07 | .13 | -.48 | .34 |  |
|  | Hierarchy | Family | -.09 | .13 | -.50 | .32 |  |
|  |  | Reciprocity | **.46^*^** | .14 | .02 | .89 |  |
|  |  | Bravery | **.86^***^** | .13 | .43 | 1.28 |  |
|  |  | Equality | .51 | .17 | -.02 | 1.04 |  |
|  |  | Property | -.18 | .14 | -.61 | .26 |  |
|  |  | Unity & Communal Sharing | **.79^***^** | .13 | .38 | 1.20 |  |
|  | Equality | Family | **-.60^***^** | .13 | -1.02 | -.17 |  |
|  |  | Reciprocity | -.05 | .13 | -.46 | .36 |  |
|  |  | Bravery | .35 | .14 | -.10 | .81 |  |
|  |  | Hierarchy | -.51 | .17 | -1.04 | .02 |  |
|  |  | Property | **-.68^***^** | .14 | -1.13 | -.24 |  |
|  |  | Unity & Communal Sharing | .28 | .16 | -.23 | .79 |  |
|  | Property | Family | .09 | .13 | -.32 | .50 |  |
|  |  | Reciprocity | **.63^***^** | .11 | .29 | .98 |  |
|  |  | Bravery | **1.04^***^** | .12 | .65 | 1.42 |  |
|  |  | Hierarchy | .18 | .14 | -.26 | .61 |  |
|  |  | Equality | **.68^***^** | .14 | .24 | 1.13 |  |
|  |  | Unity & Communal Sharing | **.96^***^** | .15 | .49 | 1.44 |  |
|  | Unity & Communal Sharing | Family | **-.88^***^** | .14 | -1.31 | -.44 |  |
|  |  | Reciprocity | -.33 | .14 | -.79 | .12 |  |
|  |  | Bravery | .07 | .13 | -.34 | .48 |  |
|  |  | Hierarchy | **-.79^***^** | .13 | -1.20 | -.38 |  |
|  |  | Equality | -.28 | .16 | -.79 | .23 |  |
|  |  | Property | **-.96^***^** | .15 | -1.44 | -.49 |  |
| Negative | Family | Reciprocity | **-.46^***^** | .10 | -.78 | -.14 |  |
|  |  | Bravery | **-.53^***^** | .11 | -.87 | -.19 |  |
|  |  | Hierarchy | -.25 | .10 | -.56 | .07 |  |
|  |  | Equality | **-.54^***^** | .11 | -.89 | -.20 |  |
|  |  | Property | .00 | .11 | -.36 | .36 |  |
|  |  | Unity & Communal Sharing | **-.44^*^** | .11 | -.80 | -.08 |  |
|  | Reciprocity | Family | **.46^***^** | .10 | .14 | .78 |  |
|  |  | Bravery | -.07 | .11 | -.41 | .27 |  |
|  |  | Hierarchy | .21 | .13 | -.20 | .62 |  |
|  |  | Equality | -.09 | .12 | -.46 | .29 |  |
|  |  | Property | **.46^***^** | .11 | .11 | .80 |  |
|  |  | Unity & Communal Sharing | .02 | .10 | -.31 | .34 |  |
|  | Bravery | Family | **.53^***^** | .11 | .19 | .87 |  |
|  |  | Reciprocity | .07 | .11 | -.27 | .41 |  |
|  |  | Hierarchy | .28 | .12 | -.10 | .66 |  |
|  |  | Equality | -.02 | .14 | -.48 | .44 |  |
|  |  | Property | **.53^***^** | .13 | .10 | .95 |  |
|  |  | Unity & Communal Sharing | .09 | .14 | -.36 | .54 |  |
|  | Hierarchy | Family | .25 | .10 | -.07 | .56 |  |
|  |  | Reciprocity | -.21 | .13 | -.62 | .20 |  |
|  |  | Bravery | -.28 | .12 | -.66 | .10 |  |
|  |  | Equality | -.30 | .13 | -.73 | .13 |  |
|  |  | Property | .25 | .14 | -.19 | .68 |  |
|  |  | Unity & Communal Sharing | -.19 | .14 | -.63 | .25 |  |
|  | Equality | Family | **.54^***^** | .11 | .20 | .89 |  |
|  |  | Reciprocity | .09 | .12 | -.29 | .46 |  |
|  |  | Bravery | .02 | .14 | -.44 | .48 |  |
|  |  | Hierarchy | .30 | .13 | -.13 | .73 |  |
|  |  | Property | **.54^***^** | .12 | .17 | .92 |  |
|  |  | Unity & Communal Sharing | .11 | .11 | -.26 | .47 |  |
|  | Property | Family | .00 | .11 | -.36 | .36 |  |
|  |  | Reciprocity | **-.46^***^** | .11 | -.80 | -.11 |  |
|  |  | Bravery | **-.53^***^** | .13 | -.95 | -.10 |  |
|  |  | Hierarchy | -.25 | .14 | -.68 | .19 |  |
|  |  | Equality | **-.54^***^** | .12 | -.92 | -.17 |  |
|  |  | Unity & Communal Sharing | **-.44^***^** | .10 | -.75 | -.13 |  |
|  | Unity & Communal Sharing | Family | **.44^*^** | .11 | .08 | .80 |  |
|  |  | Reciprocity | -.02 | .10 | -.34 | .31 |  |
|  |  | Bravery | -.09 | .14 | -.54 | .36 |  |
|  |  | Hierarchy | .19 | .14 | -.25 | .63 |  |
|  |  | Equality | -.11 | .11 | -.47 | .26 |  |
|  |  | Property | **.44^***^** | .10 | .13 | .75 |  |
| Based on estimated marginal means | | | | | | | |
| *. The mean difference is significant at the .05 level. ***. The mean difference is significant at the .001 level. | | | | | | | |
| b. Adjustment for multiple comparisons: Bonferroni. | | | | | | | |

**Fig D. Confirmability of Behaviors Across Domains.**


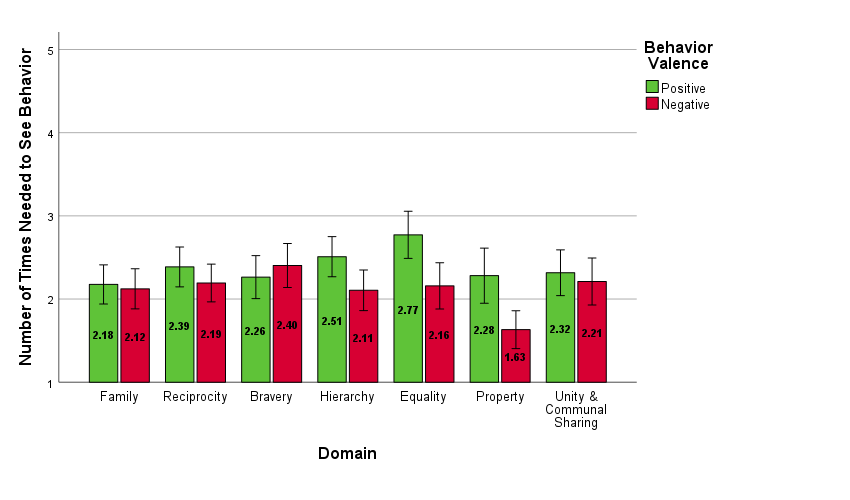


For each domain, participants were asked, “Before you would say that someone is the type of person who [behavior], how many times would you have to see the person behave that way?” Responses were on a 5-point scale from 1 = *1-2 times* and 5 = *9-10 times*. Because a higher value suggests a need to observe more instances of a behavior to attribute it to a social target, lower values indicate higher overall confirmability. Results show *Property*-relevant violations to be statistically different and more confirmable than most other behaviors aside from violations in the *Hierarchy* domain, but this difference would not explain the patterns of judgment shown in Study 1. Bonferroni-adjusted multiple comparisons can be found in Table E below.

**Table E. Multiple Comparisons of Domain Confirmability.**

| Valence | (I) Domain | (J) Domain | Mean Difference (I-J) | Std. Error | 95% Confidence Interval for Difference^b^ | |  |
| --- | --- | --- | --- | --- | --- | --- | --- |
|  |  |  |  |  | Lower Bound | Upper Bound |  |
| Positive | Family | Reciprocity | -.21 | .12 | -.58 | .16 |  |
|  |  | Bravery | -.09 | .13 | -.50 | .33 |  |
|  |  | Hierarchy | -.33 | .12 | -.72 | .05 |  |
|  |  | Equality | **-.60^*^** | .17 | -1.14 | -.05 |  |
|  |  | Property | -.11 | .18 | -.67 | .46 |  |
|  |  | Unity & Communal Sharing | -.14 | .17 | -.67 | .39 |  |
|  | Reciprocity | Family | .21 | .12 | -.16 | .58 |  |
|  |  | Bravery | .12 | .13 | -.28 | .53 |  |
|  |  | Hierarchy | -.12 | .13 | -.55 | .31 |  |
|  |  | Equality | -.39 | .16 | -.88 | .11 |  |
|  |  | Property | .11 | .17 | -.42 | .63 |  |
|  |  | Unity & Communal Sharing | .07 | .16 | -.45 | .59 |  |
|  | Bravery | Family | .09 | .13 | -.33 | .50 |  |
|  |  | Reciprocity | -.12 | .13 | -.53 | .28 |  |
|  |  | Hierarchy | -.25 | .15 | -.73 | .24 |  |
|  |  | Equality | -.51 | .18 | -1.08 | .06 |  |
|  |  | Property | -.02 | .16 | -.54 | .50 |  |
|  |  | Unity & Communal Sharing | -.05 | .18 | -.63 | .52 |  |
|  | Hierarchy | Family | .33 | .12 | -.05 | .72 |  |
|  |  | Reciprocity | .12 | .13 | -.31 | .55 |  |
|  |  | Bravery | .25 | .15 | -.24 | .73 |  |
|  |  | Equality | -.26 | .16 | -.76 | .23 |  |
|  |  | Property | .23 | .18 | -.36 | .81 |  |
|  |  | Unity & Communal Sharing | .19 | .16 | -.30 | .69 |  |
|  | Equality | Family | .60^*^ | .17 | .05 | 1.14 |  |
|  |  | Reciprocity | .39 | .16 | -.11 | .88 |  |
|  |  | Bravery | .51 | .18 | -.06 | 1.08 |  |
|  |  | Hierarchy | .26 | .16 | -.23 | .76 |  |
|  |  | Property | .49 | .17 | -.06 | 1.04 |  |
|  |  | Unity & Communal Sharing | .46 | .18 | -.11 | 1.02 |  |
|  | Property | Family | .11 | .18 | -.46 | .67 |  |
|  |  | Reciprocity | -.11 | .17 | -.63 | .42 |  |
|  |  | Bravery | .02 | .16 | -.50 | .54 |  |
|  |  | Hierarchy | -.23 | .18 | -.81 | .36 |  |
|  |  | Equality | -.49 | .17 | -1.04 | .06 |  |
|  |  | Unity & Communal Sharing | -.04 | .20 | -.67 | .60 |  |
|  | Unity & Communal Sharing | Family | .14 | .17 | -.39 | .67 |  |
|  |  | Reciprocity | -.07 | .16 | -.59 | .45 |  |
|  |  | Bravery | .05 | .18 | -.52 | .63 |  |
|  |  | Hierarchy | -.19 | .16 | -.69 | .30 |  |
|  |  | Equality | -.46 | .18 | -1.02 | .11 |  |
|  |  | Property | .04 | .20 | -.60 | .67 |  |
| Negative | Family | Reciprocity | -.07 | .12 | -.44 | .30 |  |
|  |  | Bravery | -.28 | .17 | -.82 | .26 |  |
|  |  | Hierarchy | .02 | .15 | -.47 | .51 |  |
|  |  | Equality | -.04 | .13 | -.44 | .37 |  |
|  |  | Property | **.49^***^** | .11 | .13 | .85 |  |
|  |  | Unity & Communal Sharing | -.09 | .11 | -.45 | .27 |  |
|  | Reciprocity | Family | .07 | .12 | -.30 | .44 |  |
|  |  | Bravery | -.21 | .16 | -.71 | .29 |  |
|  |  | Hierarchy | .09 | .15 | -.41 | .58 |  |
|  |  | Equality | .04 | .13 | -.39 | .46 |  |
|  |  | Property | **.56^***^** | .11 | .20 | .93 |  |
|  |  | Unity & Communal Sharing | -.02 | .14 | -.46 | .42 |  |
|  | Bravery | Family | .28 | .17 | -.26 | .82 |  |
|  |  | Reciprocity | .21 | .16 | -.29 | .71 |  |
|  |  | Hierarchy | .30 | .17 | -.24 | .84 |  |
|  |  | Equality | .25 | .19 | -.36 | .85 |  |
|  |  | Property | **.77^***^** | .18 | .20 | 1.34 |  |
|  |  | Unity & Communal Sharing | .19 | .19 | -.40 | .79 |  |
|  | Hierarchy | Family | -.02 | .15 | -.51 | .47 |  |
|  |  | Reciprocity | -.09 | .15 | -.58 | .41 |  |
|  |  | Bravery | -.30 | .17 | -.84 | .24 |  |
|  |  | Equality | -.05 | .15 | -.53 | .43 |  |
|  |  | Property | .47 | .15 | -.02 | .97 |  |
|  |  | Unity & Communal Sharing | -.11 | .16 | -.61 | .40 |  |
|  | Equality | Family | .04 | .13 | -.37 | .44 |  |
|  |  | Reciprocity | -.04 | .13 | -.46 | .39 |  |
|  |  | Bravery | -.25 | .19 | -.85 | .36 |  |
|  |  | Hierarchy | .05 | .15 | -.43 | .53 |  |
|  |  | Property | **.53^*^** | .14 | .10 | .96 |  |
|  |  | Unity & Communal Sharing | -.05 | .16 | -.56 | .45 |  |
|  | Property | Family | **-.49^***^** | .11 | -.85 | -.13 |  |
|  |  | Reciprocity | **-.56^***^** | .11 | -.93 | -.20 |  |
|  |  | Bravery | **-.77^***^** | .18 | -1.34 | -.20 |  |
|  |  | Hierarchy | -.47 | .15 | -.97 | .02 |  |
|  |  | Equality | **-.53^*^** | .14 | -.96 | -.10 |  |
|  |  | Unity & Communal Sharing | **-.58^***^** | .14 | -1.01 | -.14 |  |
|  | Unity & Communal Sharing | Family | .09 | .11 | -.27 | .45 |  |
|  |  | Reciprocity | .02 | .14 | -.42 | .46 |  |
|  |  | Bravery | -.19 | .19 | -.79 | .40 |  |
|  |  | Hierarchy | .11 | .16 | -.40 | .61 |  |
|  |  | Equality | .05 | .16 | -.45 | .56 |  |
|  |  | Property | **.58^***^** | .14 | .14 | 1.01 |  |
| Based on estimated marginal means | | | | | | | |
| *. The mean difference is significant at the .05 level. ***. The mean difference is significant at the ,001 level. | | | | | | | |
| b. Adjustment for multiple comparisons: Bonferroni. | | | | | | | |

**Fig E. Perceived Breadth of Moral Domains.**


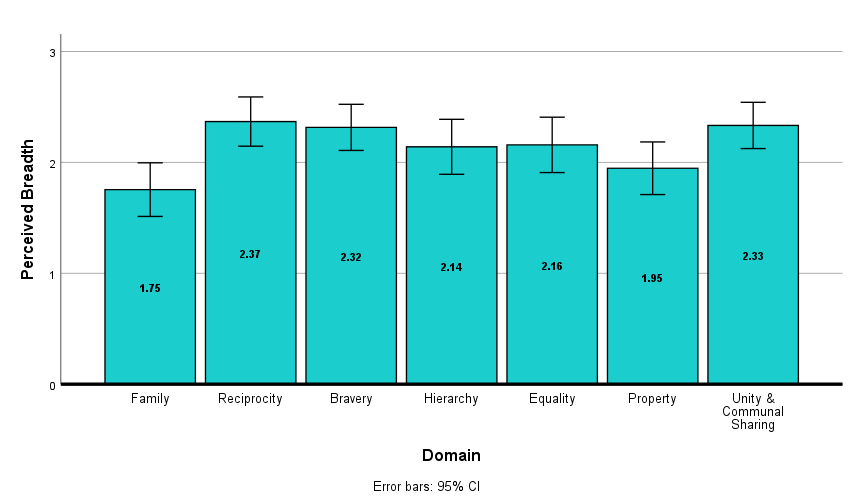


Participants were given the following prompt: “Some behavioral categories may be broader than others. That is, some categories may feel as though they include a wider range of behaviors that could fall within their boundaries than other categories. Take a look at the categories below and rank them based on how many behaviors you think might fall within their boundaries. Please keep in mind that you may feel they are all equally broad or equally narrow, and you may put them all into the same category.” Participants were then asked to sort each of the domains (encompassing both positive and negative behaviors) into one of three categories: 1 = *Narrow*, 2 = *Not Especially Broad* *or Narrow*, or 3 = *Broad*. Bonferroni pairwise comparisons showed that the *Equality* and *Property* domains did not differ meaningfully from any other domains in terms of their perceived breadth (see Table F below).

**Table F. Multiple Comparisons of Domain Breadth.**

| (I) Domain | (J) Domain | Mean Difference (I-J) | Std. Error | 95% Confidence Interval for Difference^b^ | |  |
| --- | --- | --- | --- | --- | --- | --- |
|  |  |  |  | Lower Bound | Upper Bound |  |
| Family | Reciprocity | **-.61^*^** | .17 | -1.15 | -.08 |  |
|  | Bravery | **-.56^*^** | .16 | -1.07 | -.06 |  |
|  | Hierarchy | -.39 | .17 | -.94 | .16 |  |
|  | Equality | -.40 | .19 | -1.00 | .20 |  |
|  | Property | -.19 | .17 | -.74 | .36 |  |
|  | Unity & Communal Sharing | **-.58^*^** | .17 | -1.11 | -.04 |  |
| Reciprocity | Family | **.61^*^** | .17 | .08 | 1.15 |  |
|  | Bravery | .05 | .16 | -.46 | .57 |  |
|  | Hierarchy | .23 | .17 | -.31 | .77 |  |
|  | Equality | .21 | .14 | -.24 | .66 |  |
|  | Property | .42 | .17 | -.13 | .97 |  |
|  | Unity & Communal Sharing | .04 | .14 | -.42 | .49 |  |
| Bravery | Family | **.56^*^** | .16 | .06 | 1.07 |  |
|  | Reciprocity | -.05 | .16 | -.57 | .46 |  |
|  | Hierarchy | .18 | .17 | -.38 | .73 |  |
|  | Equality | .16 | .15 | -.33 | .65 |  |
|  | Property | .37 | .17 | -.16 | .90 |  |
|  | Unity & Communal Sharing | -.02 | .13 | -.44 | .41 |  |
| Hierarchy | Family | .39 | .17 | -.16 | .94 |  |
|  | Reciprocity | -.23 | .17 | -.77 | .31 |  |
|  | Bravery | -.18 | .17 | -.73 | .38 |  |
|  | Equality | -.02 | .18 | -.60 | .57 |  |
|  | Property | .19 | .17 | -.36 | .74 |  |
|  | Unity & Communal Sharing | -.19 | .16 | -.71 | .32 |  |
| Equality | Family | .40 | .19 | -.20 | 1.00 |  |
|  | Reciprocity | -.21 | .14 | -.66 | .24 |  |
|  | Bravery | -.16 | .15 | -.65 | .33 |  |
|  | Hierarchy | .02 | .18 | -.57 | .60 |  |
|  | Property | .21 | .16 | -.29 | .71 |  |
|  | Unity & Communal Sharing | -.18 | .16 | -.68 | .33 |  |
| Property | Family | .19 | .17 | -.36 | .74 |  |
|  | Reciprocity | -.42 | .17 | -.97 | .13 |  |
|  | Bravery | -.37 | .17 | -.90 | .16 |  |
|  | Hierarchy | -.19 | .17 | -.74 | .36 |  |
|  | Equality | -.21 | .16 | -.71 | .29 |  |
|  | Unity & Communal Sharing | -.39 | .17 | -.93 | .16 |  |
| Unity & Communal Sharing | Family | **.58^*^** | .17 | .04 | 1.11 |  |
|  | Reciprocity | -.04 | .14 | -.49 | .42 |  |
|  | Bravery | .02 | .13 | -.41 | .44 |  |
|  | Hierarchy | .19 | .16 | -.32 | .71 |  |
|  | Equality | .18 | .16 | -.33 | .68 |  |
|  | Property | .39 | .17 | -.16 | .93 |  |
| Based on estimated marginal means | | | | | | |
| *. The mean difference is significant at the .05 level. | | | | | | |
| b. Adjustment for multiple comparisons: Bonferroni. | | | | | | |

**References**

1. Hampson SE. Person memory: a semantic category model of personality traits. *Br J Psychol*. 1982;73(1):1-11. doi:10.1111/j.2044-8295.1982.tb01784.x.
2. Schuette RA, Fazio RH. Attitude accessibility and motivation as determinants of biased processing: a test of the MODE model. *Pers Soc Psychol Bull*. 1995;21(7):704-10. doi:10.1177/0146167295217005.
3. Houston DA, Fazio RH. Biased processing as a function of attitude accessibility: making objective judgments subjectively. *Soc Cogn*. 1989;7:51-66.
4. Jones EE, Davis KE, Gergen KJ. Role playing variations and their informational value for person perception. *J Abnorm Soc Psychol*. 1961;63(2):302-10.
5. Rothbart M, Park B. On the confirmability and disconfirmability of trait concepts. *J Pers Soc Psychol*. 1986;50:131–42.
6. Tausch N, Kenworthy JB, Hewstone M. The confirmability and disconfirmability of trait concepts revisited: does content matter? *J Pers Soc Psychol*. 2007;92(3):542-56. doi:10.1037/0022-3514.92.3.542.
7. Hampson SE, John OP, Goldberg LR. Category breadth and hierarchical structure in personality: studies of asymmetries in judgments of trait implications. *J Pers Soc Psychol*. 1986;51(1):37-54. doi:10.1037/0022-3514.51.1.37.
8. Allen RB, Ebbesen EB. Cognitive processes in person perception: retrieval of personality trait and behavioral information. *J Exp Soc Psychol*. 1981;17(2):119-41. doi:10.1016/0022-1031(81)90010-X.
9. Mischel & Peake 1982
10. Rosch E. Principles of categorization. *Psychol Res*. 1978;189-206.
11. Hampson SE. Person memory: a semantic category model of personality traits. *Br J Psychol*. 1982;73(1):1-11. doi:10.1111/j.2044-8295.1982.tb01784.x.
