# Supplementary material for: Separate and unequal: Moral domains differ in corresponding social judgments of others
Source: PLoS One. 2026 Jan 8;21(1):e0338026. doi: 10.1371/journal.pone.0338026 (PMC12782401; doi:10.1371/journal.pone.0338026)
Supplement: Attachment — Submitted filename: Response to Reviewers.docx [file pone.0338026.s010.docx]

Johannes Schwabe

Academic Editor

PLOS ONE

15 Aug 2025

Dear Dr. Schwabe,

On behalf of myself and my co-author, I am writing to resubmit our manuscript, “Separate and Unequal: Moral Domains Differ in Corresponding Judgments of Others” (PONE-D-25-18147), to *PLOS One*.

We greatly appreciate the time you and the reviewers took to provide detailed feedback. Using these comments, we have made revisions to the manuscript that I believe have enhanced its overall quality, with specific attention to adding clarity around our measures and the overall scientific contribution of the work. We have also revised the Supporting Information in line with your requests and those of the reviewers.

The file “Revised Manuscript with Track Changes” shows all changes that were made to the manuscript, which address most feedback that was given. The manuscript also includes an updated link to the primary data and analysis scripts, which have been updated to reflect the addition of gender analyses and minor corrections to the variable names. This link is currently a peer review link but will be made public if the manuscript were to be accepted to *PLOS One*.

Our point-by-point responses to editor and reviewer comments can be seen below, with the original comments included as “**Comment:**” and our response included as “**Response:**”.

Responses to the Editor:

**Editor Comment:** Theoretical Foundation: I agree with both reviewers that the theoretical foundation for your work needs improvement, and this applies to the very grounding of your work all across to final discussion. It is okay to not derive specific hypotheses, but your work does not exist in theoretical vacuum either. Relational models theory, moral foundations theory, and even research on moral identity can be drawn upon in the introduction and speaks to why certain domains might be more or less important in judging others moral character. For example, Haidt speaks about how violations in certain domains can disproportionately undermine global judgments because they signal "core flaws" (e.g., dishonesty). Also consider the work done on differential judgments of deontological versus utilitarian wrong- and right-doers (e.g., Jim Everett's work on this). Your research is currently presented without much of this context, which makes it hard for readers to understand the significance of your work and how it speaks to other findings.

**Response:** Thank you for pointing out the lack of clarity surrounding the theoretical foundation of this work. We included more theory in the introduction of our manuscript, paying special attention to exploring various models of moral domains and discussing these in the context of our research. We specifically added references to Everett and Haidt and spent more time developing the foundation for where our investigation into moral domains begins.

**Editor Comment:** I agree with reviewer 2 that the way you arrive at the domains studied needs to be more transparent. The methods underlying the domain selection are not clear enough, and we should learn a bit more about why two and why exactly those two frameworks were chosen to begin with.

**Response:** Thank you for pointing out this lack of transparency. We added more explanation of our creation of moral stimuli and why we feature a set of domains in our work that may feel potentially limited. Our methods and procedure have been made more transparent.

**Editor Comment:** The authors report the supplementary analysis as evidence that "the patterns cannot account for the results of Study 1". I tend to disagree. There are clear differences, and I feel like these results underline a central problem. Just because the positive and negative variants of a behavior create more polarized ratings on moral characters, why can we infer that the associated domain is more central for moral character judgments? Especially in combination with the fact that the behaviors were not sampled from a representative pool of all behavior potentially representing each domain, but arrived at in a fairly untransparent bottom-up approach, I do not see how we can really infer between-domain comparisons. This needs to be addressed more comprehensively.

**Response:** We agree that the supplemental information for this manuscript was lacking in an elaboration of included variables and why and how they are relevant to the patterns of results in our studies. We have done a complete overhaul of our Supporting Information, including a more in-depth description of the variables included in our supplementary analyses and explanations of those results in the context of our Study 1 results (S1 Text). This, in tandem with providing more explanation of our moral stimuli, should help to illustrate that the differences we found among domains within our studies cannot be explained by differences in features of the domains themselves.

**Editor Comment:** The lack of theorizing and embedding the research in the literature leads to a few issues. For example, in the pre-text to Study 2, the hypothesis appears, that there would be differences in how domain-specific behaviors influence participants' willingness to cooperate with social target. This is apparently purely data-driven, but there is a wealth of literature that would speak to that topic. This also relates to the findings how positive behaviors were perceived as driven more by targets' dispositions than negative behaviors. This conflicts with other studies consistently reporting that violations are given more weight in character judgments. Such apparent contradictions need to be transparent and discussed comprehensively.

**Response:** We have added more discussion surrounding the finding that positive behaviors were perceived as driven more by targets’ dispositions than negative behaviors. This finding may be attributable to the nature of our stimuli and asking participants to judge others on the basis of a singular behavior for each domain and valence, but in addition to this speculation we also included more theory-based reasoning for this finding.

**Editor Comment:** The power analysis of Study 1 is not well-founded. At this point, it is neither clear which exact effect you are interested in, which effect size would be reasonable to expect, neither which statistical test you are going to apply to test it. If this was just a convenience sample with no clear pre-specified hypothesis and power analysis, please just state that. If not, please describe the underlying assumptions more clearly.

**Response:** Thank you for pointing this out. We have added more clarity surrounding the design that was used in our initial power calculation.

**Editor Comment:** The use of difference scores and the exact statistical method planned should be explained in the methods section, and not in the results.

**Response:** We moved this discussion of difference scores to its own subsection within the Methods.

**Editor Comment:** The figures are not self-explanatory. The direction of the differences is not clear.

**Response:** We have revised each included figure using bars where there were previously lines, and added clearer figure labels. We have also revised the figure captions to make the direction of effects in each instance clearer.

**Editor Comment:** Please report tables detailing the Bonferroni-Tests in the supplementary material and refer to them when talking about their results.

**Response:** Our updated Supporting Information includes Bonferroni-adjusted multiple comparisons for each of our analyses to more clearly illustrate differences across our variables (S2, S4, and S6 Appendices). References to these have also been added to the main text where relevant.

**Editor Comment:** When comparing means, report the means and their SD in-text, not just the mean difference and CIs.

**Response:** Where mean differences and CIs were reported in the text, this has been replaced with M and SD.

**Editor Comment:** Generally, please report the individual variables (positive and negative, actor versus situation attribution, etc.) in the supplementary materials, and potentially even replace the current bar charts by dumb bell plots or similar, depicting both individual values as well as the differences.

**Response:** Dumbbell plots of our elaborated analyses not using difference scores have been added to the Supporting Information and referenced in the manuscript where relevant (S1, S3, and S5 Appendices).

**Editor Comment:** A clearer rationale for why we need the cognitive load paradigm is important would make it easier for readers to understand how Study 3 adds to the picture.

**Response:** More discussion of our cognitive load paradigm and its relevance to impressions and social judgment has been added to the manuscript.

**Editor Comment:** Please include standard means and correlations tables for all Studies, where possible based on individual variables, and add to the supplementary material if too clunky for the main manuscript.

**Response:** Standard means tables and correlations tables for each study are included at the end of the Supporting Information (S7 Appendix).

Responses to Reviewers:

**Reviewer Comment:** As I was advancing in the review I missed an introduction to the moral domains and reflections through the studies how and why these moral domains had relatevely lower results then the Property and Equality domain. There was too much focus in these 2 moral domains throughout the study.

**Response:** In addition to elaborating on our domains and stimuli creation, we added separate Discussion sections for each study to address findings incrementally.

**Reviewer Comment:** I liked that you tought carefully in your limitations about the cultural landscape, but I missed the same carefull thinking on the characteristics of your participants (e.g. gender. In study 1 it amounted to 83.3% of the sample. Does this influeced the result?)

**Response:** We have added gender analyses for each study (and the corresponding analysis scripts) to investigate whether the gender composition of our samples may have influenced our results. There was no indication that it did, which we have mentioned for each study in the manuscript.

**Reviewer Comment:**

- No refs. "Different days bring different moral tales..." -> ref here!

- "Still remains unclear..." -> To whom? From which starting point? Ref.

Morality And Social Life

- "Violating the relational..." - ref

**Response:** While these statements may have come off as referencing specific bodies of work, the first two statements are abstract musings of the phenomenon discussed in the manuscript and the gap it addresses. The third statement is continuing from a thought in the preceding sentence in which a relevant reference is included.

**Reviewer Comment:** Ethics and Data availability

- The first link is not well formatted. Remove the linking from the ')'

**Response:** Thank you for pointing this out. The links have been revised to link to an OSF repository for the purpose of peer review and may be made publicly available if the manuscript is accepted.

**Reviewer comment:** Method / Participants

- (Faul et al., 2009) - Ref bad formatted

**Response:** Thank you for pointing this out. This reference has been updated.

**Reviewer comment:** Materials / Moral Domains

- "The current studies used a set of moral domains..." - Why?

**Response:** Thank you for addressing the lack of clarity surrounding the domains. We have addressed this by adding more elaboration surrounding our domain stimuli and their creation.

**Reviewer comment:** Table 1 - Bad formatted, Table 1 should appear right after the reference.

**Response:** Thank you for catching this; this table has been moved up in the manuscript to appear after it is referred to in text.

**Reviewer comment:** Study 2 / Participants

- "Cognition check" - which check?

**Response:** I apologize if this was unclear; the manuscript has been updated to reflect that the same cognition check described in Study 1 was also used in the subsequent studies.

**Reviewer Comment:** In the introduction, the authors state that there are distinct moral domains. However, they do not explain which frameworks exist in this regard and which one(s) they base their research on. More information regarding this is given in the Materials section of Study 1. However, since this information is relevant to all studies and important for understanding the basic idea behind the research, I suggest presenting it in the introduction section. Further information regarding the following questions should be added: Which are the most important frameworks of moral domains? Why were the two (Curry, Chesters, and Van Lissa’s MAC model and Rai and Fiske’s Relational Model) chosen? Why is the research based on both instead of choosing one? How is the theoretical idea underlying the present research (domains differing in their impact on social judgement) linked to the ideas presented in these frameworks? While the authors state that there is no research on whether the domains differ in their impact on social judgement, are there theoretically plausible ideas as to why some domains of the chosen frameworks might be more impactful than others? Adding this information would make a much stronger case for the theoretical idea and its translation into the methodology chosen by the authors.

**Response:** Thank you for this comment. We have added more theory about the existence and development of moral domains to the Introduction section and have also included more detail about our specific domains and stimuli creation in the Materials section for Study 1. This should provide greater overall clarity regarding how our domains were selected and why, as well as how existing frameworks informed our stimuli.

**Reviewer comment:** Furthermore, I am missing some more information on why attributions were chosen as an additional dependent variable. This should be explained in the introduction as a basis for understanding the goal of the present research.

**Response:** Thank you for pointing out that the addition of attribution as an outcome variable was unclear. We have added an explicit explanation to the Introduction that discusses attribution in the context of relevant literature and why it was chosen as a variable.

**Reviewer comment:** When the authors describe in the Materials section of Study 1 how the domains were chosen, it is not clear to me whether this was based on a separate pilot study. This should be made explicit. Furthermore, the authors used a bottom-up approach to choose their moral domains (i.e., having participants generate behaviors pertaining to the domains from the two frameworks from Curry et al. and Rai & Fiske and subsequently choosing and omitting some of the domains based on other participants’ ratings of these behaviors and potential overlaps). Why is this approach more informative for the author’s research question than choosing one of the frameworks and creating behaviors for all domains that are part of this framework? This would have enabled comprehensively examining one full framework instead of having an incomplete picture of both. An explanation of this should be given in the respective section of Study 1 and the potential drawbacks of picking some but not all domains from each of the frameworks (e.g., different scope of the domains from the two frameworks, risk of not covering all potentially relevant moral domains) should be discussed as a limitation in the General Discussion.

**Response:** We have updated the manuscript to reflect that the creation of our stimuli was done in a series of pilot studies. We hope that the addition of more transparency surrounding our stimuli creation process manages to shed light on the reasoning for our creation process and that our intention was to increase the ecological validity of our stimuli, not just to limit our domains to a select few. However, we have also included a statement in our Limitations to acknowledge that any exclusion of domains from contemporary moral models may limit the generalizability of our results.

**Reviewer comment:** While the findings show that there are consistent differences in the effect of the chosen domains on social judgements, the authors did not investigate why some of the domains might be more important for social judgements than others, and the use of some but not all domains from two different frameworks makes it more difficult to deduct potential explanations. As the research is exploratory, the authors do not provide any theoretical explanation as to why some domains should be more impactful than others, either. In the General Discussion, the authors discuss this; yet, the research they cite does not provide a convincing explanation as to why some domains should be *more* impactful than others. They merely emphasize that the Equality and Property domains should be relevant for judgements that people make. An additional study investigating why some domains are more impactful than others could make this research much more informative. I leave it to the Editor to decide whether this is a necessary addition to the present research. If this is not further empirically investigated, I would be interested in a more detailed explanation in the General Discussion as to why the Equality and Property domains might be more impactful than the others.

**Response:** Thank you for pointing out that we had not provided enough theoretical explanations of these effects in our General Discussion. We have updated this section to provide a more detailed review of existing literature that may speak to the importance of these domains in social life.

**Reviewer comment:** According to the study materials for all three studies, an additional two items were assessed together with the attribution items that were reported in the manuscript. Were these originally part of the attribution measurement and left out post hoc (if so why) or were they used to measure a different construct?

**Response:** I apologize if this was confusing. We mentioned in the Materials in Study 1 that the Disposition measure was created from the base item about dispositional attribution combined with an item about how representative the behavior was of the target. These were combined due to their high correlation (see S7 Appendix). The additional item (about whether participants thought the behavior might change over time) was meant to test another construct we were curious about, but it was not relevant to the current package of studies.

**Reviewer comment:** The main text states that that difference scores were calculated for the correspondent inferences. Yet, the y-axis in the graphs is titled Estimated Marginal Means and does not include any negative values.

**Response:** Thank you for pointing this out. We have recreated all of our graphs to include more easily identified values and clearer labels.

**Reviewer comment:** A different term was used for the Equality/Fairness dimension in the main text compared to the graphs. Please consistently use one term.

**Response:** Thank you for your comment about this. We have updated all graphs and code to include the correct term.

**Reviewer comment:** While the preregistration for Study 3 states that there will be “two between-subjects conditions: one condition where cognitive load is increased by presenting moral stimuli alongside a span task, or one with just the moral stimuli presented alone”, both groups in Study 3 were confronted with a digit memorization task. This deviation should be acknowledged in the manuscript and the reason(s) for the deviation should be explained.

**Response:** Thank you for catching this; we have added a brief explanation about why this differs from our preregistration.

**Reviewer comment:** There seems to be a mistake in the following sentence: “We suspected that correspondent inferences, or inferences based on others’ behavior [16], made following domain-specific behaviors might highlight variations in how heavily these domains are considered when judging others” (p. 4, lines 79-81). Please correct it or otherwise make the sentence easier to understand.

**Response:** We have revised this sentence to be clearer and easier to understand. The new sentence is as follows: “We suspected that inferences made about another’s character following domain-specific behaviors, referred to in literature as correspondent inferences [21], might highlight variations in how heavily these domains are considered when judging others”

Thank you again for your careful attention to our manuscript and considerate feedback. Both authors have read and approved of the revised manuscript and hope that it is suitable for publication in *PLOS One*.

Sincerely,

Savannah Adams

Department of Psychology

University of Michigan

Email: snladams@umich.edu
