# Supplementary material for: Separate and unequal: Moral domains differ in corresponding social judgments of others
Source: PLoS One. 2026 Jan 8;21(1):e0338026. doi: 10.1371/journal.pone.0338026 (PMC12782401; doi:10.1371/journal.pone.0338026)
Supplement: Attachment — Submitted filename: Response_to_Reviewers_auresp_2.docx [file pone.0338026.s011.docx]

Corey Cook

Academic Editor

PLOS ONE

27 Oct 2025

Dear Dr. Cook,

On behalf of myself and my co-author, I am writing to resubmit our manuscript, “Separate and Unequal: Moral Domains Differ in Corresponding Judgments of Others” (PONE-D-25-18147), to *PLOS One*.

We greatly appreciate the time you and the reviewers took to provide detailed feedback. We have used these comments to make revisions that have added more clarity to the manuscript and increased its quality. In particular, we have added more clarity surrounding our reasoning for including certain outcome measures and have more fully developed our discussion of our findings.

The file “Revised Manuscript with Track Changes” shows all changes that were made to the manuscript, which address the feedback from you as well as the feedback from Reviewer 2. Below I have included a point-by-point response to these comments with specific references to changes that have been made in the text where appropriate. In line with journal requirements, all citations have been checked for retractions. There is one additional citation included from the last version of the manuscript, which I have also disclosed below.

Our point-by-point responses to editor and reviewer comments can be seen below, with the original comments included as “**Comment:**” and our response included as “**Response:**”.

Responses to the Editor:

**Editor Comment:** If the reviewer comments include a recommendation to cite specific previously published works, please review and evaluate these publications to determine whether they are relevant and should be cited. There is no requirement to cite these works unless the editor has indicated otherwise. 

Please review your reference list to ensure that it is complete and correct. If you have cited papers that have been retracted, please include the rationale for doing so in the manuscript text, or remove these references and replace them with relevant current references. Any changes to the reference list should be mentioned in the rebuttal letter that accompanies your revised manuscript. If you need to cite a retracted article, indicate the article’s retracted status in the References list and also include a citation and full reference for the retraction notice.

**Response:** Thank you for bringing these requirements to our attention. The citations have been checked, and no known retractions have been issued for any of the papers referenced. In the revision process I added an additional citation to the paper to address comments from Reviewer 2. Surrounding citations have been changed to reflect the difference in number order of citations and the reference was added to the reference list.

**Reviewer 2 Comment:** For future revisions, please structure the response letter more clearly: Indicate which points were made by which reviewer and number each of the points made by the reviewers. For any changes you made in the manuscript, please indicate the page numbers and directly quote the changed text. Without doing so, reviewing your changes is very time-consuming and I cannot say with certainty that I have identified each of the (potentially multiple) passages you changed in response to a specific comment.

**Response:** Thank you for your comment. I have attempted to structure this response letter more clearly and include associated page numbers and quoted text where applicable.

**Reviewer 2 Comment:** The authors now mention that attributions are an additional DV in the introduction section. Apart from mentioning that this should play a role in person perception, however, they do not explain how this is linked to their theoretical idea. Why should different moral domains elicit different attributions? Given that attributions are one of the main DVs, this should be given more weight in the introduction.

**Response:** Thank you for this feedback; we have more fully developed the discussion of attribution as a DV by including why asymmetry in attribution across domains might matter to our research question. This is included on page 5 lines 99-109.

“Research has suggested that whether an individual perceives someone else’s behavior to be driven by their character or a situational circumstance may play a role in intuitive moral character judgments and shape beliefs about the kind of person someone is [22] [23]. In particular, dispositionally attributed behaviors are likely to be perceived as in line with someone’s personal beliefs and identity. We aimed to measure whether acts relevant to some moral domains may be more likely to be attributed to one’s character than acts in other domains. Some asymmetry in attribution following immoral acts has been suggested in past research, [24], though this research has primarily focused on how individuals may differently perceive moral norm violations that harm others vs. those that violate norms of purity. In our research, we aimed to investigate potential asymmetry between a larger set of proposed moral domains suggested by contemporary literature without focusing specifically on moral violations.”

**Reviewer 2 comment:** Regarding your discussion of the different effects of the domains: So far, you have not added a suggestion as to why some domains should be *more* impactful than others. As you rightly point out on p. 26, “differences in how these moral behaviors manifest and their functions within social communities may influence how they are used in social impressions.” Yet, in your previous explanations on pp. 25f., you do not contrast the different manifestations or functions of the domains/behaviors corresponding to domains. You simply point out why the Property and Equality domains are relevant to daily life. However, the interesting factor would be: What differentiates them from the other domains? While you cannot answer this question based on your research and much of your research is exploratory to begin with, it would be interesting to at least derive some assumptions on the differences between the domains in the discussion.

**Response:** This is a good point and we have further developed our discussion to speculate more about why these domains might differ from others. As you point out, we are unable to draw specific comparisons between the domains from our current research, but we have included some elaborated discussion on this topic on page 26 line 553-563.

“While our current findings are insufficient for making concrete differentiations between each of the domains, these different literatures help paint a picture of *Equality-* and *Property*-relevant behaviors as related to early socialization and embedded within social systems of development and everyday living. Within an American context, it is possible that these behaviors have evolved to be more ubiquitous and consistently valued than behaviors in other domains, which may be in line with moral research suggesting cultural variation in moral values based on differences in social ecologies and traditions when implementing or teaching moral beliefs [17]. Indeed, while moral behaviors may overall be adaptive solutions to problems of cooperation and relationship regulation within communities [8] [9], differences in how these moral behaviors manifest and their functions within social communities may influence how they are used in social impressions.”

Thank you again for your careful attention to our manuscript and considerate feedback. Both authors have read and approved of the revised manuscript and hope that it is suitable for publication in *PLOS One*.

Sincerely,

Savannah Adams

Department of Psychology

University of Michigan

Email: snladams@umich.edu
